# Mitofusin 2 regulates neutrophil adhesive migration and the actin cytoskeleton

**DOI:** 10.1242/jcs.248880

**Published:** 2020-09-04

**Authors:** Wenqing Zhou, Alan Y. Hsu, Yueyang Wang, Ramizah Syahirah, Tianqi Wang, Jacob Jeffries, Xu Wang, Haroon Mohammad, Mohamed N. Seleem, David Umulis, Qing Deng

**Affiliations:** 1 Department of Biological Sciences, Purdue University, West Lafayette, IN 47907, USA; 2 Department of Agricultural & Biological Engineering, Purdue University, West Lafayette, IN 47907, USA; 3 Weldon School of Biomedical Engineering, Purdue University, West Lafayette, IN 47907, USA; 4 Department of Comparative Pathobiology, Purdue University, West Lafayette, IN 47907, USA; 5 Purdue Institute for Inflammation, Immunology & Infectious Disease, Purdue University, West Lafayette, IN 47907, USA; 6 Purdue University Center for Cancer Research, Purdue University, West Lafayette, IN 47907, USA

**Keywords:** Mitochondria, Chemotaxis, Rac, Zebrafish, Actin, Leukocyte

## Abstract

Neutrophils rely on glycolysis for energy production. How mitochondria regulate neutrophil function is not fully understood. Here, we report that mitochondrial outer membrane protein Mitofusin 2 (MFN2) regulates neutrophil homeostasis and chemotaxis *in vivo*. *Mfn2*-deficient neutrophils are released from the hematopoietic tissue, trapped in the vasculature in zebrafish embryos, and not capable of chemotaxis. Consistent with this, human neutrophil-like cells that are deficient for MFN2 fail to arrest on activated endothelium under sheer stress or perform chemotaxis on 2D surfaces. Deletion of MFN2 results in a significant reduction of neutrophil infiltration to the inflamed peritoneal cavity in mice. Mechanistically, MFN2-deficient neutrophil-like cells display disrupted mitochondria–ER interaction, heightened intracellular Ca^2+^ levels and elevated Rac activation after chemokine stimulation. Restoring a mitochondria–ER tether rescues the abnormal Ca^2+^ levels, Rac hyperactivation and chemotaxis defect resulting from MFN2 depletion. Finally, inhibition of Rac activation restores chemotaxis in MFN2-deficient neutrophils. Taken together, we have identified that MFN2 regulates neutrophil migration via maintaining the mitochondria–ER interaction to suppress Rac activation, and uncovered a previously unrecognized role of MFN2 in regulating cell migration and the actin cytoskeleton.

This article has an associated First Person interview with the first authors of the paper.

## Abstract (precis)

**Summary:** The ER–mitochondria tether maintained by MFN2 fine-tunes Rac activation to regulate neutrophil adhesive migration and adhesion to endothelial cells under shear stress.

## INTRODUCTION

Neutrophils, the most abundant circulating leukocytes in humans, constitute the first line of host defense. Upon stimulation by either pathogen or host-derived proinflammatory mediators, neutrophils are recruited to inflamed tissue using spatially and temporally dynamic intracellular signaling pathways. Activation of the surface receptors, primarily G-protein-coupled receptors (de Oliveira et al., 2016; Gambardella and Vermeren, 2013; Mócsai et al., 2015; Pantarelli and Welch, 2018), leads to the activation of phosphatidylinositol 3-kinase (PI3K), which produces phosphatidylinositol (3,4,5)P3 and activates small GTPases, such as Rac. Rac promotes actin polymerization at the leading edge and drives cell migration (Futosi et al., 2013). In parallel, G-protein-coupled receptors activate phospholipase C, which generates inositol 1,4,5-trisphosphate (IP3) and promotes Ca^2+^ release from intracellular stores (Tsai et al., 2015). Although intracellular Ca^2+^ is a well-characterized second messenger that activates Rac and regulates cell migration in slowly migrating cells (Price et al., 2003), its role in Rac activation in neutrophils is less clear.

Cell migration requires the coordination of multiple cellular organelles, including mitochondria. Mitochondria carry out oxidative phosphorylation to produce ATP, regulate the intracellular redox status and orchestrate the distribution of Ca^2+^, all of which are involved in regulation of cell migration. In addition, mitochondria morphology changes via fusion and fission (Campello and Scorrano, 2010) to adapt to changing metabolic needs under different conditions. Mitochondria fission promotes cell migration by providing mitochondria and ATP at energy-demanding sites such as the protrusion or the uropod (Campello et al., 2006; Zhao et al., 2013).

In neutrophils, mitochondrial biology is unique. The Warburg effect is documented in neutrophils, such that they primarily use glycolysis for ATP generation (Borregaard and Herlin, 1982). Neutrophils have a relative low number of mitochondria, low respiration rates and low electron transport chain enzymatic activity (Peachman et al., 2001). However, disrupting mitochondrial membrane potential by pharmacological inhibitors abolishes chemotaxis of primary human neutrophils (Bao et al., 2015, 2014; Fossati et al., 2003). Although the Junger group demonstrated that extracellular ATP regulates neutrophil chemotaxis *in vitro* (Bao et al., 2015) and *in vivo* (Li et al., 2016), whether mitochondria provide extracellular ATP to regulate neutrophil migration is not known. Only prolonged, but not short-term, treatment with oligomycin, an ATP synthase inhibitor (with possible secondary effects), affects neutrophil migration (Fossati et al., 2003). These reports prompted us to search for mechanisms delineating the role of mitochondria in neutrophil migration outside the realm of ATP or cellular energy (Bi et al., 2014; Schuler et al., 2017; Zanotelli et al., 2018).

Human neutrophils are terminally differentiated and undergo apoptosis within 24 h in culture and thus are not genetically tractable. We have overcome this hurdle by developing a neutrophil-specific knockout platform in zebrafish (Zhou et al., 2018a). The zebrafish is a suitable model for neutrophil research, and its innate immune system is highly conserved with that of humans. In our previous work, we have confirmed the requirement of mitochondrial membrane potential and the electron transport chain in the migration of zebrafish neutrophils (Zhou et al., 2018a). In addition, we have visualized a highly fused and dynamic tubular network of mitochondria in zebrafish neutrophils, which is consistent with a previous report investigating primary human neutrophils (Maianski et al., 2002). Here we present evidence that a mitochondrial outer membrane protein mitofusin 2 (MFN2) regulates Rac activation to coordinate neutrophil adhesion and migration. In addition, we reveal a previously unknown function of MFN2 in regulating the actin cytoskeleton, contributing to the understanding and management of patients with MFN2-related mitochondrial diseases.

## RESULTS

### Neutrophils depleted of *mfn2* accumulate in zebrafish vasculature

**Fig. 1.**
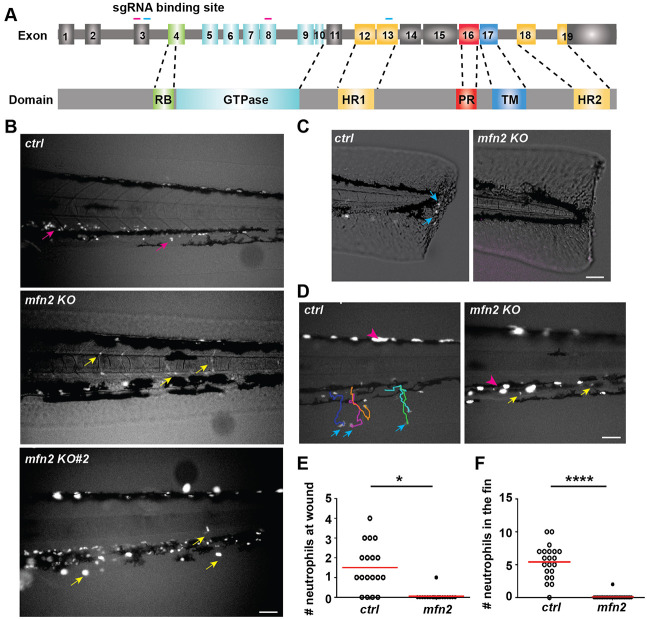
**Mfn2 regulates neutrophil tissue retention and extravasation in zebrafish.** (A) Schematics of the gene structure and protein domains of the zebrafish *mfn2* gene. The first set of sgRNAs (magenta) targets exon 3 and exon 8 in the forward strand, and the second set (blue) targets exon 3 and exon 13 in the forward strand. (B) Representative images of neutrophils in the zebrafish trunk of the indicated transgenic lines at 3 dpf. Magenta arrows, neutrophils in the caudal hematopoietic tissue; yellow arrows, neutrophils in the vasculature. Images are representative of *n*>20 in ctrl and *n*>20 in the *mfn2*-knockout lines. (C,E) Representative images (C) and quantification (E) of neutrophil recruitment to the wound edge at 1 h post wound. Blue arrows, neutrophils migrated to the wound. (D,F) Representative tracks (D) and quantification (F) of neutrophil recruitment to the fin at 30 min post LTB_4_ treatment. Blue arrows, neutrophils in the fin; magenta arrowhead, pigments; yellow arrows, neutrophils in the vasculature. One representative result of three biological repeats is shown in E and F; *n*>20 fish embryos in each group were quantified. *P<0.05, ****P<0.0001 (unpaired *t*-test). Scale bars: 50 µm.

A highly fused and dynamic network of mitochondria in neutrophils has been reported previously (Maianski et al., 2002; Zhou et al., 2018a). To address whether this fused mitochondrial network benefits neutrophil migration, we generated zebrafish transgenic lines with neutrophil specific deletion of proteins that regulate mitochondrial fusion. The mitofusins Mfn1 and Mfn2 are required for mitochondrial outer membrane fusion (Chen et al., 2003), and Opa1 (Dominant optic atrophy 1) regulates inner membrane fusion (Song et al., 2007)*.* In embryos from *Tg(lyzC:Cas9-mfn2 sgRNAs)^pu23^* with *mfn2* deletion in neutrophils, the majority of neutrophils circulate in the bloodstream (Fig. 1A,B; Movie 1). This is in sharp contrast to what is seen in control or the wild-type embryos in which over 99% of neutrophils are retained in the caudal hematopoietic tissue or in the head mesenchyme (Harvie and Huttenlocher, 2015). This abnormal distribution of neutrophils was further confirmed in a second transgenic line expressing different single-guide RNAs (sgRNAs) targeting *mfn2*, *Tg(lyzC:Cas9-mfn2 sgRNAs#2) ^pu24^* (Fig. 1A,B; Movie 2). Neutrophils were sorted from both lines and their respective loci targeted by the four sgRNAs were deep sequenced. The overall mutation frequency ranged from 24% to 60% (Fig. S1A,B). In contrast, circulating neutrophils were not observed in embryos expressing sgRNAs targeting *opa1*, although the velocity of neutrophil migration in the head mesenchyme was significantly reduced (Fig. S1C,D; Movie 3), indicating that the decreased neutrophil retention in tissue is not simply due to defects in mitochondrial fusion.

Next, we determined whether neutrophils in the vasculature were able to respond to acute inflammation induced by a tail transection or perform chemotaxis towards leukotriene B4 (LTB4). Significant defects in both assays were observed in the line with neutrophil-specific *mfn2* deletion (Fig. 1C–F; Movie 4). Taken together, we conclude that *mfn2* regulates neutrophil chemotaxis and extravasation in zebrafish*.*

### MFN2 regulates neutrophil adhesion and migration *in vitro* and *in vivo*

**Fig. 2.**
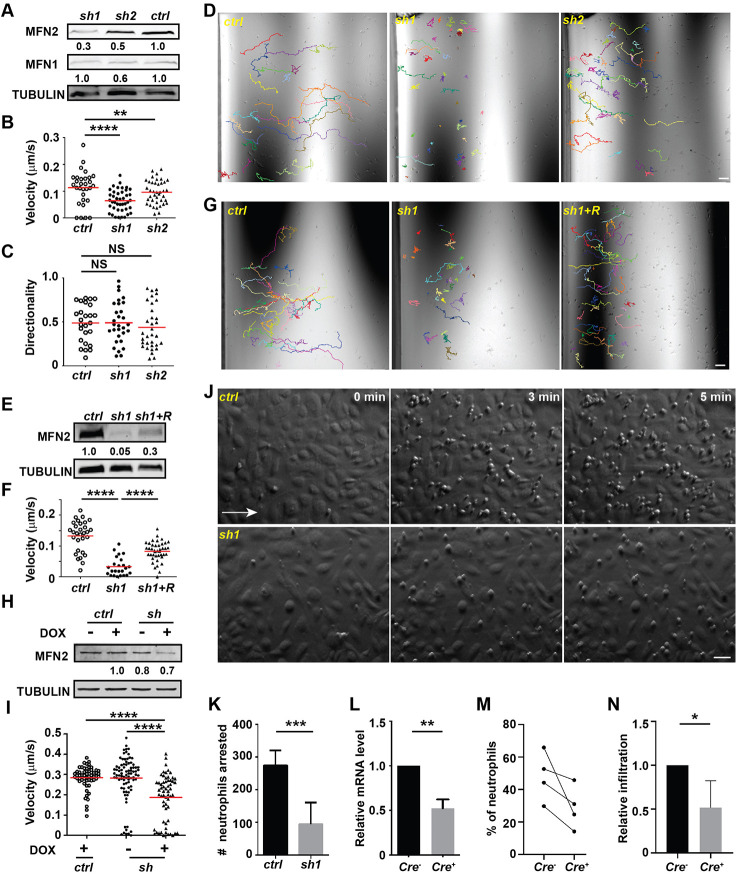
**MFN2 regulates neutrophil migration *in vitro* and *in vivo*.** (A) Western blot determining the expression level of MFN2 and MFN1 in indicated cell lines. *Ctrl*, standard control; *sh1*, shRNA targeting MFN2; *sh2*, a second shRNA targeting MFN2. (B) Quantification of velocity, (C) quantification of directionality and (D) representative images with individual tracks of neutrophil chemotaxis to fMLP. (E) Western blot showing the expression level of MFN2 in indicated cell lines. (F,G) Quantification (F) and representative images (G) with individual tracks of neutrophils migrating toward fMLP. (H) Western blot of MFN2 in indicated cell lines with or without doxycycline induction. (I) Quantification of velocity of neutrophil chemotaxis towards fMLP. (J,K) Adhesion of neutrophils under sheer stress. A HUVEC monolayer was activated with TNFα and neutrophils were flowed on top of the monolayer for 5 min. (J) Representative images showing neutrophils arrested by HUVECs at indicated time points. White arrow, flow direction. (K) Quantification of numbers of neutrophils arrested at 5 min. (L) The relative mRNA level of *Mfn2* in mice neutrophils isolated from *Mfn2^flox/flox^; S100A8:Cre^+^* or the control *Mfn2^flox/flox^; S100A8:Cre^−^* littermates. (M) Percentage of neutrophils in the peritoneal cavity in the indicated mice. (N) Relative neutrophil infiltration to peritoneal cavity. Percentage of neutrophils in the lavage was normalized to that in sex-matched littermates in each experiment. One representative result of three (A–I) or two (J–K) biological repeats is shown. Numbers below each immunoblot indicates the normalized intensity of the bands with the respective loading control and is representative of more than three biological repeats. Data are pooled from two (K), three (L) or four (M and N) independent experiments; *n*>20 cells are tracked and counted in B–D,F,G,I. *P<0.05; **P<0.01; ***P<0.001; ****P<0.0001 [one-way ANOVA (B,C,F,I); unpaired *t*-test (K and L); paired *t*-test (N)]. Scale bars: 50 µm.

To investigate whether the regulation of MFN2 in neutrophil migration is conserved among species, we knocked down MFN2 in the human neutrophil-like cell line HL-60, which can be differentiated to become neutrophil-like cells, denoted dHL-60 cells. Using short-hairpin RNAs (shRNAs), we obtained two individual lines with 70% and 50% reduction of MFN2 after cell differentiation (Fig. 2A). No significant differences in cell viability (indicated by Annexin V staining), differentiation (determined by *MMP9* transcript levels), or surface expression of integrins (CD11b and CD18), a selectin ligand (CD15) and oligosaccharides [wheat germ agglutinin (WGA) staining] were noted (Fig. S2A–G). To investigate whether MFN2 regulates adhesion-dependent neutrophil migration, we used IBIDI chemotaxis slides – microfluidic chambers that allow real-time observation of cell migration towards the chemoattractant on collagen coated two-dimensional (2D) surfaces. Both knockdown lines showed significantly slower chemotaxis towards N-formylmethionyl-leucyl-phenylalanine (fMLP). The directionality was not affected (Fig. 2B–D). The defect in chemotaxis was rescued by reconstitution with a shRNA-resistant *MFN2* in the MFN2-knockdown cells (Fig. 2E–G; Movie 5), supporting the conclusion that the shRNA specifically targets *MFN2*. In addition, we induced expression of a third *MFN2* targeting shRNA in the HL-60 cells at 4 days post differentiation through doxycycline (DOX) treatment, and assayed cell function 2 days later. This acute reduction of MFN2 in dHL-60 cells resulted in similar chemotaxis defects (Fig. 2H,I; Movie 6), suggesting that this defect is not due to nonspecific secondary effects associated with chronic MFN2 depletion. Next, we used a neutrophil flow chamber adhesion assay (Zhou et al., 2014) to measure cell adhesion under shear stress. The majority of *MFN2*-deficient cells failed to adhere firmly to activated endothelial cells (Fig. 2J,K; Movie 7), recapitulating the phenotype in zebrafish where neutrophils depleted of *mfn2* failed to adhere to the vasculature. In addition to cell migration, we determined whether MFN2 regulates other neutrophil functions. Whereas MFN2-deficient dHL-60 cells formed a comparable amount of neutrophil extracellular trap, they were defective in degranulation of the primary granules, although the degranulation of the secondary granules was intact (Fig. S2H–K). Intriguingly, we did not observe any chemotaxis defect in in dHL-60 cells when knocking down MFN1, which shares a similar structure and function to MFN2 (Fig. S3A–C). We have also knocked down OPA1 in HL-60 cells using the same technique as described previously (Amini et al., 2018). Massive cell death and defective cell migration were observed in this line (Fig. S3E–G), possibly due to the depletion of cellular ATP as reported (Amini et al., 2018). To investigate whether MFN2 is required for neutrophil chemotaxis in mammals *in vivo*, we bred *Mfn2* flox/flox mice (Chen et al., 2007) with the *S100A8-Cre* strain (Abram et al., 2013) for neutrophil-specific depletion. With the 50% of *Mfn2* transcript reduction in neutrophils obtained in this strain, a significant reduction of neutrophil infiltration into the inflamed peritoneal cavity was observed (Fig. 2L–N). Consistent with a previous report that *Mfn2* does not regulate blood cell development under homeostatic conditions (Luchsinger et al., 2016), neutrophil frequencies were comparable between the *Cre^+^* and *Cre*^−^ lines (Fig. S2L,M). Therefore, MFN2 is required for neutrophil chemotaxis and infiltration into inflammation sites in mammals *in vivo*.

### MFN2 regulates the actin cytoskeleton and migration of mouse embryonic fibroblasts

**Fig. 3.**
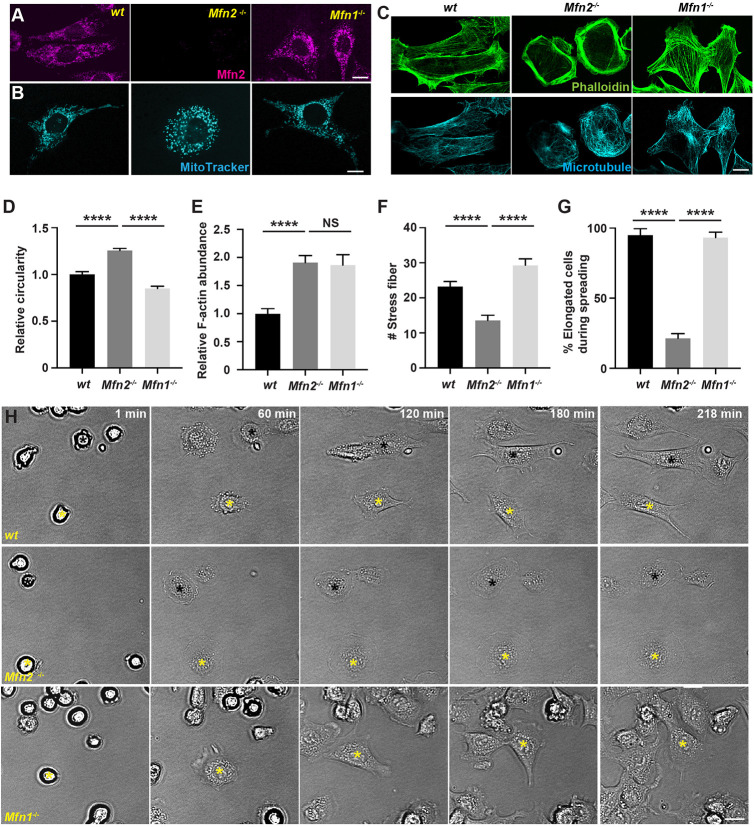
**Mfn2 regulates cytoskeleton organization and cell migration in MEFs.** (A) Immunofluorescence of MFN2 in *wt*, *Mfn2*-null and *Mfn1*-null MEFs. (B) MitoTracker staining in indicated MEFs. (C) Immunofluorescence of microtubule and F-actin (phalloidin) in indicated MEFs. Note, cells in B are different from those in A and C. Quantification of circularity (D), F-actin abundance (E) and number of stress fibers (F) in indicated cells. (G,H) Quantification (G) and representative images (H) of indicated MEFs during cell spreading at indicated time points. Asterisks of yellow and black label the same cells during spreading. Results are mean±s.e.m.; >100 cells quantified in D–G. NS, not significant; ****P<0.0001 (one-way ANOVA). Scale bars: 10 µm (A–C), 200 µm (H).

In addition to neutrophils, we also investigated the role of MFN2 in mouse embryonic fibroblasts (MEFs). MFN2 is well characterized as a mitochondrial fusion mediator in MEFs (Chen et al., 2003; de Brito and Scorrano, 2008; Naon et al., 2016). MFN1, which is structurally similar to MFN2, and also mediates mitochondrial fusion in MEFs (Chen et al., 2003), serves as a specificity control. First, we confirmed specific MFN2 deletion and mitochondria fragmentation in *Mfn2*-null and *Mfn1*-null MEFs (Fig. 3A,B). Next, we investigated the actin cytoskeleton, a major player in adhesive cell migration, using phalloidin staining. Wild-type (*wt*) MEFs were elongated with stress fibers when plated on ligand-coated or uncoated substrates. In contrast, *Mfn2*-null MEFs were round and had an enrichment in actin filaments in the cell cortex with significantly reduced stress fibers in cell body (Fig. 3C–F; Fig. S4A,B). *Mfn1*-deficient MEFs also had increased actin abundance, but were not rounder and still retained stress fibers (Fig. 3C–F). The significant changes in actin organization suggest that *Mfn2*-null MEFs may behave differently to both *wt* and *Mfn1*-null MEFs. Indeed, during cell spreading, *wt* MEFs extended transient filopodia and lamellipodia and eventually elongated, whereas *Mfn2*-null MEFs generated extensive membrane ruffles and retained the circular shape (Fig. 3G,H; Movie 8). *Mfn1*-null cells spread similarly to *wt* cells. Additionally, MEFs deficient for MFN2 migrated slower than *wt* cells during wound closure (Fig. S4C,D; Movie 9). In summary, MFN2 modulates the actin cytoskeleton and cell motility in MEFs.

### MFN2 regulates cell migration through maintaining the mitochondria–ER tether in dHL-60 cells

**Fig. 4.**
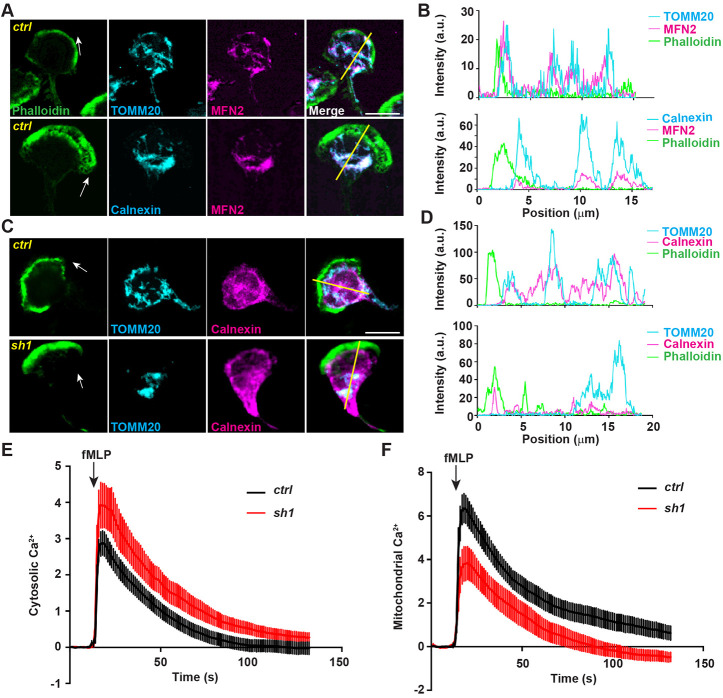
**MFN2 regulates mitochondria-ER interaction.** (A) Immunofluorescence of mitochondria (TOMM20) or ER membrane (calnexin) and MFN2 in indicated cells 3 min post fMLP stimulation. Cells were stained also with phalloidin to reveal F-actin. Arrows, direction of cell polarization. (B) Plot profiles of the fluorescence intensity (MFI) along the corresponding yellow lines in A. a.u., arbitrary units. (C) Immunofluorescence of mitochondria and ER membrane in indicated cells 3 min post fMLP stimulation. Arrows, direction of cell polarization. (D) Plot profiles of the fluorescence intensity (MFI) along the corresponding yellow lines in C. One representative result of three biological repeats was shown in A–D. Scale bars: 10 µm. (E) Cytosolic Ca^2+^ in the control or MFN2-knockdown cell lines after fMLP stimulation. (F) Mitochondrial Ca^2+^ in the control or MFN2-knockdown cell lines after fMLP stimulation. Data are presented as mean±s.d. (*n*>30). One representative result of three biological repeats is shown.

**Fig. 5.**
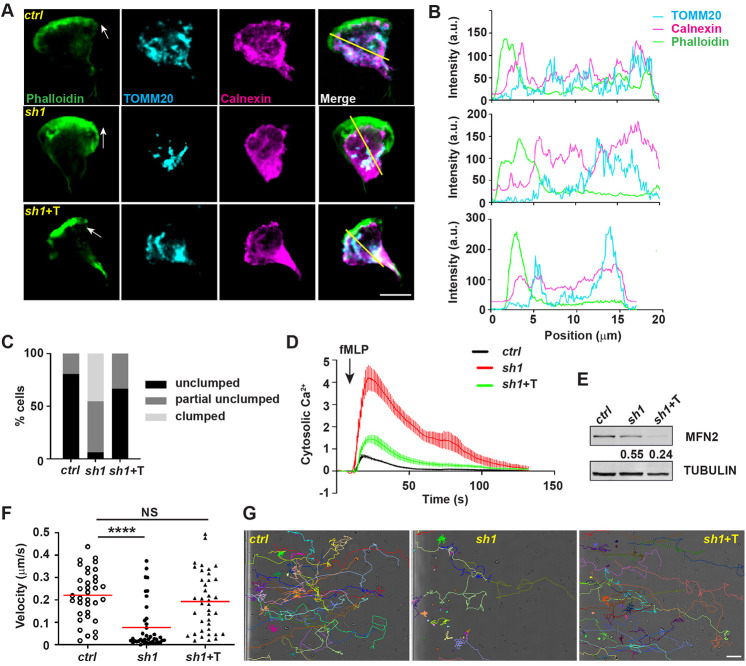
**The mitochondria–ER tether restores neutrophil chemotaxis in MFN2-deficient dHL-60 cells.** (A) Immunofluorescence of mitochondria (TOMM20), and ER membrane (calnexin) in indicated cells 3 min post fMLP stimulation. Arrows, direction of cell polarization. (B) Plot profiles of the fluorescence intensity (MFI) along the corresponding yellow lines in A. a.u., arbitrary units. (C) Quantification of clumped mitochondria in indicated cell lines. *n*=26 (ctrl), *n*=31 (sh1), *n*=42 (sh1+T). (D) Cytosolic Ca^2+^ in the indicated cell lines after fMLP stimulation. Data are presented as mean±s.d. (*n*>30). (E) Western blot of MFN2 in indicated cell lines. sh1+T, HL-60 cells with MFN2-sh1 and synthetic tether construct. Numbers below each immunoblot indicates the normalized intensity of the bands with the respective loading control and is representative of more than three biological repeats. (F,G) Quantification of neutrophil velocity (F) and representative images (G) of individual tracks of neutrophils migrating to fMLP. One representative result of three biological repeats is shown in A,B,D–G. Data are pooled from three independent experiments in C; *n*>20 cells are tracked in F and G. NS, not significant; ****P<0.0001 (one-way ANOVA). Scale bars: 10 µm (A), 100 µm (G).

We have demonstrated a role of MFN2 in regulating cell migration in different model systems. Next, we investigated the underlying molecular mechanisms. We treated dHL-60 cells with a uniform bath of fMLP to induce cell polarization. Consistent with normal directionality during chemotaxis (Fig. 2D), MFN2-defective neutrophils did not have a defect in cell polarization (Fig. 4A). In dHL-60 cells, MFN2 colocalized with both the mitochondria and the endoplasmic reticulum (ER), with Manders' colocalization coefficients of 0.60±0.085 and 0.69±0.13, respectively (mean±s.d.; Fig. 4A,B). Mitochondria also colocalized with the ER (Manders' colocalization coefficient of 0.52±0.097, mean±s.d.) and distributed throughout the cell body (Fig. 4C,D). The morphology of the mitochondria and the ER was further visualized using electron microscopy in dHL-60 cells (Fig. S5). When MFN2 was inhibited, mitochondria lost their structure and formed a cluster in the middle of the cell body (Fig. 4C,D), which did not happen when MFN1 was inhibited (Fig. S3D). However, the mitochondria and the ER Manders' colocalization coefficiency (0.45±0.12, mean±s.d.) was slightly but not significantly reduced when compared with that of the control cells, possibly due to the prevalent ER structure in the cells. The localization of MFN2 in dHL-60s is consistent with that in MEFs, where MFN2 localizes to both the mitochondria and the ER membrane and regulates the tethering of the two organelles (Naon et al., 2016). The close proximity of the ER and the mitochondria regulates multiple cellular signaling pathways including Ca^2+^ homeostasis in MEFs (de Brito and Scorrano, 2008). Indeed, *MFN2*-deficient dHL-60 cells exhibited higher levels of Ca^2+^ in the cytosol and reduced levels in the mitochondria after fMLP stimulation (Fig. 4E,F), suggesting a possible loss of the ER–mitochondria tether in *MFN2*-deficient dHL-60 cells. A positive control for the cytosolic Ca^2+^ measurement is included in Fig. S7A. To further determine whether MFN2 regulates neutrophil chemotaxis through mediating the mitochondria–ER tether, we reconstituted the MFN2 knockdown dHL-60 cells with an artificial tether (Kornmann et al., 2009). The tether is composed of a GFP protein carrying both ER and mitochondrial localization sequences at the ends, which functions independently of MFN2. Indeed, the tether partially restored the morphology and structure of mitochondria in MFN2-deficient dHL-60 cells (Fig. 5A–C). Functionally, the tether expression reduced cytosolic Ca^2+^ levels in the MFN2-deficient cells after fMLP stimulation (Fig. 5D) without increasing MFN2 expression (Fig. 5E). Furthermore, expression of the artificial tether was able to rescue the chemotaxis defect in MFN2-deficient dHL-60 cells (Fig. 5F,G; Movie 10). Taken together, the loss of mitochondria and ER interaction is a possible mechanism for how MFN2 regulates chemotaxis in dHL-60 cells.

We also evaluated other mitochondria-related signals in MFN2-deficient dHL-60 cells. ATP levels were not affected in the MFN2-knockdown dHL-60 cells (Fig. S6A), in line with the observation that mitochondria are not a major source of ATP in neutrophils (Amini et al., 2018; Borregaard and Herlin, 1982). Mitochondrial membrane potential and the reactive oxygen species (ROS) level in mitochondria were slightly reduced when MFN2 was depleted, especially after fMLP stimulation (Fig. S6B–E). This may be due to the altered level of Ca^2+^ in mitochondria, which activates the electron transportation chain (Glancy et al., 2013), whose activity determines the mitochondrial membrane potential and ROS generation. We attempted to chelate cytosolic Ca^2+^ using BAPTA to determine whether elevated cytosolic Ca^2+^ is responsible for the chemotaxis defects in MFN2-knockdown cells. However, a global cytosolic Ca^2+^ inhibition abrogated the ability of dHL-60 to migrate (Fig. S7B,C), possibly due to the requirement for precisely regulated cytosolic Ca^2+^, spatially and/or temporally, for neutrophil migration (Mandeville and Maxfield, 1997; Marks and Maxfield, 1990). We also attempted to reduce cytosolic Ca^2+^ using an IP3R inhibitor, 2APB; however, dHL-60 cells cannot migrate in the presence of this inhibitor (Fig. S7D,E). The mitochondrial Ca^2+^ uniporter (MCU) is one of the major channels for mitochondrial Ca^2+^ uptake, regulating migration of many cell types including primary human neutrophils (Zheng et al., 2017). We further confirmed this observation by treating cells with the MCU inhibitor Ru360 and observed similar results in dHL-60 cells (Fig. S7F,G). As a control, we did not observe a decrease in the protein level of MCU in MFN2-knockdown dHL-60 cells (Fig. S7H), indicating that MFN2 regulates mitochondrial Ca^2+^ uptake in dHL-60 cells independently of the MCU.

### MFN2 suppresses Rac activation in dHL-60 cells

**Fig. 6.**
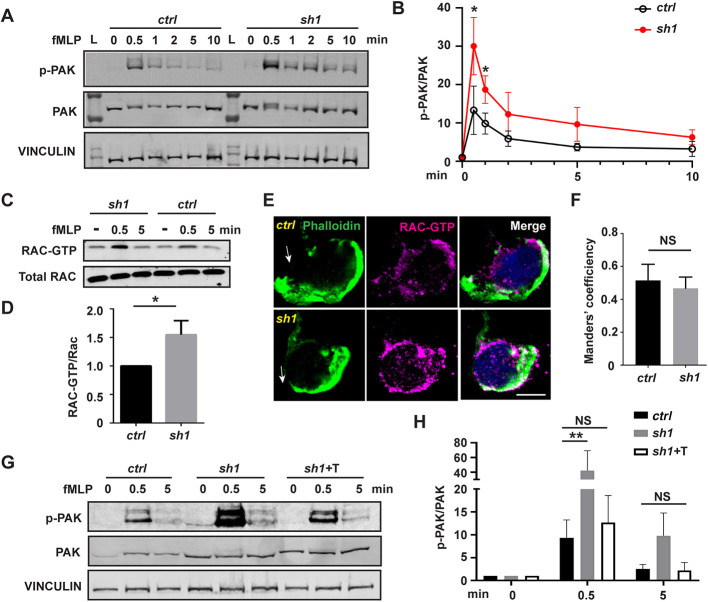
**Heightened Rac activation in MFN2-deficient dHL-60 cells is corrected by inducing a mitochondria–ER tether.** (A) Western blot and (B) quantification determining the amount of phospho-PAK (pPAK) in dHL-60 cells treated with fMLP at indicated time points. L, protein ladder. (C) Western blot determining the amount of Rac-GTP and total Rac protein in dHL-60 cells treated with fMLP at indicated time points. (D) Quantification of Rac activation 5 min after stimulation with fMLP. (E) Immunofluorescence of F-actin and Rac-GTP in indicated cell lines 3 min after stimulation with fMLP. Arrows, direction of cell polarization. (F) Colocalization of Rac-GTP and F-actin. *n*>20. (G,H) Western blot (G) and quantification (H) determining the amount of pPAK in dHL-60 cells treated with fMLP at indicated time points. One representative result of three biological repeats is shown in A,C, and G. Data are pooled from three independent experiments in B, D and H. Error bars represent s.d. NS, non-significant; *P<0.05; **P<0.01 [unpaired *t*-test (B,D), and two-way ANOVA (H)]. Scale bar: 10 µm.

**Fig. 7.**
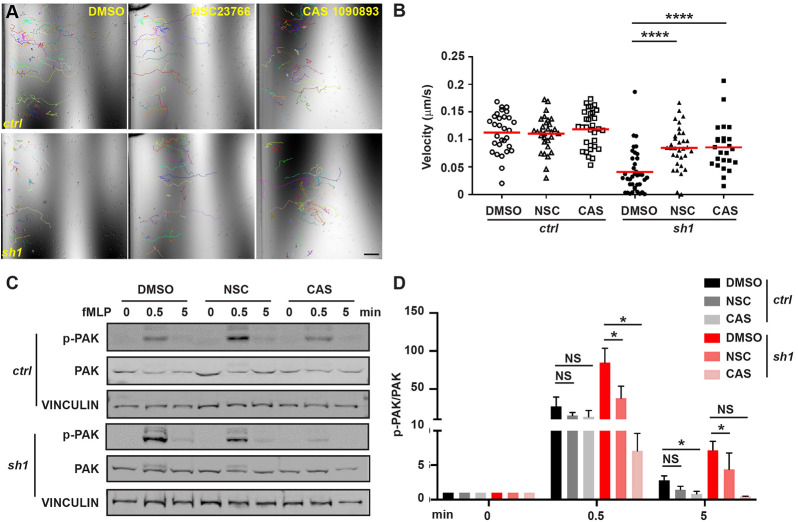
**Heightened Rac activation underlies the chemotaxis defect in MFN2-deficient dHL-60 cells.** (A) Representative images with individual tracks and (B) quantification of velocity of neutrophil chemotaxis towards fMLP in the presence of vehicle or the Rac inhibitor NSC23766 or CAS1090893. (C,D) Western blot (C) and quantification (D) determining the amount of phospho-PAK (pPAK) in dHL-60 cells treated with fMLP and the Rac inhibitors at indicated time points. One representative result of three biological repeats is shown in A–C. Data are pooled from three independent experiments in D. *n*>20 cells are tracked in B. NS, non-significant; *P<0.05; ****P<0.0001 (two-way ANOVA). Scale bar: 100 µm.

Notably, the predominant cortical actin and extensive membrane ruffles seen in *Mfn2*-null MEF cells (Fig. 3) resembled the classic phenotype seen in fibroblasts expressing constitutively active Rac (Hall, 1998), indicating that Rac might be overactivated in MFN2-depleted cells. To test this hypothesis, we measured Rac activation in MFN2-knockdown cells. Cells were plated on substrate-coated plates and stimulated with chemoattractant. The phosphorylation of PAK (PAK1, -2 and -3) (Graziano et al., 2017) was used as a readout for Rac activation. The phosphorylation of PAK peaked at 30 s post stimulation and returned to the baseline at 5 min post stimulation in control cells. However, in MFN2-deficient cells, the phosphorylation of PAK was elevated at multiple time points investigated compared with the control (Fig. 6A,B), suggesting a suppressive role of MFN2 in Rac activation in dHL-60 cells. To further confirm this observation, we performed a Rac-GTP pulldown assay to directly measure active Rac in MFN2-deficient dHL-60 cells. Consistent with the above results, a significantly increased amount of active Rac was detected in MFN2-sh1 cells (Fig. 6C,D). To visualize the subcellular localization of active Rac, we stained Rac-GTP as previously reported (Fayngerts et al., 2017). Rac-GTP colocalizes with F-actin at cell protrusions as well as the retracting rear of the cells, and this colocalization was not affected by MFN2 depletion (Fig. 6E,F). Notably, the excessive phosphorylation of PAK can be corrected by expressing the artificial tether in MFN2-deficient dHL-60 cells (Fig. 6G,H), indicating that MFN2 moderates Rac activation via regulating the mitochondria–ER tether. To determine whether the heightened activation of Rac is the mechanism leading to the chemotaxis defect in MFN2-deficient cells, we treated the cells with two different Rac inhibitors, NSC23766 and CAS1090893. Both inhibitors restored neutrophil migration in *MFN2-sh1* cells, at least partially, and did not affect chemotaxis of control cells (Fig. 7A,B; Movie 11). Additionally, the inhibitors corrected the Rac hyperactivation seen in the *MFN2-sh1* cells, without impacting Rac activation in the control cells (Fig. 7C,D). Taken together, the mitochondria–ER tether maintained by MFN2 regulates mitochondrial Ca^2+^ uptake and Rac signaling to orchestrate chemotaxis in dHL-60 cells.

## DISCUSSION

Here, we report that MFN2 is crucial for neutrophil adhesion and migration, providing evidence that MFN2 regulates the actin cytoskeleton and cell migration. By maintaining the tether between the mitochondria and ER, MFN2 orchestrates intracellular Ca^2+^ signaling and regulates Rac activation. Therefore, we have identified the mechanism for how MFN2 regulates neutrophil adhesive migration, and highlighted the importance of mitochondria and their contact with the ER in neutrophils.

MFN1 and MFN2 both mediate mitochondrial outer membrane fusion (Chen et al., 2003). In MEFs, mitochondria are fragmented in both MFN1- and MFN2-knockout cells. We observed that only in MFN2-knockout MEFs do cells display a Rac overactivation morphology, suggesting that unique functions of MFN2, such as regulating the mitochondrial–ER tether, are relevant in regulating Rac signaling. In neutrophil-like dHL-60 cells, again, only MFN2, and not MFN1, is required for cell migration. Although MFN2 is not the only protein that can maintain the mitochondria–ER tether (Eisenberg-Bord et al., 2016), mitochondria and ER interaction was significantly reduced upon MFN2 deletion in dHL-60 cells, suggesting that MFN2 is at least one of the critical tether proteins in neutrophils. The literature thus far suggests that mitochondrial fission promotes cell migration in many cell types (Campello et al., 2006; Zhao et al., 2013). Here, we propose an alternative model that, in neutrophils, the interaction of mitochondria with the ER is critical in regulating cell migration. It remains to be determined whether mitochondrial fission/fusion regulates neutrophil migration. The fused mitochondrial network in neutrophils is possibly a result of the abundant expression of the mitofusins. Indeed, unlike in MEFs, a significant increase in mitochondria fragmentation was not observed in either the MFN1- or MFN2-knockdown cells (Fig. 4C; Fig. S3D). Evaluating the role of other mitochondrial shape-regulating genes in neutrophil migration will be necessary to draw a solid conclusion on this topic.

Mutations in human MFN2 cause Charcot–Marie–Tooth disease type 2A (CMT2A), a classical axonal peripheral sensorimotor neuropathy (Züchner et al., 2004). MFN2 is also implicated in many other diseases such as cancer, cardiomyopathies, diabetes and Alzheimer's disease (Filadi et al., 2018). Currently, over 100 dominant mutations in the *MFN2* gene have been reported in CMT2A patients (Stuppia et al., 2015), although how these mutations lead to disease is largely unknown. The challenges in MFN2 research are that MFN2 regulates mitochondrial fusion and a plethora of cellular functions, such as mitochondrial dynamics, transport, mtDNA stability, lipid metabolism and survival (Chandhok et al., 2018). In addition, gain-of-function and loss-of-function mutations are reported that affect different aspects of cellular functions (Chandhok et al., 2018). Our findings provide a new direction to understand the consequences of MFN2 deficiency in disease pathology, namely the actin cytoskeleton and Rac activation. Our findings also imply a possibility that the defects in immune cell migration in humans may affect immunity or chronic inflammation, and indirectly regulate the progression of the aforementioned diseases. Future work will be required to carefully evaluate the individual mutations of MFN2 identified in human diseases for their effect on immune cell migration. It is possible that mutations disrupting the mitochondria–ER tether would result in defects in cell adhesion and the cytoskeleton regulation.

Our conclusions present a significant departure from the prevailing focus of bioenergy, or ATP, in cell migration. In many cell types, including neutrophils, the relevance of mitochondria-derived ATP in cell migration is emphasized (Bao et al., 2015, 2014). A recent report has confirmed the findings from established literature that mitochondria do not provide ATP in neutrophils (Amini et al., 2018). Intriguingly, OPA1 deletion suppresses the production of neutrophil extracellular traps and alters the cellular ATP levels by indirectly suppressing glycolysis. In contrast, MFN2 deletion does not affect ATP levels (Fig. S6) nor neutrophil extracellular trap formation (Amini et al., 2018), again suggesting distinct biological functions of OPA1 and MFN2. In vascular endothelial cells, mitochondria also serve as signaling rather than energy-producing moieties (Lugus et al., 2011). In our study, in addition to the altered Ca^2+^ level, mitochondrial membrane potential and ROS, both of which are critical for neutrophil chemotaxis and migration (Fossati et al., 2003; Zhou et al., 2018a), were reduced in stimulated *MFN2*-deficient dHL-60 cells. It remains to be determined whether the modest decreases in mitochondria membrane potential or ROS contribute to the defect in neutrophil migration seen upon MFN2 depletion.

Our result in leukocytes is consistent with previous results in murine fibroblasts (de Brito and Scorrano, 2008) showing that knocking out Mfn2 results in excessive cytosolic Ca^2+^ and defective mitochondrial Ca^2+^ uptake. Intriguingly, chronic blockade of mitochondrial Ca^2+^ import by depleting the MCU results in the reduction of the ER and cytosolic Ca^2+^ pools and a migration defect (Prudent et al., 2016). Although the phenotype is similar upon MFN2 depletion, the underlying mechanisms is possibly different. Whereas MFN2 reduces the cytosolic Ca^2+^ levels after the activation of the chemokine receptor, the MCU is required for maintaining the Ca^2+^ store and elevates the cytosolic Ca^2+^, suggesting a requirement for delicate and precise Ca^2+^ signaling in orchestrating neutrophil migration. Although cytosolic Ca^2+^ triggers the activation of Rac in slow moving cells (Price et al., 2003), previous work in neutrophils suggests that Rac activation is independent of cytosolic Ca^2+^ (Geijsen et al., 1999). This discrepancy could be explained through the differences in assay conditions (suspension versus adhesion) or in how Ca^2+^ levels were manipulated (elevation versus reduction). Further work will be required to determine whether/how elevated Ca^2+^ regulates Rac activation in neutrophils.

In summary, combining evidence from different models, we have identified an essential role for MFN2 in neutrophil adhesion and migration, and determined the downstream mechanism, which provides insights and potential therapeutic strategies for inflammatory diseases and mitochondrial diseases.

## MATERIALS AND METHODS

### Reagents

RPMI-1640 (10-041-CV), DMEM (10-013-CV), HEPES (25-060-CI), Trypan Blue solution (25-900-CI) were from Corning (Corning, NY, USA). Endothelial cell growth medium (CCM027) was from R&D Systems (Minneapolis, MN, USA). Fetal bovine serum (FBS; ES-009-B) and pLKO.1 lentiviral constructs with shRNA were from Millipore Sigma (Burlington, MA, USA). e-Myco plus Mycoplasma PCR detection kit (25234) was from Bulldog Bio (Portsmouth, NH, USA). DMSO (AC610421000) was from ACROS Organics (NJ, USA). Sodium bicarbonate solution (S8761) and sodium pyruvate solution (S8636) was from Sigma-Aldrich (Burlington, MA, USA). Bovine serum albumin (BP1600-100) was from ThermoFisher Scientific (Waltham, MA, USA). The shERWOOD UltramiR lentiviral inducible shRNA system was from Transomics (Huntsville, AL, USA). Lipofectamin 3000 (L3000015) was from ThermoFisher Scientific. Lenti-X concentrator (631232) was from Takara Bio (Mountain View, CA, USA). Primers were from Integrated DNA Technologies (Coralville, IA, USA). Plasmids YFP (#28010), pCMV-dR8.2 dvpr (#8455) and pCMV-VSV-G (#8454) were from Addgene (Watertown, MA, USA). The In-Fusion HD Cloning Plus kit (638920) was from Takara Bio (Mountain View, CA). The RBC lysis solution (158904), QIAamp DNA Mini Kit (51306) and RNeasy mini kit (74104) were from Qiagen (Waltham, MA, USA). Poly(dA:dT) naked was from InvivoGen (San Diego, CA, USA). The Transcriptor first strand cDNA synthesis kit (04379012001) and FastStart Essential DNA Green Master (06402712001) were from Roche (Indianapolis, IN, USA), and the ATP assay kit (ab83355) was from Abcam (Cambridge, MA, USA). The Rac1 pull-down activation assay biochem kit (bead pull-down format) (BK035) was from Cytoskeleton (Denver, CO, USA). The neutrophil isolation kit, mouse (130-097-658) was from Miltenyi (Somerville, MA, USA). µ-slides (80322) and µ-slide 8 well plates (80826) were from IBIDI (Fitchburg, WI, USA), 35 mm plates (430165) and FALCON 96-well plates (353075) were from Corning (Corning, NY, USA), and black wall 96-well plates (655096) were from Greiner Bio-One (Monroe, NC, USA). WGA–Alexa Fluor 594 (W11262), ER-tracker (E34251), TMRM (T668), MitoTracker (M22426), Hoechst 33342 (62249), MitoSOX (M36008), phalloidin–Alexa Fluor 488 (A12379), Sytox Green (S7020), DAPI (D3571), Rhod-2 (R1245MP) and the Fluo-4 calcium imaging kit (F10489) were from ThermoFisher Scientific. Fibrinogen (F3879), doxycycline (D9891), polybrene (TR-1003-G), PMA (P1585), NSC23766 (SML0952), CAS 1090893 (553511), RU360 (557440), TCA (T6399), NaF (G9422), β-glycerophosphate (S6776), thioglycollate (B2551) and fMLP (F3506) were from Millipore Sigma. 2-APB (1224) was from Tocris Bioscience (Minneapolis, MN, USA). Ionomycin (BP25271), puromycin (A1113803) and human TNF-α (PHC3015) was from ThermoFisher Scientific. Antibodies for immunoblotting were: anti-MFN1 (14793S), anti-MFN2 (9482S), anti-Rac1/2/3 (2465S), anti-phospho-PAK (2605S), anti-PAK (2604) and anti-MCU (14997) from Cell Signaling (Danvers, MA, USA); anti-vinculin (V9193) from Millipore Sigma. Secondary antibodies anti-rabbit (SA5-35571) and anti-mouse (Invitrogen 35518) from ThermoFisher Scientific. 2× Laemmli sample buffer (1610737) was from (BIO-RAD (Hercules, CA, USA). Nitrocellulose membranes (9680617) were from LI-COR (Lincoln, NE, USA). Antibodies for immunofluorescence were: anti-MFN2 (9482S) and anti-calnexin (2433S) from Cell Signaling (Danvers, MA, USA); anti-TOMM20 (sc-17764) from Santa Cruz Biotechnology (Dallas, TX, USA); anti-tubulin (T5168) from Millipore Sigma; anti-paxillin (AHO0492) from ThermoFisher Scientific; anti-RAC-GTP (26903) from NewEast Biosciences (King of Prussia, PA, USA). Secondary antibodies were: anti-rabbit-IgG conjugated to Alexa Fluor 568 (A-11011) and anti-mouse-IgG conjugated to Alexa Fluor 647 (A21236) from ThermoFisher Scientific. Antibodies for flow cytometry were: murine anti-CD11b (562605), murine anti-Ly6G (560601), anti-CD63 (B561982) and anti-CD66 (B562741), anti-CD11b (557686), anti-Ly6G (566453), anti-CD18 conjugated to PE (B555924), PE isotype control (B554680), CD15-BV510 (B563141), BV510 isotype control (B562946) were from BD Biosciences (San Jose, CA, USA), and CD11b-AF647 (301319) and AF647 isotype control (400130) from Biolegend (San Diego, CA, USA). Annexin V (563973) was from BD Biosciences (San Jose, CA, USA).

### Animals

The zebrafish (*Danio rerio*) and mice (*Mus musculus*) experiments were conducted in accordance to the internationally accepted standards. The Animal Care and Use Protocols were approved by The Purdue Animal Care and Use Committee (PACUC), adhering to the Guidelines for Use of Zebrafish and Mice in the NIH Intramural Research Program (Protocol number: 1401001018 and 1803001702). MATLAB and the sampsizepwr function was used to calculate the sample sizes required for each experiment based on conservative estimates for the variability in the controls for each type of experiments, with a power of 0.9 (significance level of 0.05) in a two-sample *t*-test. Data were quantified blindly by an investigator not involved in data collection.

To generate transgenic zebrafish lines, plasmids with the tol2 backbone were co-injected with Tol2 transposase mRNA into embryos of the AB strain at one-cell stage as described previously (Zhou et al., 2018a). Constructs for neutrophil-specific knockout in zebrafish were generated as described previously (Zhou et al., 2018a) using the following primers (sgRNA sequences are indicated with underscores):

mfn2 guide1-F1: 5′-GTGGATGAGCTGCGGGTGGGTTTAAGAGCTATGCTGGAAACAGCATAGC-3′; mfn2 guide1-R1: 5′-CGCACCTCCGCCACCTGCCCGAACTAGGAGCCTGGAGAACTGC-3′; mfn2 guide1-F2: 5′-GGTGGCGGAGGTGCGGTTTAAGAGCTATGCTGGAAACAGCATAGC-3′; mfn2 guide1-R2: 5′-CCGCAGCTCATCCACCGAACCAAGAGCTGGAGGGAGA-3′; mfn2 guide2-F1: 5′-GGGGGATACCTGTCCAAAGGTTTAAGAGCTATGCTGGAAACAGCATAGCAAG-3′; mfn2 guide2-R1: 5′-AGACCTTCCTCTATGTGCCCGAACTAGGAGCCTGGAGAACTGCTATATAAAC-3′; mfn2 guide2-F2: 5′-CATAGAGGAAGGTCTGTTTAAGAGCTATGCTGGAAACAGCATAGCAAGTTTAAATAAG-3′; mfn2 guide2-R2: 5′-GGACAGGTATCCCCCCGAACCAAGAGCTGGAGGGAGAGCTATATATAC-3′; opa1 guide-F1: 5′-GTAGTTGGGGACCAGAGTGGTTTAAGAGCTATGCTGGAAACAGCATAGC-3′; opa1 guide-R1: 5′-CCTCAGCTCAGCTGCCCGAACTAGGAGCCTGGAGAACTGC-3′; opa1 guide-F2: 5′-AGCTGAGCAGTGAGGGTTTAAGAGCTATGCTGGAAACAGCATAGC-3′; opa1 guide-R2: 5′-CTGGTCCCCAACTACCGAACCAAGAGCTGGAGGGAGA-3′.

All mice used in this study were purchased from Jackson Laboratories (Bar Harbor, Maine, USA). Conditional *Mfn2* knockout mice (B6.129(Cg)-*Mfn2^tm3Dcc^*/J) were crossed to S100A8-Cre (B6.Cg-Tg(S100A8-cre, -EGFP)1Ilw/J) transgenic mice to obtain a homozygous floxed *Mfn2* alleles with or without the Cre. All mice were used at age 6–8 weeks, and both male and female were used for experiments.

### Cell culture

HEK293T (CRL-11268), wild-type (CRL-2991), *Mfn2*-null (CRL-2993) and *Mfn1*-null (CRL-2992) MEFs were from the American Type Culture Collection (ATCC, Manassas, VA, USA). HUVECs (200P-05N) were from Sigma-Aldrich (St Louis, MO, USA). The HL-60 line was a generous gift from Dr Orion D. Weiner (UCSF, San Francisco, CA, USA). All cells were maintained at 37°C with 5% CO_2_ in a Forma™ Steri-Cycle™ i160 CO2 Incubator (NC1207547, ThermoFisher Scientific). HL-60 cells were cultured in RPMI-1640 with 10% FBS, 25 mM HEPES, 1% sodium bicarbonate, and 1% sodium pyruvate. HEK293T and MEF cells were cultured in 10% FBS, in DMEM with sodium bicarbonate. HUVECs were cultured in endothelial cell growth medium. HL-60 cells were differentiated with 1.5% DMSO for 6 days. Cells were checked monthly for mycoplasma using the e-Myco plus Mycoplasma PCR detection kit. To generate knockdown lines in HL-60 cells, pLKO.1 lentiviral constructs with shRNA (*MFN2-sh1*:TRCN0000082684, *MFN2-sh2*: TRCN0000082687, *MFN1-sh*: TRCN0000051837, OPA1-sh: TRCN0000082846) were used, and SHC 003 was used as a non-targeting control. The MFN2 rescue construct was generated by replacing GFP in TRCN0000082684 with *sh1*-resistant *MFN2*. Primers MFN2r-F: 5′-CAAGTGTATTGTGAAGAGATGCGTGAAGAGCGGCAAG-3′ and MFN2r-R: 5′-TTCACAATACACTTGTTGCTCCCGAGCCGCCATG-3′ was used to make *sh1*-resistant *MFN2* with MFN2–YFP as the template. Primers: pLKO-F: 5′-AATTCTCGACCTCGAGACAAATGGC-3′ and pLKO-R: 5′-GGTGGCGACCGGGAGCGC-3′ were used to linearize the backbone of pLKO, and p-MFN2r-F: 5′-CTCCCGGTCGCCACCATGTCCCTGCTCTTCTCTCG-3′ and p-MFN2r-R: 5′-TCGAGGTCGAGAATTTTATCTGCTGGGCTGCAGGT-3′ were used to amplify *sh1*-resistant *MFN2* fragment. In-Fusion cloning was used to fuse the *sh1*-resistant *MFN2* fragment with the linearized backbone. The tether rescue construct was generated by replacing the GFP in TRCN0000082684 with a GFP containing a mitochondria localization signal (ATGGCAATCCAGTTGCGTTCGCTCTTCCCCTTGGCATTGCCCGGAATGCTGGCCCTCCTTGGCTGGTGGTGGTTTTTCTCTCGTAAAAAA) and ER localization signal (ATGGTTTATATTGGCATCGCTATTTTTTTGTTTTTGGTGGGCCTGTTTATGAAA) at it N- and C- terminal respectively. Primers used were: tether rescue+: 5′-CTCCCGGTCGCCACCATGGCAATCCAGTTGCGTTCG-3′, tether rescue−: 5′-TCGAGGTCGAGAATTTTAAGATACATTGATGAGTTTGG-3′. The Transomics shERWOOD UltramiR lentiviral inducible shRNA system (non-targeting control: TLNSU4300, mfn2 shRNA: ULTRA-3418270) was used for acute MFN2 deletion. Lentiviral constructs together with pCMV-dR8.2 dvpr and pCMV-VSV-G were co-transfected into HEK293T cells with Lipofectamin 3000 to produce lentivirus. Virus supernatant was collected at both 48 hpt and 72 hpt, and further concentrated with a Lenti-X concentrator. HL-60 cells were infected in complete medium supplemented with 4 µg ml^−1^ polybrene and selected with 1 µg ml^−1^ puromycin to generate stable lines.

### Microinjection

Microinjections of fish embryos were performed as described previously (Deng et al., 2011). Briefly, 1 nl of mixture containing 25 ng µl^−1^ plasmid and 35 ng µl^−1^ Tol2 transposase mRNA was injected into the cytoplasm of embryos at the one-cell stage.

### Tailfin wounding and Sudan Black staining

Tailfin wounding and Sudan Black staining were carried out with embryos at 3 days post fertilization (dpf) as described previously (Zhou et al., 2018b). Briefly, embryos were fixed in 4% paraformaldehyde in phosphate-buffered saline overnight at 4°C and stained with Sudan Black.

### Live imaging

Time-lapse images for zebrafish circulation, the LTB_4_ bath and the flow adhesion assay were obtained with an AXIO Zoom V16 microscope (Zeiss, Thornwood, NY, USA). Time-lapse fluorescence images for zebrafish neutrophil motility were acquired using a LSM 710 laser scanning confocal microscope (Zeiss, Thornwood, NY, USA) with a 1.0/20× objective lens at 1 min interval of 30 min. Neutrophils were tracked using ImageJ with the MTrackJ plugin and the velocity was plotted in Prism 6.0 (GraphPad, San Diego, CA, USA). Time-lapse fluorescence images for dHL-60 migration were acquired using a LSM 710 laser scanning confocal microscope (Zeiss, Thornwood, NY, USA) with a 1.0/20× objective lens at 10 s interval for 5 min. Cells were stained with 1 µM ER-tracker and 20 nM TMRM for 20 min, washed twice with Hanks' balanced salt solution (HBSS) and added to fibrinogen coated wells. After 30 min, cells were treated with 1 nM fMLP to induce chemokinesis.

### Confocal imaging

For confocal imaging, single slices of images were obtained using a LSM 800 laser-scanning confocal microscope (Zeiss, Thornwood, NY, USA) with a 1.4/63× oil immersion objective lens. Images were analyzed with ImageJ. For fluorescence intensity measurement, images within an experiment were acquired using identical camera settings and background was subtracted using ImageJ with the rolling ball radius of 50. The mean fluorescence intensity of selected areas was measured by use of the measurement tool in ImageJ and plotted in Prism 6.0. Colocalization was quantified using ImageJ Plugin Coloc 2. Interaction between channels was quantified by determining the Manders' colocalization coefficient as described previously (de Brito and Scorrano, 2008). Mitochondria clustering was defined as follows: a clustered morphology is defined as three or less distinct clusters in the cell; unclustered is defined as even distribution without major clusters; partial clustered is defined as scattered signal outside the three or less major clusters.

### µ-slide chemotaxis

dHL-60 cells were resuspended in mHBSS (modified HBSS with 20mM HEPES and 0.5% FBS) at 4×10^6^ ml^−1^ and loaded into µ-slides following the manufacturer's instructions. fMLP was added to the right-hand reservoir at a concentration of 1 µM. Chemotaxis was recorded every 1 min for 2 h using a laser scanning confocal microscope (LSM 710) with a 1.0/10× objective. The velocity of neutrophils was measured using ImageJ with the MTrackJ plugin and plotted in Prism 6.0. For inhibitor treatments, dHL-60 cells were pre-treated with DMSO, BAPTA (0.05, 0.1, 0.5, 1, 5 µM), NSC23766 (200 µM), CAS1090893 (50 µM), 2APB (50 µM) or RU360 (10 µM) for 30 min before loading into µ-slides. For 3D migration, dHL-60 cells were starved for 1 h in HBSS with 0.1% FBS and 20 mM HEPES. Chemotaxis was recorded every 1 min for 2 h, with a BioTek Lionheart FX Automated Microscope (Winooski, VT, USA) using a 10× phase objective, Plan Fluorite WD 10 NA 0.3 (1320516). Cells were tracked using MTrackJ image J plugin and plotted in Prism 6.0.

### Flow adhesion

The neutrophil flow adhesion assay was performed as described previously (Zhou et al., 2014). Briefly, 5×10^5^ HUVECs in 2 ml of medium were plated onto a 10 µg/ml fibrinogen-coated 35 mm plate (Corning 430165), and incubated at 37°C. Then the HUVEC monolayer was primed with 20 ng/ml human TNF-α for 4–6 h. Differentiated HL-60 cells were harvested and resuspended at a cell density of 5×10^5^ cells/ml in complete medium. Differentiated HL-60 cells were flowed on top of HUVEC monolayer at a speed of 350 µl/min using a syringe pump. Cells adhering to the monolayer were recorded using AXIO Zoom V16 microscope with camera streaming for 5 min. The total number of adherent neutrophils was quantified at 5 min.

### Rac-GTP pulldown assay

A Rac1 Pull-Down Activation Assay Biochem Kit was used to isolate active Rac from whole-cell lysate as described previously (Graziano et al., 2017). Briefly, dHL-60 cells were serum starved with RPMI medium lacking FBS for 1 h in the incubator at a density of 2×10^6^ cells ml^−1^. After starvation, cells were pelleted and suspended in mHBSS, and plated on a fibrinogen-coated 100 mm tissue culture dish to attach for 30 min. fMLP was then added to the cells at a final concentration of 100 nM, then cells were lysed with ice-cold lysis buffer at indicated time points and collected by scraping. 10 µg PAK–GST beads were mixed with each sample and incubated at 4°C for 1 h. Protein beads were washed and processed for western blotting.

### Western blotting

Protein samples were separated using SDS-PAGE and transferred onto nitrocellulose membranes in a Mini-PROTEAN Tetra vertical electrophoresis cell (Bio-Rad, Hercules, CA, USA). Membranes were blocked for ∼30 min in PBS and 0.1% Tween 20 (PBST) with 5% BSA. After blocking, membranes were incubated overnight with primary antibodies diluted 1:1000 in PBST at 4°C and secondary antibodies diluted 1:10,000 in PBST at room temperature for 1 h. A LI-COR Odyssey (BioAgilytix, Durham, NC, USA) device was used to image membranes. The phospho-PAK level was determined as described previously (Graziano et al., 2017). 1×10^6^–2×10^6^ dHL-60 cells were adhered to fibrinogen coated 6 cm dishes for 30 min, and stimulated with 100 nM fMLP for indicated time. Ice-cold stop solution (20% TCA, 40 mM NaF and 20 mM β-glycerophosphate) was immediately added to the cells at 1:1 volume and put on ice for 1 h. Lysates were pelleted (14,000 ***g*** for 10 min) and washed once with 0.75 ml of ice-cold 0.5% TCA and resuspended in 2× Laemmli sample buffer. Western blotting was performed as described previously (Hsu et al., 2019).

### Bone marrow neutrophil isolation

Femurs and tibias from mice of 6–8 weeks of age were isolated and bone marrow cells were collected and passed through a 70 µm filter followed by RBC lysis. Bone marrow neutrophils were isolated using the mouse neutrophil isolation kit. Neutrophils were stained with Trypan Blue and showed >99% viability.

### Peritonitis model

For the model of peritonitis, 1 ml of 4% thioglycollate was injected into the peritoneal cavity of mice of 6–8 weeks of age. After 3 h, 8 ml of PBS was injected into the cavity and the ascites were collected immediately. Cells were subjected to RBC lysis and viability was determined using Trypan Blue staining. Cells were stained with antibodies against CD11b and Ly6G on ice for 30 min and washed three times with staining buffer. Cells profiles were collected with an LSR Fortessa™ X-20 Cell Analyzer (BD Biosciences, Franklin Lakes, NJ, USA) device and analyzed with Kaluza software (Beckman Coulter Life Sciences, Indianapolis, IN, USA). The neutrophil population was defined as FCS/SSC high and CD11b^+^Ly6G^high^. The percentage of neutrophils in the lavage relative to total viable cells in each experiment was normalized to that from the sex-matched littermate control.

### Immunostaining

Differentiated HL-60 cells were resuspended in mHBSS and attached to fibrinogen-coated slides for 30 min. Cells were stimulated with 100 nM fMLP for 3 min and fixed with 3% paraformaldehyde in PBS for 15 min at 37°C. The immunostaining of fixed cells were performed as described previously (Fayngerts et al., 2017). Briefly, cells were permeabilized in PBS with 0.1% Triton X-100 and 3% BSA for 1 h at room temperature. Differentiated HL-60 cells were incubated with phalloidin–AlexaFluor 488 or primary antibodies diluted 1:100 in 3% BSA overnight at 4°C. The cells were then stained with secondary antibodies diluted 1:500 in 3% BSA and DAPI for 1 h at room temperature. For MEF staining, cells were plated on fibrinogen-coated slides and incubated for ∼4 h at 37°C, followed with fixation with 3% paraformaldehyde in PBS.

### Electron microscopy

Transmission electron microscopy was performed at the Purdue life science microscopy facility. dHL-60 cells were pelleted (14,000 ***g*** for 2 min) and fixed in 2.5% glutaraldehyde in 0.1 M sodium cacodylate buffer, post-fixed in buffered 1% osmium tetroxide containing 0.8% potassium ferricyanide, and en bloc stained in 1% aqueous uranyl acetate. They were then dehydrated with a graded series of acetonitrile and embedded in EMbed-812 resin. Thin sections (80 nm) were cut on a Leica EM UC6 ultramicrotome (Buffalo Grove, IL, USA) and stained with 2% uranyl acetate and lead citrate. Images were acquired using a Gatan US1000 2K CCD camera (Pleasanton, CA, USA) on a FEI Tecnai G2 20 electron microscope (Hillsboro, OR, USA) equipped with a LaB6 source and operating at 100 kV or 200 kV.

### Ca^2+^ measurement

Fluo-4 Ca^2+^ Imaging Kit was used for cytosolic Ca^2+^ measurement. Differentiated HL-60 cells were resuspended in mHBSS and incubated with PowerLoad solution and Fluo-4 dye at 37°C for 15 min and then at room temperature for 15 min. After incubation, cells were washed with mHBSS and loaded into fibrinogen-coated 96-well plates with 20,000 cells in 150 μl for each well, followed by incubation at 37°C for 30 min. Green fluorescence images were recorded with a BioTek Lionheart FX Automated Microscope (Winooski, VT, USA) with 20× phase lens at 1 s interval of 10 s. 15 µl of 1 µM fMLP was injected into cells using a reagent injector. Images were recorded for another 2 min with 1 s interval. The fluorescence intensity was normalized to that of the basal line level in each cell. For mitochondrial Ca^2+^ measurement, Rhod-2 was used. Differentiated HL-60 cells were incubated in mHBSS with Rhod-2 at 37°C for 30 min, and then washed and added into fibrinogen-coated 96-well plates with 150 µl well^−1^. After 30 min incubation, time-lapse red fluorescence images were acquired by the BioTek Lionheart FX Automated Microscope (Winooski, VT, USA) with 1 s interval of 10 s and followed by fMLP injection and imaged for another 2 min. The fluorescence intensity was normalized to that of the basal line in each cell. For the positive control, intracellular Ca^2+^ in dHL-60 cells was induced with 1 µM ionomycin or PBS.

### Cell spreading

The MEF cell spreading assay was performed as described previously (Jovic et al., 2007). Briefly, cells were trypsinized and replated onto fibrinogen-coated eight-well µ-slides with complete medium. Time-lapse images were acquired using BioTek Lionheart FX Automated Microscope (Winooski, VT, USA) with 20× phase lens at 2 min interval of ∼3 h at 37°C with 5% CO_2_.

### MEF morphology

MEF cells were seeded at 1×10^5^ cells ml^−1^ in 300 µl in chamber slides and stained with Mitotracker and WGA. Fluorescence images were acquired with a LSM 710 with a Plan-Apochromat 20×/0.8 M27 objective. Circularity was quantified using a custom algorithm (available at https://github.com/tomato990/cell_roundness_calculation).

### Wound closure

MEF cells in complete medium were seeded into 96-well plates and incubated at 37°C overnight. A wound was induced by a BioTek automated 96-well WoundScratcher (Winooski, VT, USA). Cells were washed twice with mHBSS and time-lapse images were acquired using BioTek Lionheart FX Automated Microscope (Winooski, VT, USA) with a 4× phase lens at 20 min interval of ∼12 h at 37°C with 5% CO_2_.

### Degranulation assay

1×10^6^ dHL-60 cells were stimulated with 100 nM fMLP for 60 min 37°C in suspension and washed with HBSS three times. Cells were then stained with anti-CD11b, CD18, CD63 and CD66 antibodies on ice for 30 min in 100 µl staining buffer (1% BSA, 0.1% NaN_3_ in PBS), which then were washed with staining buffer three times and resuspended in 300 µl of staining buffer and subjected to flow cytometry analysis.

### Flow cytometry analysis

Differentiated HL-60 cells were harvested and resuspended into ice-cold FACS buffer (PBS with 1% BSA) at a concentration of 1×10^6^ cells ml^−1^. Then, 5 ul of Annexin V solution was added into 100 μl cell suspension and incubated at room temperature for 30 min. Cells were washed for three times with ice-cold FACS buffer, and subjected to flow cytometry analysis. For surface markers, cells were incubated on ice for 1 h in staining buffer (1% BSA in PBS) containing CD18-PE, PE isotype control, CD11b–AF647, AF647 isotype control, CD15-BV510, BV510 isotype control or WGA–AF594, washed three times with staining buffer and resuspended in suitable volumes. Flow cytometry was performed using LSR Fortessa™ X-20 Cell Analyzer (BD Biosciences, Franklin Lakes, NJ, USA).

### NETosis assay

Differentiated HL-60 NETosis induction was performed as described previously (Hsu et al., 2019). Briefly, dHL-60 cells were resuspended in HBSS in 20 mM HEPES with 0.5% FBS and allowed to attach to fibrinogen-coated slides for 30 min at 37°C. Neutrophil extracellular traps (NETs) were induced with 50 nM PMA in HBSS for 4 h at 37°C. NETs were enumerated using cell permeable Hoechst 33258 at 1 µg ml^−1^ and cell impermeable Sytox Green at 1 µg ml^−1^. Images were acquired using LSM 710 with a Plan Apochromat 63×/1.4 Oil M27 or Plan-NEOFLUAR 10×/0.3 objective and processed with ImageJ. The percentage of cells forming NETs was calculated by dividing the number of Sytox Green-positive cells by that of the Hoechst-positive cells.

### Quantitative RT-PCR

Neutrophils from *Mfn2* conditional knockout mice were isolated and RNA was extracted using the RNeasy Mini Kit. HL-60 cells were differentiated for 6 days in 1.3% DMSO, and RNA was extracted using Qiagen RNeasy Mini Kit. mRNAs were reverse-transcribed with Transcriptor First Strand cDNA Synthesis Kit. Quantitative PCR were performed using the FastStart Essential DNA Green Master in a LightCycler^®^ 96 Real-Time PCR System (Roche Life Science, Brighton, MA, USA). Primers: mus-mfn2+, 5′-TCTTTCTGACTCCAGCCATGT-3′; mus-mfn2−, 5′-TGGAACAGAGGAGAAGTTTCTAGC-3′ (Luchsinger et al., 2016); mus-gapdh+, 5′-GGGTTCCTATAAATACGGACTGC-3′; mus-gapdh−, 5′-CCATTTTGTCTACGGGACGA-3′; hsa-mmp9+, 5′-GAACCAATCTCACCGACAGG-3′; hsa-mmp9−, 5′-GCCACCCGAGTGTAACCATA-3′; hsa-rpl32+, 5′-GAAGTTCCTGGTCCACAACG-3′; hsa-rpl32−, 5′-GAGCGATCTCGGCACAGTA-3′; hsa-OPA1+, 5′-GGTTGTTGTGGTTGGAGAT-3′; hsa-OPA1−, 5′-AGAGTCACCTTAACTGGAGAA-3′ (Amini et al., 2018). The specificity of the primers was verified as single peaks in the melting curves. The relative levels of mRNA were calculated using the ΔCt method. The relative fold change with correction of the primer efficiencies was calculated following instructions provided by the real-time PCR Miner (http://ewindup.info/miner/data_submit.htm; Zhao and Fernald, 2005).

### Mitochondrial membrane potential, ROS and ATP measurement

Differentiated HL-60 cells were resuspended in mHBSS and incubated with 150 nM MitoTracker, 20 nM TMRM, and 0.2 µg ml^−1^ Hoechst for 30 min at 37°C. Then cells were washed and plated onto fibrinogen-coated eight-well µ-slides. After 30 min incubation, cells were stimulated with or without fMLP at a concentration of 100 nM. The fluorescence images were acquired using a BioTek Lionheart FX Automated Microscope (Winooski, VT, USA) with a 20× phase lens, and processed using ImageJ. Mitochondrial membrane potential was measured using the fluorescence intensity of TMRM normalized to the intensity of MitoTracker of each cell. For mitochondrial ROS measurement, 5 µM of mitoROX was added to the cell suspension and incubated for 30 min at 37°C. Cellular ATP levels was measured by using the ATP Assay Kit. Briefly, dHL-60 cells treated with or without fMLP were harvested, washed with PBS, and resuspended in ATP Assay Buffer. Samples with ATP reaction mix were loaded into black wall 96-well plates and incubated at room temperature for 30 min protected from light. Results were measured using a BioTek microplate reader (Winooski, VT, USA) at excitation/emission=535/587 nm. All results were normalized to the values in control cell lines without fMLP treatment.

### Mutational efficiency quantification

The mutation efficiency of neutrophil-specific knockout in zebrafish was quantified as described previously (Zhou et al., 2018a). To determine the mutation efficiency in *Tg(lyzC:Cas9-mfn2 sgRNAs)^pu23^*, *Tg(lyzC:Cas9-mfn2 sgRNAs#2) ^pu24^*, and *Tg(lyzC:Cas9-opa1 sgRNAs) ^pu25^*, 3 dpf embryos of each line were digested with trypsin to prepare single-cell suspensions. mCherry-positive cells were sorted by FACS in Purdue Flow Cytometry and Cell Separation Facility using an Aria III Cell Sorter (BD Biosciences, Franklin Lakes, NJ, USA). Genomic DNA was purified using QIAamp DNA Mini Kit from sorted cells. 5 µg of poly(dA:dT) were used as the carrier DNA. The *mfn2 and opa1* loci around the sgRNA-binding sites were amplified using PCR with the following primers:

mfn2-F1: 5′-GGCGATGATAAACATGGCAGTTTG-3′, mfn2-R1: 5′-GTACCACAGGTGCACAGTGTC-3′, mfn2-F2: 5′-CTGGGACGCATCGGCCAATG-3′, mfn2-R2: 5′-CTACCTGCTTCAGGCATTCCCTG-3′, mfn2#2-F1: 5′-GTCGGGCTTCTCCTAAGTTATTC-3′, mfn2#2-R1: 5′-CAGTGTCCATAGCCTAGAGTCTGC-3′, mfn2#2-F2: 5′-GTGGTCTCATATAATTTTGCTTGCTG-3′, mfn2#2-R2: 5′-CACACGCGAATCGATAAGAGGAAT-3′, opa1-F1: 5′-CAAGCTCATTAAAGGTTTGAAACCACTTG-3′, opa1-R1: 5′-CTCCACAAATCACATAGGTGAC-3′, opa1-F2: 5′-GTGCCTGAATGCTCTACACTTTC-3′, opa1-R: CATGATAACAATACCATGCACATGC-3′. Purified PCR products were used for library construction with Nextera library prep kit and sequenced using an Illumina MiSeq 300 (San Diego, CA, USA) at the sequencing center of Purdue University. Mutational efficiency was calculated using the CrispRVariants R package (Lindsay et al., 2016).

### Statistical analysis

Statistical analysis was performed with Prism 6.0. A two-tailed Student's *t-*test or ANOVA was used to determine the statistical significance of differences between groups. A *P* value of less than 0.05, indicated in the figures by asterisks, was considered as statistically significant.

## Supplementary Material

Supplementary information

Reviewer comments
