# Supplementary material for: Mitofusin 2 regulates neutrophil adhesive migration and the actin cytoskeleton
Source: J Cell Sci. 2020 Sep 4;133(17):jcs248880. doi: 10.1242/jcs.248880 (PMC7491649; doi:10.1242/jcs.248880)
Supplement: Supplementary information [file joces-133-248880-s1.pdf]

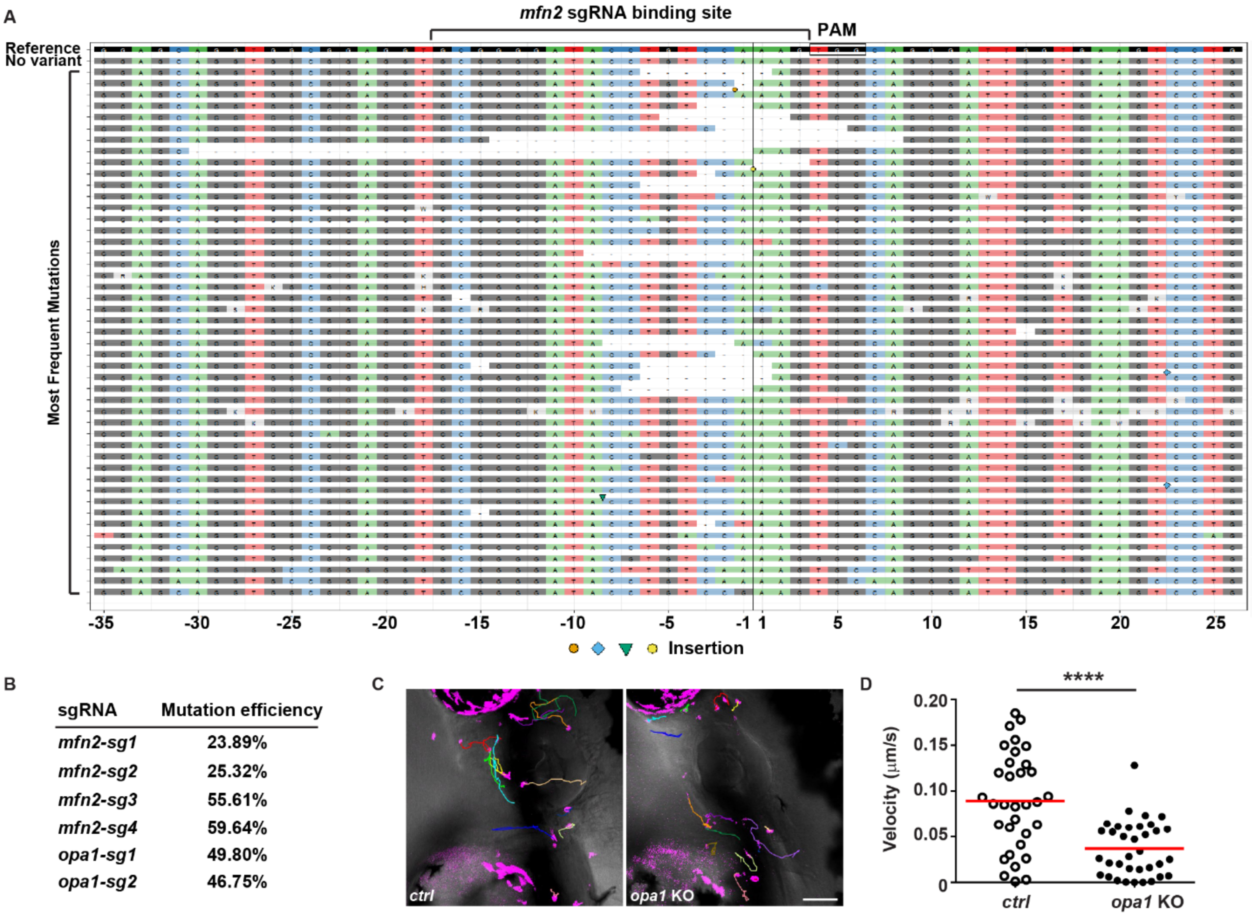

Figure S1. *In vivo* editing in the *mfn2 and opa1* loci. (A, B) Neutrophils were sorted via FACS from 3 dpf embryos of the transgenic lines. Genomic DNA was extracted and the *mfn2 or opa1* locus around the sgRNA cleavage site was PCR amplified and sequenced. (A) The 48 most frequent types of mutation at one of the *mfn2* locus are shown. Point mutations, deletions and insertions are all observed. Symbols indicate various forms of insertion. (B) Percentage of mutated alleles detected at each locus. Note, amplifying the genomic DNA from the sorted cells introduces PCR bias and the numbers are not a quantitative reflection of the degree of gene disruption. (C) Representative tracks and (D) quantification of neutrophil motility in the head mesenchyme of the control line and the line with neutrophil specific *opa1* knockout at 3 dpf. One representative result of three biological repeats is shown. n>20 cells were tracked. ****, p<0.0001, unpaired *t* test. Scale bar: 50 μm.

Journal of Cell Science • Supplementary information

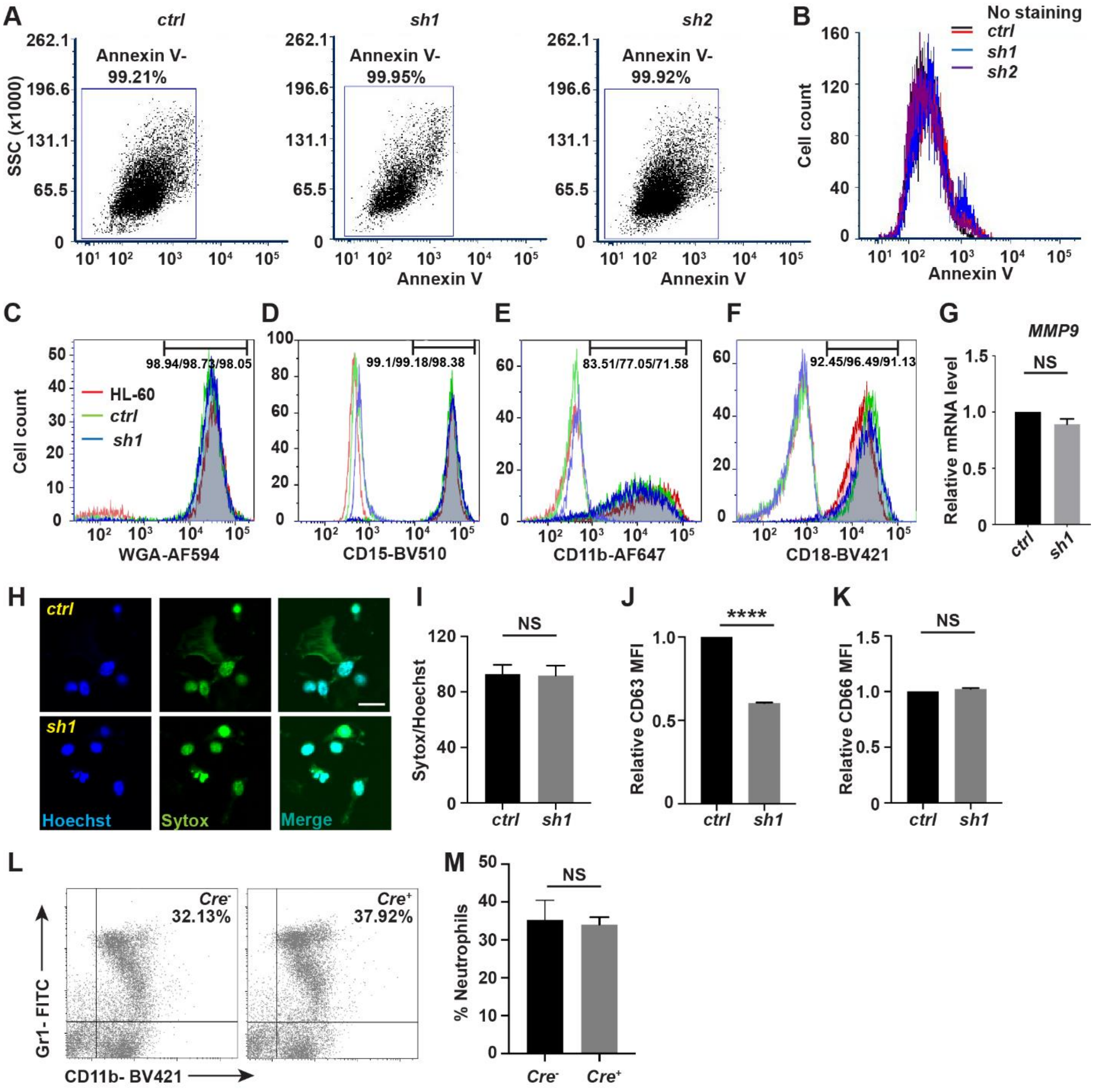

Figure S2. MFN2 deficiency does not induce apoptosis or affect cell differentiation of neutrophils. (A) Dot plot and (B) histogram of Annexin V staining. (C-F) Histogram of WGA, CD15, CD11b, and CD18 staining in indicated cell lines. (G) Relative mRNA level of *MMP9* in indicated cells lines. (H) Representative images and (I) quantification of neutrophil extracellular trap formation in *MFN2* knockdown or control dHL-60 cell. Percentage of cells with extracellular DNA is quantified. Scale bar: 50 µm. (J,K) fMLP-induced degranulation was

Journal of Cell Science • Supplementary information

analyzed by granule membrane markers CD63 for primary granules (J) and CD66 for secondary granules (K). MFI is calculated by geometric mean. The percentages of neutrophils in femoral bone marrow of *Mfn2$^{flox/flox}$; S100A8:Cre^{+}* or the control *Mfn2$^{flox/flox}$; S100A8:Cre^{-}* littermates at baseline. One representative result of three biological repeats is shown (A-F). Data are pooled from three independent experiments in G, I, J, K, M. NS, non-significant; ****, p<0.0001, unpaired *t*-test in G, I, J, K, M.

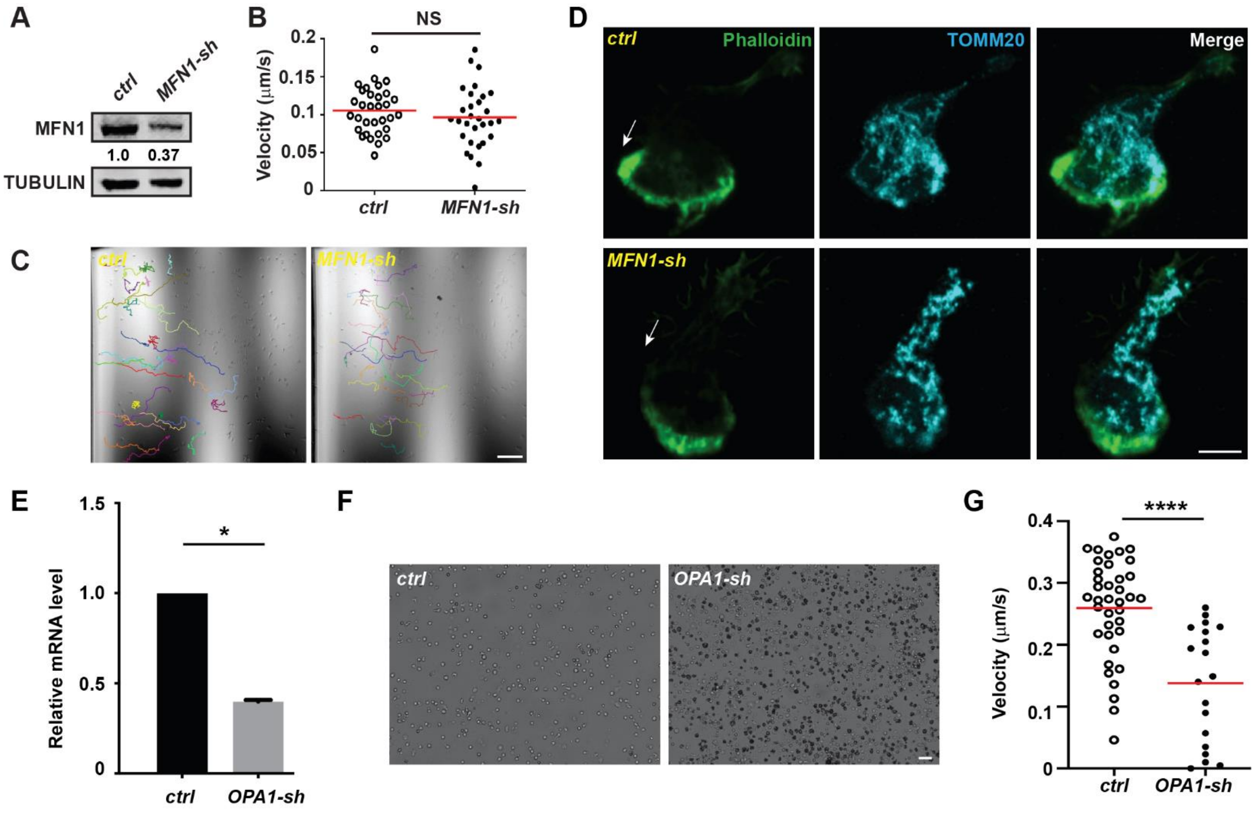

Figure S3. Effect of knocking down *MFN1* or *OPA1* on neutrophil chemotaxis. (A) Immuno blot showing the expression level of MFN1 in the control cell line or the one stably expressing a shRNA targeting *MFN1*. (B) Quantification and (C) representative tracks of indicated dHL-60 cells migrating toward fMLP. (D) Immunofluorescence of mitochondria (TOMM20) in indicated cells 3 min post fMLP stimulation. Cells were stained also with phalloidin to reveal F-actin. Arrows: direction of cell polarization. (E) Relative amount of OPA1 transcript in the control cell line or the one stably expressing a shRNA targeting *OPA1*. (F) Morphology of OPA1 deficient cells in culture. Massive cell death (nontransparent cells) are observed. (G) Quantification of indicated dHL-60 cells migrating toward fMLP. One representative result of three repeats is shown in B, F and G. Data are pooled from three independent experiments in E. n>20 cells were quantified and tracked in B and G. Scale bar: 100 µm in C and F, and 10 µm in D.

Journal of Cell Science • Supplementary information

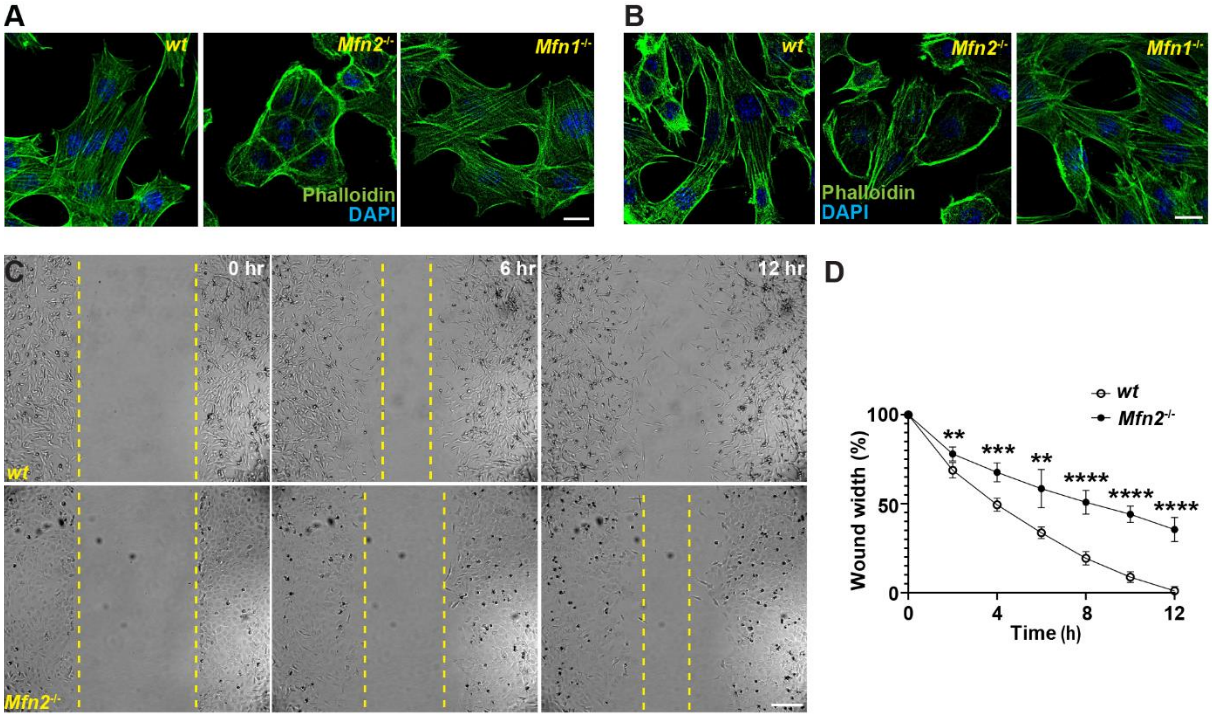

Figure S4. MFN2 regulates actin cytoskeleton and cell migration in MEFs. (A, B) Immunofluorescence of F-actin (Phalloidin) and nucleus (DAPI) in indicated cells plated on un-coated (A) or fibrinogen-coated (B) cover glasses. (C) Representative images of wt and *Mfn2-null* MEFs during wound closure at indicated time points. Yellow dash lines: wound edge. (D) Quantification of the wound area in wt and *Mfn2-null* MEFs during wound closure. One representative result of three biological repeats is shown. **, $p<0.01$, ***, $p<0.001$, ****, $p<0.0001$, unpaired *t*-test. Scale bar: 10 µm in A, B and 200 µm in C.

Journal of Cell Science • Supplementary information

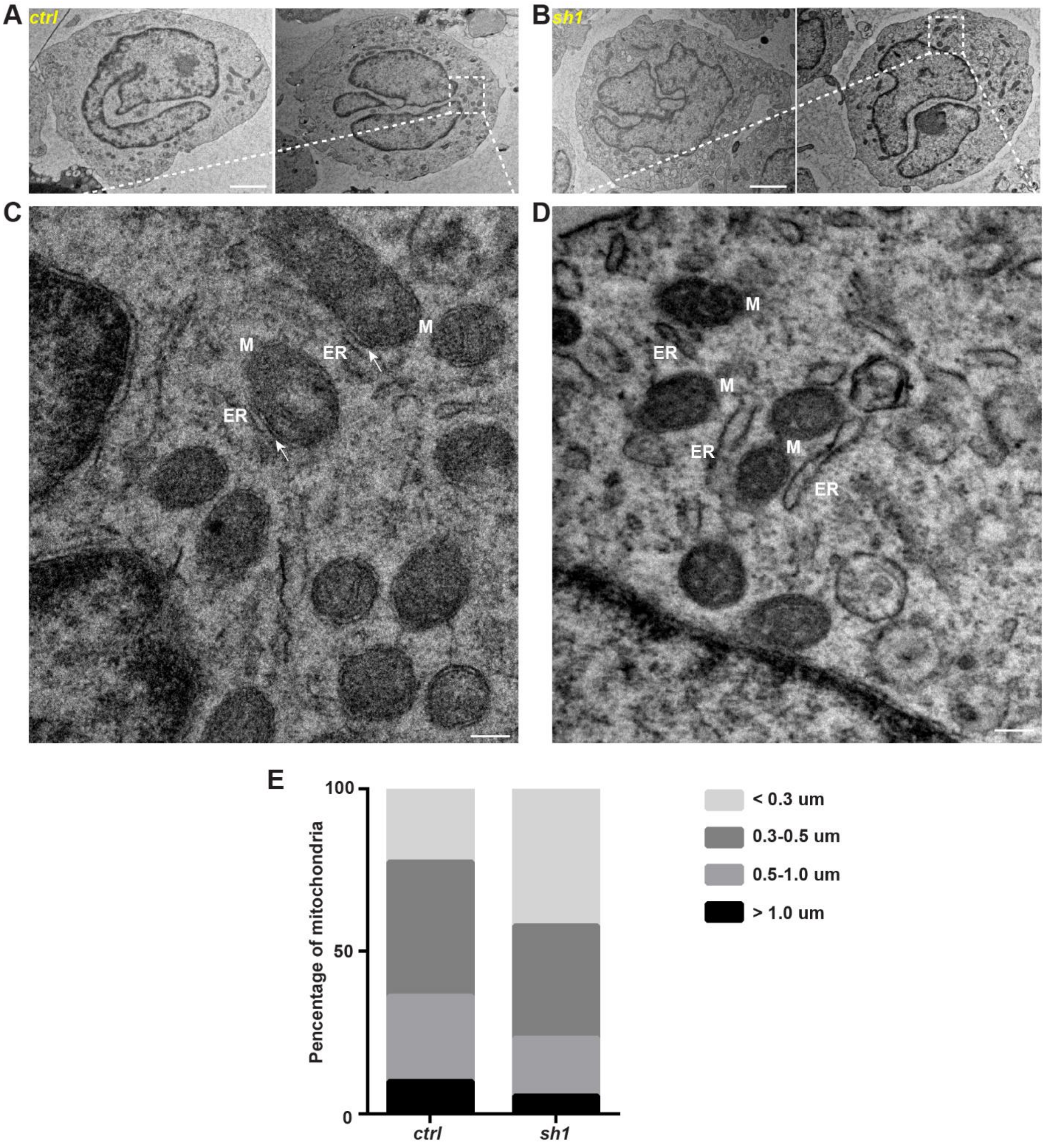

Figure S5. Electron microscopy of mitochondria and ER in MFN2 knock down dHL-60 cells. (A, B) Representative electron microscopy images of control and MFN2 knockdown dHL-60 cells. (C, D) Enlarged images of the white box in (A) and (B). M: mitochondria, ER: endoplasmic reticulum. White arrows: contact sites between mitochondria and ER. (E) Quantification of the length of mitochondria in control and *MFN2-sh1* dHL-60 cells. n>20 cells were quantified in E. Scale bar: 2 μm in A and B; 0.2 μm in C and D.

Journal of Cell Science • Supplementary information

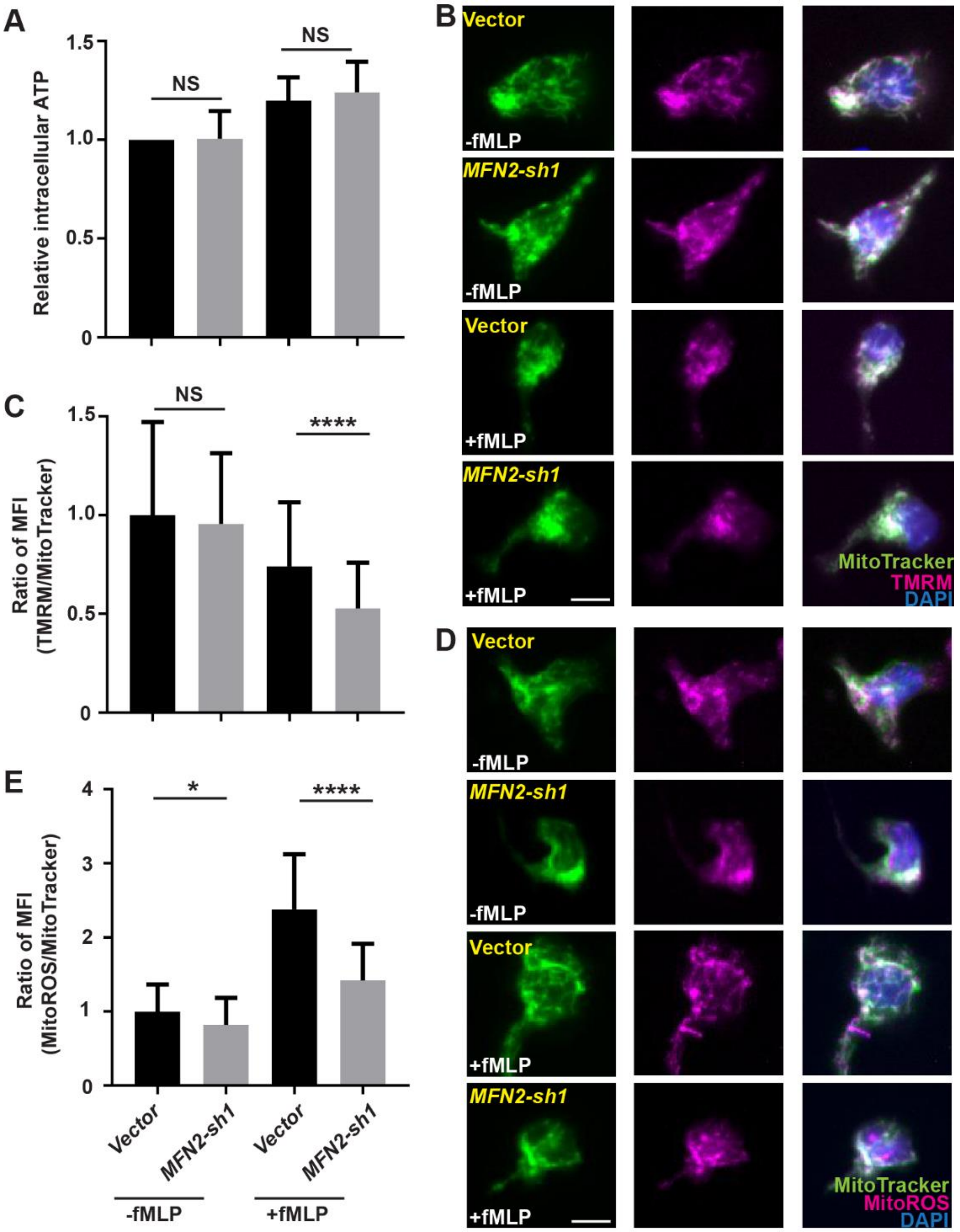

Figure S6. MFN2 regulates mitochondrial membrane potential and ROS, but not ATP in dHL-60 cells. (A) Quantification of cellular ATP levels with or without treatment of fMLP for 3 min. (B) Fluorescence of mitochondria (MitoTracker), membrane potential (TMRM), and nucleus (Hoechst) in the control or *MFN2* knockdown cell lines with or without treatment of fMLP for 3 min. (C) Quantification of the mean fluorescence intensity of TMRM normalized

Journal of Cell Science • Supplementary information

with MitoTracker. (D) Fluorescence of mitochondria (MitoTracker), mitochondrial ROS (MitoROS), and nucleus (Hoechst) in indicated cells with or without treatment of fMLP for 3 min. (E) Quantification of mean fluorescence intensity of MitoROS normalized with MitoTracker. Data are pooled from three independent experiments (A, C, E) *, $p<0.05$; ****, $p<0.0001$, NS, no significance, unpaired *t*-test. Scale bar: 10 μm in B and D.

Journal of Cell Science • Supplementary information

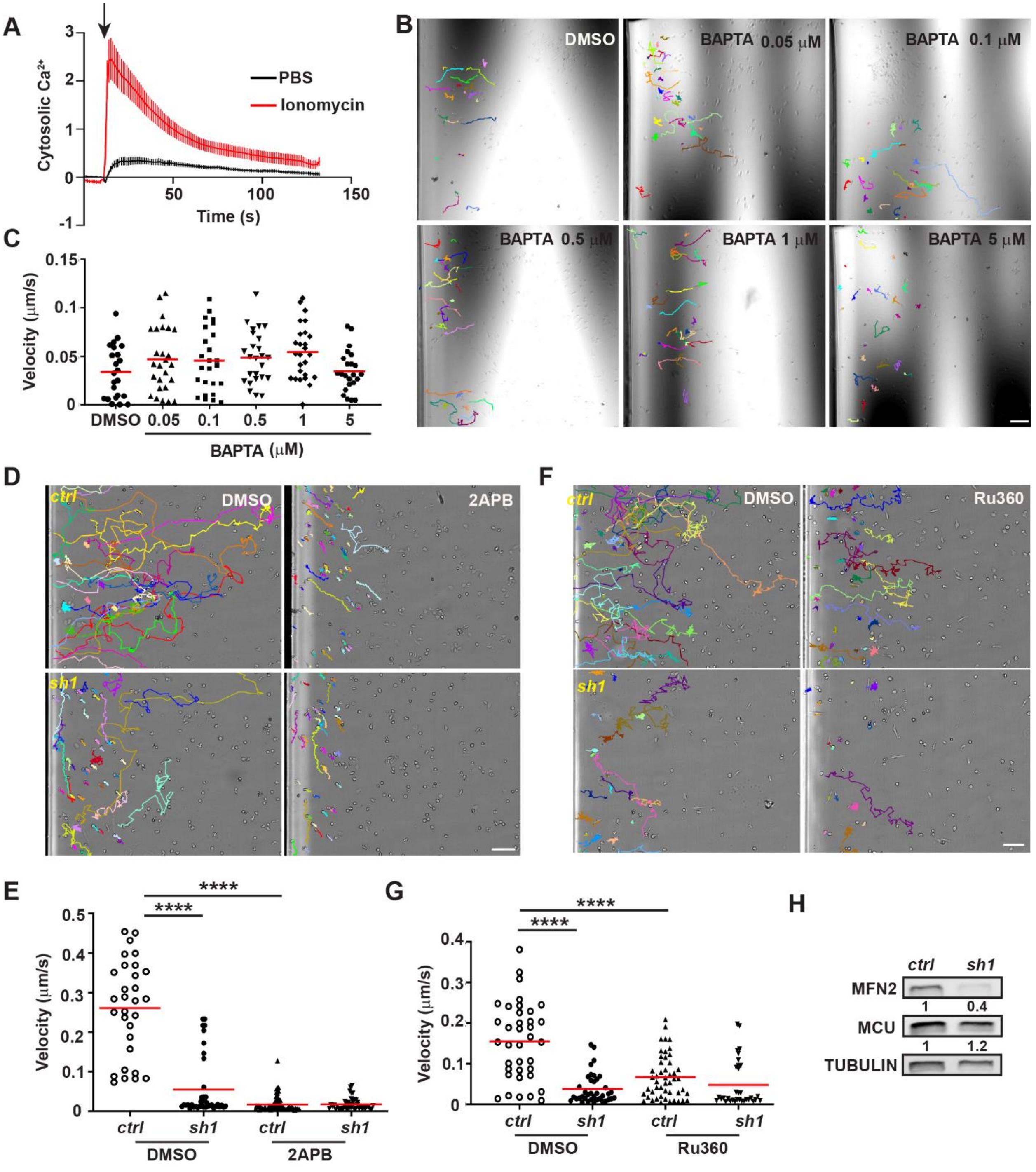

Figure S7. Buffering intracellular Ca^{2+} does not rescue the migration defect of *MFN2* knockdown dHL-60 cells. (A) Cytosolic Ca^{2+} in dHL-60 cells after stimulated with ionomycin or PBS. (B) Representative tracks and (C) quantification of MFN2 knockdown dHL-60 cells migrating toward fMLP in the presence of indicated concentrations of BAPTA. (D)

Journal of Cell Science • Supplementary information

Representative tracks and (E) quantification of neutrophil velocity migrating to fMLP in the presence of DMSO or IP3R inhibitor, 2APB. (F) Representative tracks and (G) quantification of neutrophil velocity migrating to fMLP in the presence of DMSO or MCU inhibitor, Ru360. (H) Immunoblot showing the expression level of MNF2 and MCU in the control or *MFN2* knock down cell line. One representative result of three repeats is shown in A-H. n>20 cells were quantified and tracked in B-G. ****, p<0.0001, by unpaired *t*-test in E and G. Scale bar: 100 μm in B, D and F.

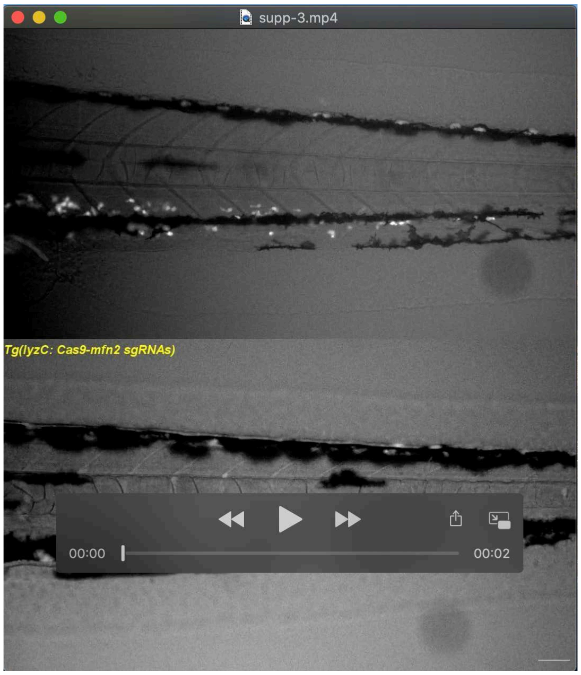

Movie 1. Neutrophil specific deletion of *mfn2* alters neutrophil homeostasis in zebrafish.

Time-lapse images of neutrophil homeostasis in 3 dpf embryos from the transgenic lines with neutrophil specific *mfn2* knockout and the control. Scale bar: 50 µm.

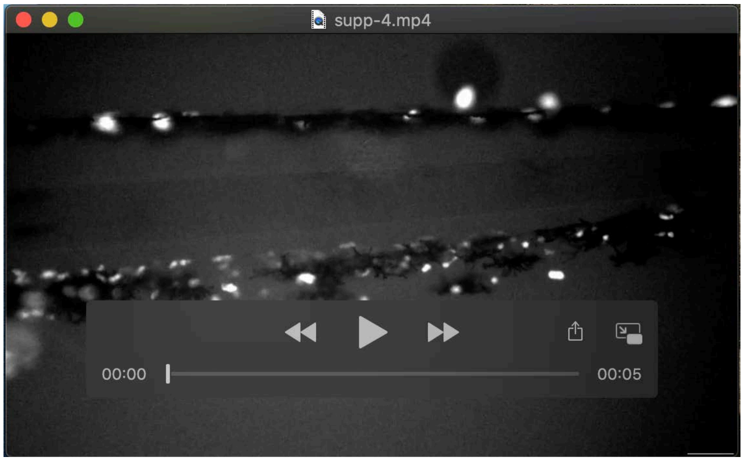

Movie 2. Neutrophil specific deletion of *mfn2* using a second set of sgRNAs results in a similar phenotype.

Time-lapse images of neutrophil homeostasis in 3 dpf embryos from a second transgenic line expressing different sgRNAs for neutrophil specific *mfn2* knockout. Scale bar: 50 µm.

Journal of Cell Science • Supplementary information

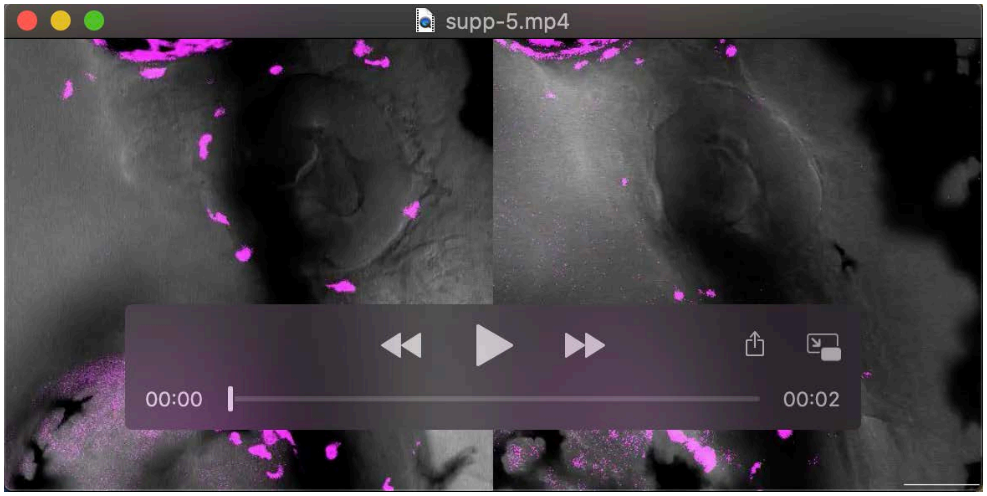

Movie 3. Opa1 is required for neutrophil motility in zebrafish.

Time-lapse images of neutrophil migration at the head mesenchyme and individual neutrophils were tracked for velocity quantification. Scale bar: 50 µm.

Journal of Cell Science • Supplementary information

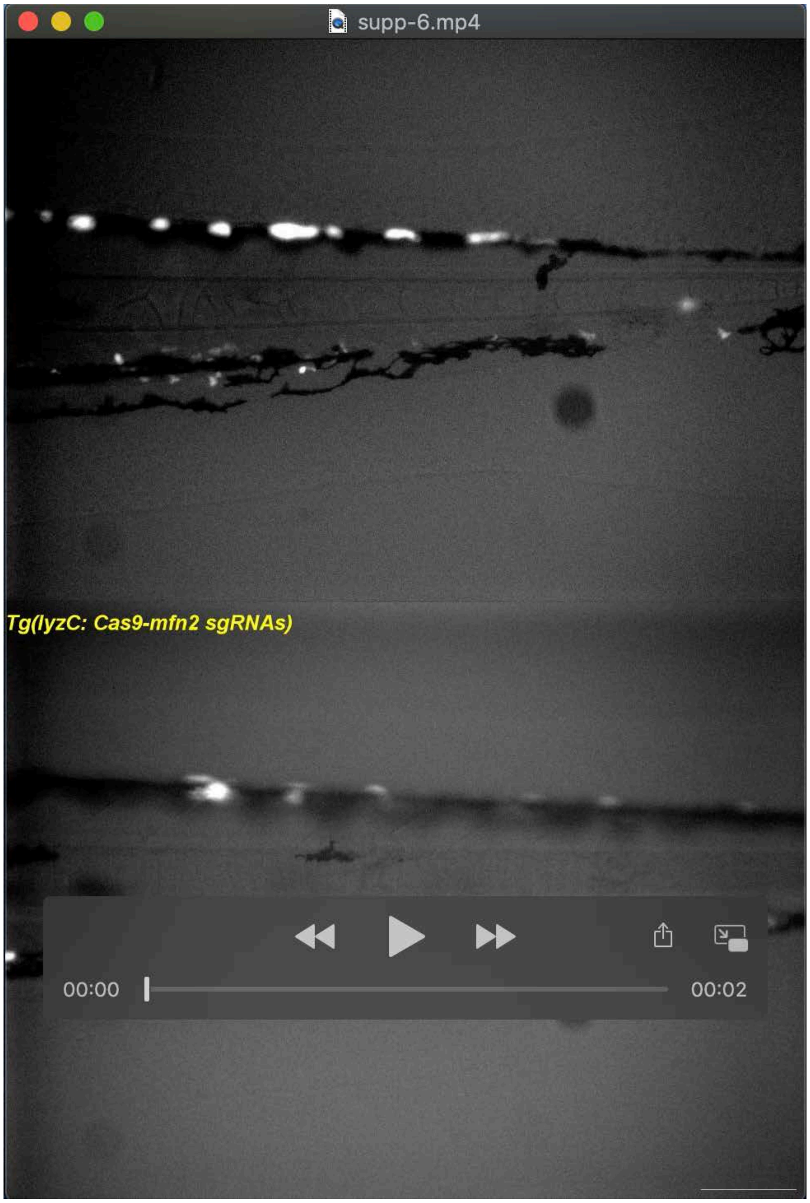

Movie 4. Mfn2 is required for neutrophil chemotaxis to LTB_4 in zebrafish.

Time-lapse images of neutrophil chemotaxis in 3 dpf embryos from the transgenic lines with neutrophil specific *mfn2* knockout and the control after LTB4 treatment. Scale bar: 50 µm.

Journal of Cell Science • Supplementary information

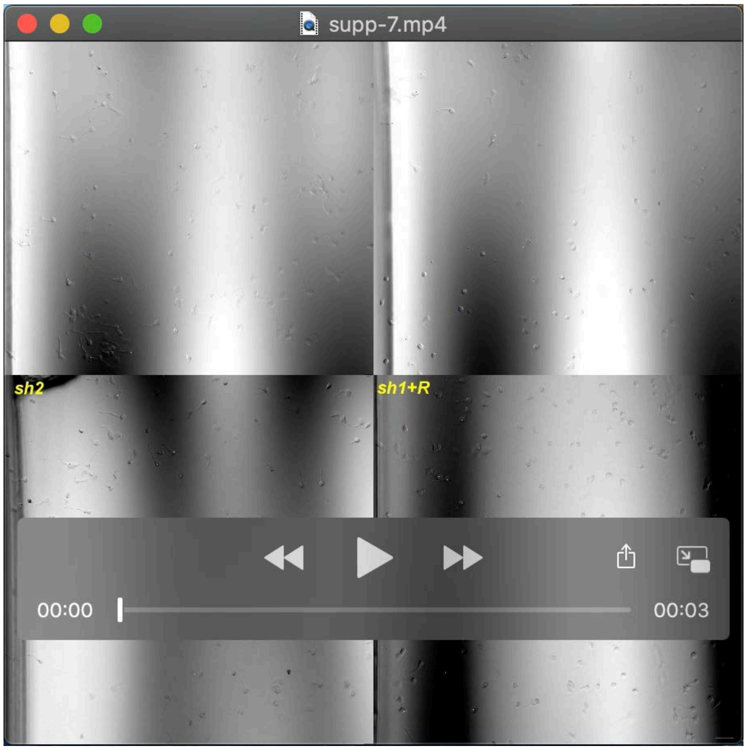

Movie 5. MFN2 regulates adhesive migration in dHL-60 cells.

Chemotaxis of the control, *MFN2* knockdown, and *MFN2* rescue cells towards fMLP in the μ-slide. Individual neutrophils in the left half of the chamber were tracked. Scale bar: 50 μm.

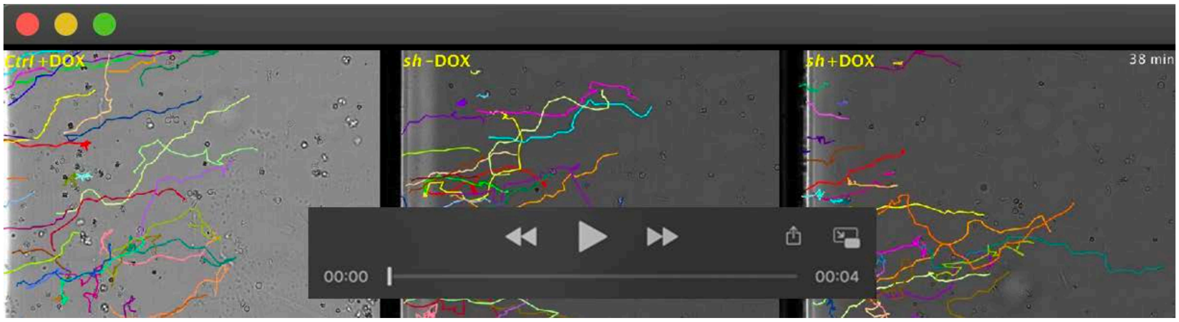

Movie 6. Acute deletion of MFN2 reduced adhesive migration in dHL-60 cells.

Chemotaxis of the control with dox, *MFN2*-sh1 with or without dox cells towards fMLP in the μ-slide. Individual neutrophils in the left half of the chamber were tracked. Dox: doxycycline. Scale bar: 50 μm.

Journal of Cell Science • Supplementary information

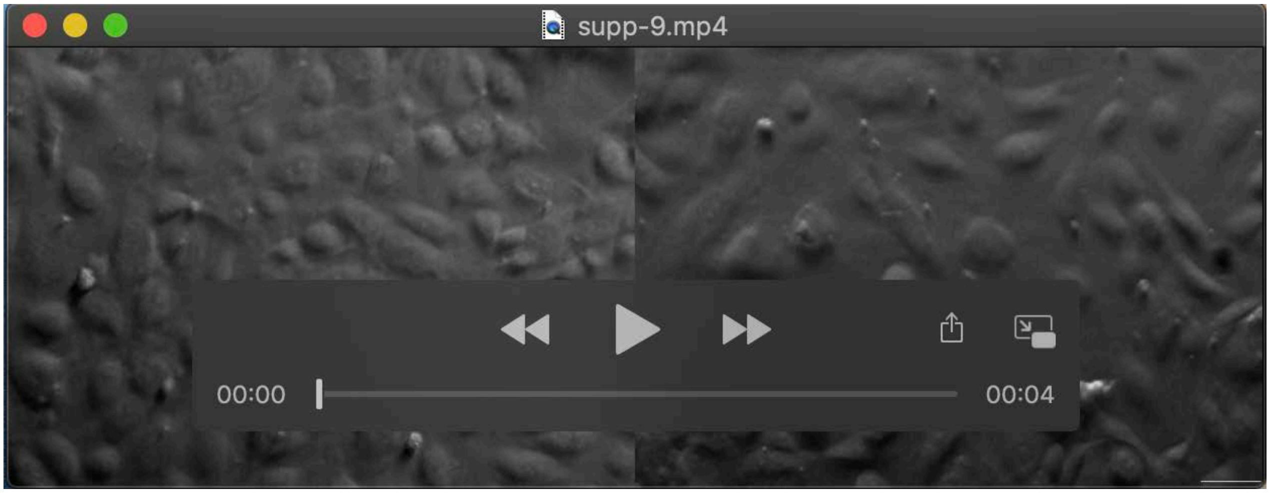

Movie 7. *MFN2*-deficient dHL-60 cells have defective adhesion to endothelial cells.

HUVEC cell monolayer was stimulated by 20 ng ml^{-1} TNF-α for 4-6 h. Vector control and*MFN2-sh1* dHL-60 cells were flowed through HUVEC layers with a speed of 350 µl min^{-1} and recorded by microscope with no interval. Scale bar: 50 µm.

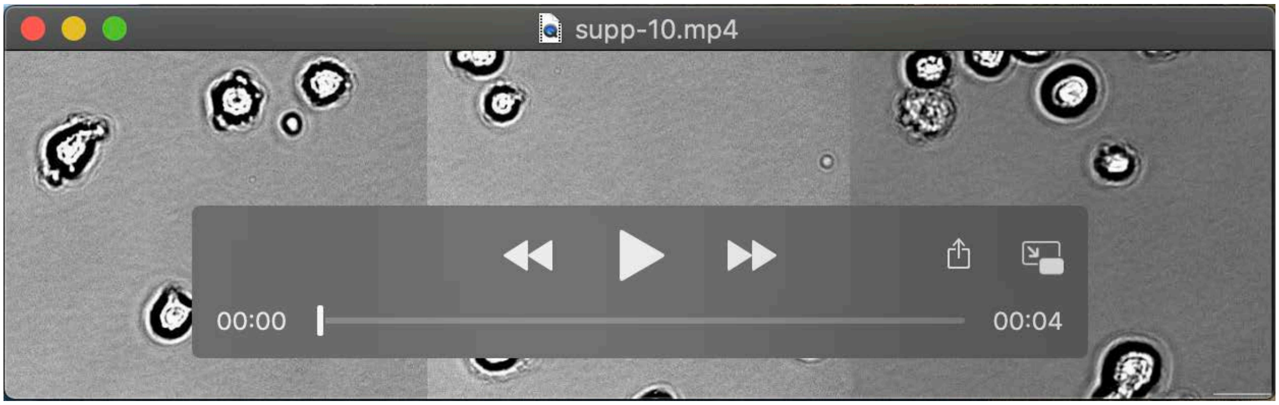

Movie 8. MFN2 regulates spreading of MEF cells.

Spreading of the control, *Mfn2*$^{-/-}$ and *Mfn1*$^{-/-}$ cells on 8 well µ-slides immediately after plating. Scale bar: 20 µm.

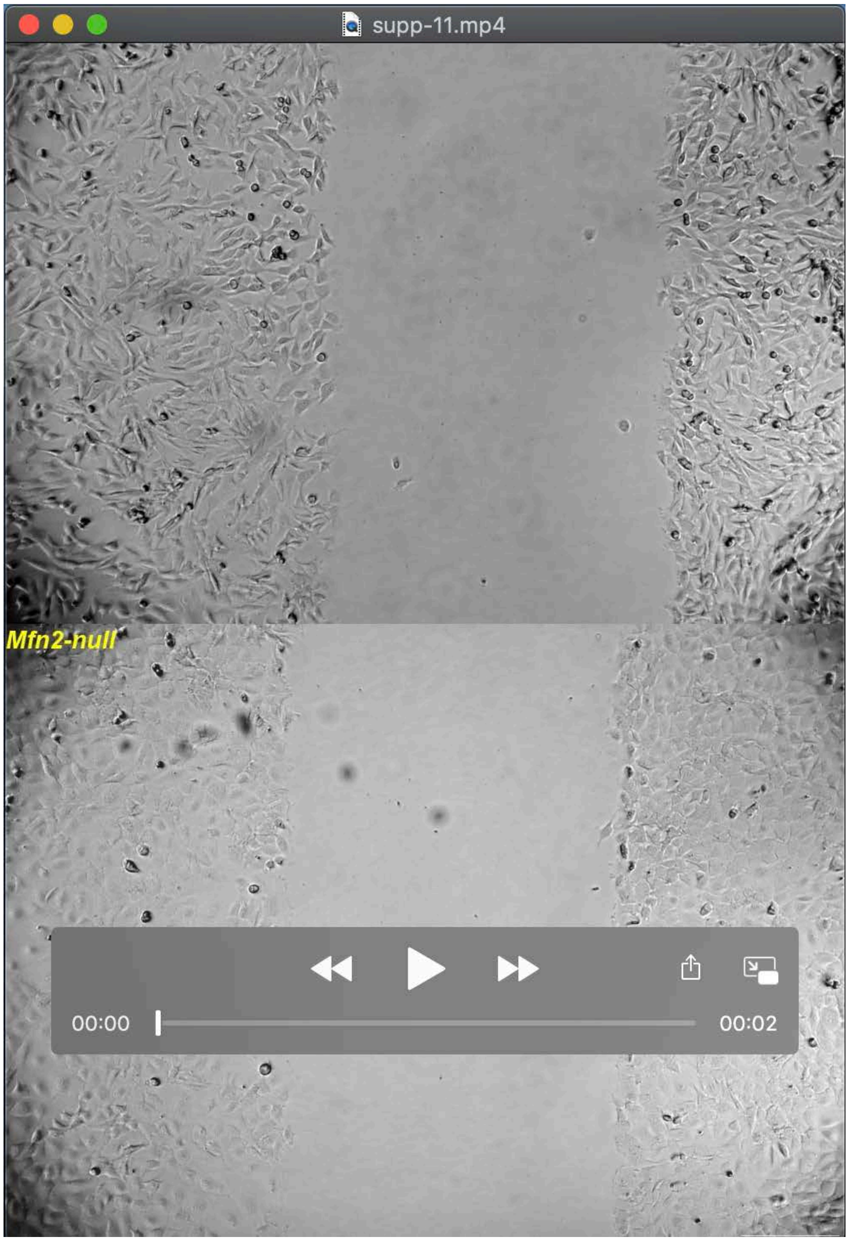

Movie 9. MFN2 regulates wound closure of MEF cells. Wound closure of the control and *Mfn2*$^{-/-}$ MEFs. Scale bar: 200 µm.

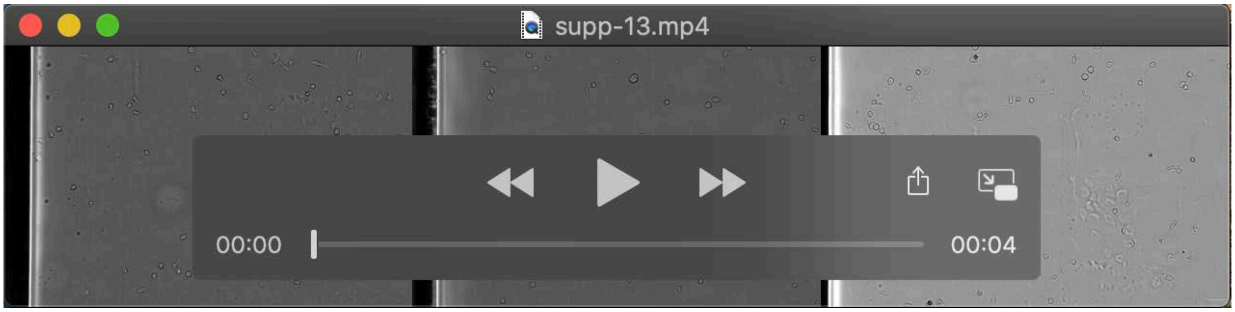

Movie 10. Restoring ER-mitochondrial tether rescue the migration defects in *MFN2*-deficient dHL-60 cells.

Chemotaxis of control, *MFN2*-deficient, and *MFN2*-deficient cells with synthetic tether construct towards fMLP in the µ-slide. Individual neutrophils in the left half of the chamber were tracked. Scale bar: 50 µm.

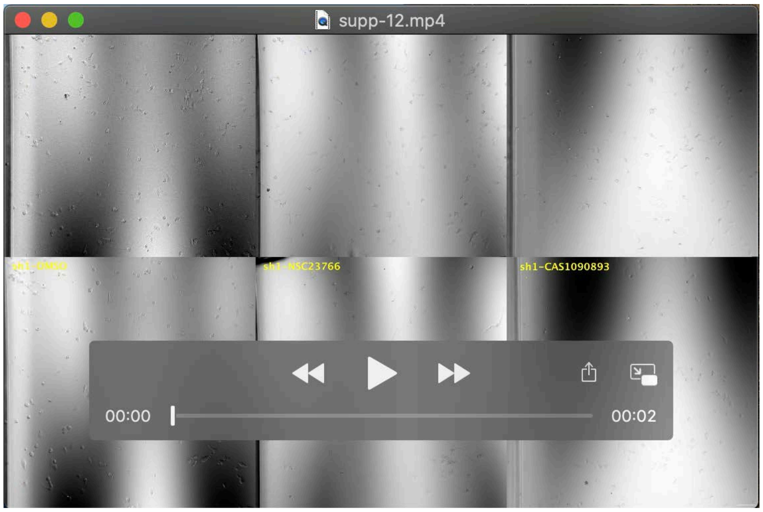

Movie 11. Rac inhibition restores chemotaxis in *MFN2*-deficient dHL-60 cells.

Chemotaxis of control and *MFN2* knockdown cells treated with DMSO, NSC23766 (200 µM) or CAS 1090893 (50 µM) towards fMLP in the µ-slide. Individual neutrophils in the left half of the chamber were tracked. Scale bar: 50 µm.
