# Supplementary material for: Mitofusin 2 regulates neutrophil adhesive migration and the actin cytoskeleton
Source: J Cell Sci. 2020 Sep 4;133(17):jcs248880. doi: 10.1242/jcs.248880 (PMC7491649; doi:10.1242/jcs.248880)
Supplement: Reviewer comments [file JCS248880_review_history.pdf]

Mitofusin 2 regulates neutrophil adhesive migration and the actin cytoskeleton

Wenqing Zhou, Alan Y. Hsu, Yueyang Wang, Ramizah Syahirah, Tianqi Wang, Jacob Jeffries, Xu Wang, Haroon Mohammad, Mohamed N. Seleem, David Umulis and Qing Deng
DOI: 10.1242/jcs.248880

Editor: Daniel Billadeau

Review timeline

Submission to Review Commons:	24 February 2020
Submission to Journal of Cell Science:	12 May 2020
Editorial decision:	15 May 2020
First revision received:	24 July 2020
Accepted:	29 July 2020

Reviewer 1

Evidence, reproducibility and clarity

The manuscript by Zhou et al. describes the role of mitofusin-2 in neutrophil adhesive reproducibility migration. The authors suggest that MFN-2 is required to sustain neutrophil migration and and clarity link this observation to the role of MFN-2 in maintaining mitochondria-ER contacts and (Required)suppressing Rac activation.
Although some of the experiments are convincing, the authors come to conclusions that are not entirely supported by their data and a few statements appear the result of inductive reasoning.
A major problem is the distinction between adhesion and migration: in several parts of the manuscript, there is confusion between these two events and the experiments are not designed (and not discussed) in order to clarify this point.
For example, the fact that in zebrafish embryos lacking Opa1 there is no defect in neutrophil retention but reduced neutrophil migration should suggest that MFN-2 controls adhesion rather than migration. But this is not properly elaborated. The same problem comes with the role of Rac, which has been elegantly shown to be required for cell migration but not for cell spreading or focal adhesion formation (Steffen et al, JCS 2013). Again, it is necessary to distinguish between migration and other functions requiring the actin cytoskeleton.

Specific comments:

Introduction:
"Although mitochondria-derived ATP possibly regulates neutrophil chemotaxis in vitro (Bao et al., 2015), removal of extracellular ATP improves neutrophil chemotaxis in vivo (Li et al., 2016). These conflicting reports prompted us to search for mechanisms delineating the role of mitochondria in neutrophil migration outside the realm of ATP or cellular energy (Bi et al., 2014; Schuler et al., 2017; Zanotelli et al., 2018)."
This sentence is superficial and misleading: extracellular ATP may interfere with chemotaxis through various energy-independent mechanisms (see for example Zumerle et al. Cell Reports 2019) and this is not conflicting with the role of intracellular ATP in migration.

Figure 1:
The authors didn't show evidence of the genome edition (PCR, RFLP or Sequencing over the sgRNA target) or at least RT-PCR or WB for MFN2.
In Fig 1b, 1c the scale bar is missing.

© 2020. Published by The Company of Biologists under the terms of the Creative Commons Attribution License (https://creativecommons.org/licenses/by/4.0/).

"Neutrophils were sorted from both lines and their respective loci targeted by the 4 sgRNAs were deep sequenced." There are no data about sorting strategies for zebrafish neutrophils in the figure. Moreover, only 2 sgRNAs are shown and there are no sequencing data.

Figure 2:
In the WB, reconstitution is not obvious. In general, all WBs are not quantified (and they should be quantified).
The in vivo experiment does not have proper controls. For example, can the authors exclude that in these mice there is reduced inflammation because neutrophils have defective activation? What about NETs? And cytokines/chemokines? And exocytosis? In the absence of these controls, the experiment cannot be properly interpreted.

Figure 3:
The conclusion of the authors "In summary, Mfn2 modulates the actin cytoskeleton and cell migration in MEFs" should be supported by experiments to distinguish between the specific role of Mfn2 and the role of mitochondrial dynamics (Opa1, Drp1, Mfn1). It is also not clear why the authors decided to use MEFs instead of other cells (more similar to neutrophils which are not adherent cells). The results obtained in MEFs may be irrelevant for neutrophils.

Figure 4-5:
Fig 5a: in ctrl and sh1 the ER seems to be larger than the phalloidin (=cytoskeleton=cell border approximately) in a few regions. Only the sh1+T seems to fit correctly.
The TEM image (only 1 in supplementary) is not sufficient to convince that the tethering is lost. Quantification of number of contacts and distance between ER and mitochondria should be included.
The title of figure 5 is wrong.
However, in these figures, it is clear that cells are beautifully polarized, with mitochondria accumulating at the uropod (and even more in the absence of Mfn2). When comparing these images with those published by Campello et al (JEM 2006), there are 2 observations that can be made: first of all, these data confirm that mitochondrial fission promotes cell polarity; second, they suggest that the defect is not at the level of cell polarity/chemotaxis.

Figure 6:
Calcium data are, in general, very weak. First of all, controls with ionomycin are missing. Statistical analyses of the curves should be included. As for the use of the MCU inhibitor Ru360, is there any evidence that it is cell-permeant in this context? Is it blocking MCU? Since the authors can show mitochondrial calcium upon FMLP, they should also demonstrate that Ru360 is indeed working and inhibiting mitochondrial calcium uptake. The sentence "The MCU inhibitor Ru360did not cause further reduction of chemotaxis in MFN2 knockdown dHL-60 cells (Supplementary Fig. 6c, d and Supplementary Movie. 12),indicating that MCU and MFN2 lies in the same pathway in terms of regulating chemotaxis in dHL-60 cells" is speculative.
In general, there is no solid demonstration that the effect is calcium-mediated.
As for Rac, it is surprising to see that Rac inhibition has no effect on cell migration. Rac is known to promotes migration in fibroblasts and other cell types and Rac deficiency inhibits migration (see for example Steffen et al, JCS 2013). Two sets of experiments are absolutely required: 1) verify this in fibroblasts since it has been elegantly shown that Rac is essential in these cells for migration; 2) analyse the effect of Rac inhibitors in pPak kinetics.

Significance

As presented here, the manuscript has a modest significance. The audience would be specialised: cell migration, cell signalling.
My expertise is immunology, cell activation, cell migration, cell signalling.

Reviewer 2

Evidence, reproducibility and clarity

The manuscript by Zhou W and colleagues entitled "Mitofusin 2 regulated neurophil adhesive migration and actin cytoskeleton" proposed that mitochondrial outer membrane GTPase Mitofusin 2

© 2020. Published by The Company of Biologists under the terms of the Creative Commons Attribution License (https://creativecommons.org/licenses/by/4.0/).

controls cell migration via its capacity to regulate mitochondria- endoplasmic reticulum (ER) contacts, independently of its fusogenic activity. Using transgenic Mfn2 zebrafish, they first show that Mfn2 mutant embryos exhibit circulating neutrophils and defects in neutrophil recruitment to generated wound, compared to control. Then, using a combination of in cellulo and in vivo mouse models, they show that loss of Mfn2 decreases neutrophil migration, their adhesion under sheer stress and their infiltration to the peritoneal cavity in vivo. Third, they confirm these results using Mfn2 KO MEFs, where they show migration and actin skeleton defects, in contrast to Mfn1 KO MEFs. Mechanistically, they propose that migration defects induced by Mfn2 loss are associated to a decrease of membrane contact sites between the ER and the mitochondria. Using different in cellulo cell migration assays, they show that migration defects in Mfn2-null was rescued upon an artificial mito-ER tether. Finally, they propose that the loss of Mfn2 leads to cytosolic calcium accumulation, inducing hyper-activation of the RhoGTPase, Rac1, a key regulator of actin dynamics and cell migration. Together, the authors proposed a new function of Mfn2 in regulating cell migration via mito-ER contacts tethering.

Major comments:

Although the results could be very interesting, and could be significantly relevant to the mitochondrial field and the cell biology one in general, major points need to be addressed to fully support conclusions of the authors. Different controls and quantification are missing, Actin dynamics analysis should be improved, effects of the artificial tether is weakly characterized and the demonstration of the specific role of mito-ER contacts via mfn2 in migration should be reinforced.

-In figure 1, quantification of circulating neutrophils is required in Mfn2 KO embryos. The authors should also show these quantified results for OPA1KO, which are just mentioned in the text. In addition, in figure 1b and d, the neutrophils from the Mfn2KO embryos seem bigger compared to control. Can the authors comment on neutrophils size and potential contribution to the phenotype? Finally, the authors propose a defect in neutrophil migration in Mfn2-KO, however neutrophils are found in the circulation. The authors should explain these results.

-The authors need to reinforce the Mfn2 specificity for their phenotype. In particular in Fig S1, they show that loss of OPA1 significantly decreases neutrophil migration in vivo.
However, they then only study the effect of Mfn1 silencing in neutrophil and MFN1 KO MEFs (Sup Fig s3). The authors should perform the same experiments in neutrophil and MEF upon loss of OPA1 (similar to Fig S3). Does loss of OPA1 and Mfn1 decrease neutrophil arrest to activated endothelial cells?

-Using their images, the authors should also document on the directionality of the cell during cell migration. Do Mfn2 depleted cells do not migrate because they are arrested or because they are lacking directionality? Environment/chemokine sensing defects?

-Actin dynamics analysis should be improved. Loss of Mfn1 and Mfn2 lead to cell shape changes. The authors should quantify this phenotype by analysing cell circularity (as well as for Opa1 loss). Stress fibres number or Phalloidin intensity quantification in cell body should also be performed.

-Can the migration defects could be attributed to Focal adhesion protein dynamics defects? The authors shown an hyperactivation of Rac1 and an hyperphosphorylation of PAK, which can control FAP (focal adhesion proteins) dynamics. In addition, immunofluorescence analysis shows a decreased signal and cellular misdistribution of paxillin. The authors should characterize these phenotypes. FAP levels (Paxillin/Phospho-Paxillin and Vinculin) should be analysed by immunoblot, the number of FAP/cell, distribution and size should also be quantified. Their dynamics should also be analysed by live cell imaging. Finally, Paxillin level and distribution seems to be also impacted in Mfn1KO cells. Can the authors comment on that? The different quantifications would help to better understand the effect of different mitofusins in cytoskeleton dynamic.

-Please perform rescue experiments for cell migration in MFN2KO and MFN1KO MEFs. Immunoblots showing protein levels of these proteins would be appreciated. To really discriminate how Mfn2 regulates cell migration, the authors should also perform rescue experiments using a fusogenic

© 2020. Published by The Company of Biologists under the terms of the Creative Commons Attribution License (https://creativecommons.org/licenses/by/4.0/).

mutant Mfn2 ((K109A). It will help to demonstrate the relevance of mito-ER contacts and not mitochondrial fusion in the phenotype.

-Figure 4, the authors stipulate that Mfn2 regulates ER-mitochondria tethering. However, the authors present no evidence for this conclusion. The authors should perform manders coefficient in MFN2 KO cells and compared it to control. Also, loss of Mfn2 induces mitochondrial fragmentation, which can lead to problem for mito-er contacts quantification by light microscopy. The authors should use their TEM pictures to quantify mito-ER contacts (Number, length and % of mito perimeter), not only mitochondrial morphology. Mfn1 should be used as negative control. it would be interesting also to determine the status of the mito- ER contact in the different conditions used in the manuscript to stimulate cell migration like fMLP treatment.

-The authors use an artificial tether to manipulate mito-ER contacts in cellulo. However, no information from its origin, or its design are documented in the manuscript. In addition, the authors should show that this tether efficiently works by analysing mito-ER contacts upon expression by EM and mitochondrial calcium uptake. Does this tether rescue mito-ER contacts defects induced by loss of Mfn2? How the authors explain that the tether rescues mitochondrial morphology defects in MFN2KO? In these conditions, mitochondria should not be able to fuse anymore as Mfn2 is lost? This is really intriguing results. Does the tether rescue the other parameters? Mitochondrial distribution (with quantification)? Cell shape?
Paxillin defects? ROS and membrane potential? These rescue experiments analyses are important to determine which parameters are really involved in cell migration defects due to the decreased tethering. Finally, it would be of great interest to analyse the effect of the tether alone on cell migration, Rac1 activity, cell shape? Gain of function? These results may reinforce the idea that contact sites regulate cell migration.

-It is well established that a decrease of membrane potential leads to a decrease of mitochondrial calcium uptake. Calcium results obtained by the authors without any information on the roles of the tether could not lead to any conclusion. Does the tether rescues membrane potential and calcium uptake by the mitochondria? So far, the decrease of mitochondrial calcium upon stimulation in Mfn2KO cab be attributed to both mito-ER contact or membrane potential defects. It has been shown that MEFs MFN2 KO can lead to a decrease of MCU provel level leading to a decrease of mitochondrial calcium uptake (PMID: 25870285). The authors should also check MCU protein level.

-Hyperactivation of Rac1 is only based on phosphorylation of PAK, which is quite weak. The authors should better describe the hyperactivation of RAC1 or other RhoGTPases in their Mfn2 KO MEFs. What are the levels of RAC1 and other RhoGTPases? Subcellular distribution in the cell? Kits are also available to determine RhoGTPase activity by pull down assay (Cell biolabs).

-The references are up-to-date. The text and the figures are clear and accurate.

Minor comments:

-The authors should show the efficiency of the KO generated for Mfn2 and Opa1 in zebrafish embryos. Sequencing results to highlight the position of the mutations and their consequences on the coding protein should be shown, as well as immunoblot analysis should be performed to analyse Mfn1, Mfn2 and OPA1 protein levels. The generation of a MFN1-KO transgenic line would have been appreciated to finely compare the roles of the 3 GTPases involved in mitochondrial fusion during neutrophil infiltration and migration in vivo.

-MFN1, MFN2, AND OPA1 protein levels should be analysed by immunoblot in the Mfn1 and Mfn2 KO MEFs.

-In cell spreading assay, it would be great to identify cells during the process, by an asterix for example. "wt MEFs extended transient filopodia and lamellipodia and eventually elongated, whereas Mfn2-null MEFs generated extensive membrane ruffles and retained the circular shape". It would be interesting to quantify these different parameters.

© 2020. Published by The Company of Biologists under the terms of the Creative Commons Attribution License (https://creativecommons.org/licenses/by/4.0/).

-For all their immunoblot analysis, the authors should use a mitochondrial marker as loading control (VDAC1, TOM20, HSP60...). In figure 5, Vinculin should not be used a loading control, with its role in focal adhesion dynamics.

-Legends for figures 5 and 6 are inverted.

-Please document in the material and methods section, how confocal images have been acquired: number of z-stacks, reconstitution, 3D analysis...

-The authors should show their results of blood cell composition quantification in ctrl vs MFn2 depletion.

-The authors should describe all the acronyms used throughout all the manuscript. For example, LTB4, fMLP...

Significance

Beyond their role in energy production, mitochondria are involved in numerous cellular functions including cell migration. Mitochondria form a network balanced by fission and fusion events, where membrane contact sites with the endoplasmic reticulum are crucial. These contact sites are also involved in mitochondrial and cellular functions via their capacity to exchange lipids, metabolites and calcium. The role of mitochondria in cell migration has started to emerge where mitochondrial fragmentation and/or mitochondrial calcium homeostasis are acknowledged to drive cancer cell migration and to regulate actin dynamics.

In this manuscript, Zhou W and colleagues proposed for the first time the role of mitochondria-ER contacts in cell migration. Mechanistically, this can be associated to the capacity of these contacts to control mitochondrial functions or mitochondrial calcium homeostasis. These findings are physiologically relevant and of particular interest to the mitochondrial and cell migration field but also to general cell biology. It represents a novel function associated to these membrane contact sites and point-out these contacts as signalling platform creating microdomains of metabolites exchanges involved in cell migration.

Keywords: Mitochondria - Membraned dynamics - calcium homeostasis - Membrane contact sites

Reviewer 3

Evidence, reproducibility and clarity

Mitofusin 2 (Mfn2) is a mitochondrial outer membrane protein that is important for mitochondrial fusion and the establishment of mitochondrial ER contacts. It has been published before that these contact sites are important for calcium signaling. Zhou et al. examined the role of Mfn2 in neutrophils. They propose a model in which mitochondrial ER contacts established via Mfn2 are crucial for regulation of Rac, which is a small GTPase driving cell migration by promoting actin polymerization. Loss of Mfn2 results in elevated cytosolic calcium, over-activation of Rac, and defects of chemotactic movements. These defects can be partially rescued by restoration of mitochondrial ER contacts through expression of an artificial tether protein.

Major points

1.The authors claim on p. 6 that decreased neutrophil retention is not simply due to defects in mitochondrial fusion. However, the experimental setup they used for mfn2 (Fig. 1) is different from that for opa1 (Fig. S1), and therefore the results are not directly comparable. Unfortunately, the authors don't show fragmentation of mitochondria, neither in mfn2 nor in opa1 depleted cells. To support their statement they must show that lack of Mfn2 and Opa1 causes mitochondrial fragmentation to the same extent and then examine neutrophil retention in the same assay. Also, it would make sense to include Mfn1 in this analysis, as the authors later claim that the effects they observed are specific for Mfn2.

© 2020. Published by The Company of Biologists under the terms of the Creative Commons Attribution License (https://creativecommons.org/licenses/by/4.0/).

2.The authors should examine mitochondrial morphology in MFN2 shRNA treated cells (Fig. 2) and in mfn2-null MEFs (Fig. 3).

3.The authors claim that chemotaxis defects of neutrophils are specific for MFN2 knock down, but not for MFN1. They show a Western blot of mfn1 knock down cells in Figure S3s. There is a band clearly visible, which appears to be much stronger than the MFN2 band in sh1 cells in Fig. 2a. Therefore, this conclusion is not valid.

4.The colocalization of MFN2 with mitochondria and ER, shown in Fig. 4a, should be improved. Both mitochondria and ER appear abnormally clumped. The authors should stain mitochondria, ER and Mfn2 in the same cells. Images should be displayed much larger. The same is true for Fig. 5a. The authors claim that an artificial tether restored mitochondrial morphology in mfn2 knock down cells. They should state in the text which tether was used. Furthermore, they should explain their criteria for judgement of mitochondrial morphology. At least in my exes, mitochondria appear highly clumped in the image shown for sh1+T cells. In Fig. 5c it is not indicated how many cells were scored.

Minor points

5.The Western blot shown in Fig. 5d suggests that expression of the tether construct reduced the amount of MFN2. How can this be explained?

6.The paper is sometimes hard to digest for a reader who is not familiar with the authors' experimental systems. The description of the experiments in the main text is highly condensed.

7.Page 11: "5 m post stimulation" should read "5 min post stimulation".

8.Some references are incomplete (page numbers are lacking).

Significance

Apparently, the manuscript is written for an audience with a special interest in chemotactic movements of neutrophils. I guess that the results reported in this manuscript will be of interest for this field. My background is mitochondrial biology and dynamics and I don't have the expertise to evaluate the aspects specific for neutrophils.

Author response to reviewers' comments

I thank the referees for their enthusiasm and time providing critical feedbacks to our manuscript. The novelty of our work is the identification of the importance of Mfn2 in regulating the Rac signaling and neutrophil migration& adhesion, which is significantly relevant to the mitochondrial field and cell biology in general. Below please find our point-to-point response to the comments.

Reviewer #1 (Evidence, reproducibility and clarity (Required)):
Introduction:
"Although mitochondria-derived ATP possibly regulates neutrophil chemotaxis in vitro (Bao et al., 2015), removal of extracellular ATP improves neutrophil chemotaxis in vivo (Li et al., 2016). These conflicting reports prompted us to search for mechanisms delineating the role of mitochondria in neutrophil migration outside the realm of ATP or cellular energy (Bi et al., 2014; Schuler et al., 2017; Zanotelli et al., 2018)."
This sentence is superficial and misleading: extracellular ATP may interfere with chemotaxis through various energy-independent mechanisms (see for example Zumerle et al. Cell Reports 2019) and this is not conflicting with the role of intracellular ATP in migration.

© 2020. Published by The Company of Biologists under the terms of the Creative Commons Attribution License (https://creativecommons.org/licenses/by/4.0/).

We were not clear in the writing that Bao et al suggest that neutrophils secret ATP at the leading edge and mitochondria at the leading edge is the source of the extracellular ATP. Both studies focused on extracellular ATP. We agree that the reports are not necessarily conflicting since exogenous ATP can induce additional signaling. We rewrote this sentence emphasizing that we are looking for mechanisms in addition to ATP, which is distinct from previous studies.

Figure 1:
The authors didn't show evidence of the genome edition (PCR, RFLP or Sequencing over the sgRNA target) or at least RT-PCR or WB for MFN2.
In Fig 1b, 1c the scale bar is missing.
"Neutrophils were sorted from both lines and their respective loci targeted by the 4 sgRNAs were deep sequenced." There are no data about sorting strategies for zebrafish neutrophils in the figure. Moreover, only 2 sgRNAs are shown and there are no sequencing data.

To show evidence of the genome edition, we have deep sequenced this loci of mfn2 and opa1 and the mutation frequencies were stated in the original text. The sorting strategies were described in Methods-Mutational Efficiency Quantification. Each mfn2 KO has 2 individual sgRNAs, and two KO (mfn2 KO and mfn2 KO#2) were shown in Fig 1b, so there are 4 sgRNAs targeting mfn2. Since each embryos have approximately 150 neutrophils, WB is not feasible. Sequencing is the standard method (Ablain et al., 2015; Zhou et al., 2018). We only stated the mutation efficiency in the manuscript because amplifying the genomic DNA from the sorted cells introduces PCR bias and the numbers are not a quantitative reflection of the degree of gene disruption. We will include the sequencing result of the sgRNA target sites in a supplemental Figure.

We used one scale bar for all the panels in Fig 1b,c. All panels are at the some magnification.

Figure 2:
In the WB, reconstitution is not obvious. In general, all WBs are not quantified (and they should be quantified).
The in vivo experiment does not have proper controls. For example, can the authors exclude that in these mice there is reduced inflammation because neutrophils have defective activation? What about NETs? And cytokines/chemokines? And exocytosis? In the absence of these controls, the experiment cannot be properly interpreted.

We have quantified all WBs in our study. The results were sometimes stated in the text only. We will add the quantifications to each blot.
The mice model we chose is used to evaluate in vivo neutrophil migration. We used a neutrophil specific promoter to delete mfn2 in mice and collected data at a very early time point when the tissue inflammatory environment is determined by tissue resident sentinel cells, such as macrophages. Although our results support that mfn2 is required for neutrophil migration in mammals, we agree that we can not fully rule out that other neutrophil functions are also regulated by mfn2.

To address whether other neutrophil functions are affected by MFN2, we will performed assays to evaluate NETosis and degranulation in MFN2 KD HL-60 cells to evaluate the other neutrophil functions.

Figure 3:
The conclusion of the authors "In summary, Mfn2 modulates the actin cytoskeleton and cell migration in MEFs" should be supported by experiments to distinguish between the specific role of Mfn2 and the role of mitochondrial dynamics (Opa1, Drp1, Mfn1). It is also not clear why the authors decided to use MEFs instead of other cells (more similar to neutrophils which are not adherent cells). The results obtained in MEFs may be irrelevant for neutrophils.

We agree that MEFs are very different from neutrophils. We chose MEFs since the function of Mfn2 in MEF is well characterized (Chen et al., 2003; de Brito and Scorrano, 2008; Naon et al., 2016). Both Mfn1 and Mfn2 MEF have fragmented mitochondria. Mfn1, which is very similar to Mfn2, serves as the best control. We will confirm the mitochondria structure in the KO cells.

© 2020. Published by The Company of Biologists under the terms of the Creative Commons Attribution License (https://creativecommons.org/licenses/by/4.0/).

For specificity, in addition to mfn2, we looked at Mfn1 and opa1 in different systems. We did not select Drp1 since the mitochondrial network in neutrophils is highly fused (Fig 4 and 5)(Maianski et al., 2002; Zhou et al., 2018).

We have also knocked down Opa 1 in HL-60s. We observed massive cell death in this line and cell migration is affected, possibly due to a depletion of cellular ATP as reported (Amini et al., 2018). We will include the data showing cell death, qRT to show knockdown efficiency and chemotaxis. In zebrafish neutrophils, knocking out Opa1 also reduced cell migration (Fig 1S).

Figure 4-5:
Fig 5a: in ctrl and sh1 the ER seems to be larger than the phalloidin (=cytoskeleton=cell border approximately) in a few regions. Only the sh1+T seems to fit correctly.

We use the F-actin staining as an indicator of cell front. F-Actin is predominant at cell front, but much less in the cell body and uropod. Here we set the confocal laser power at a certain level to give us a good resolution of brighter signals which may not be strong enough to detect signals in the cell body. That's why the fluorescence is very dim or even absent in the cell body. However, the majority of ER do fit in the cell border if look closer.

The TEM image (only 1 in supplementary) is not sufficient to convince that the tethering is lost. Quantification of number of contacts and distance between ER and mitochondria should be included.

Using EM method, *Mfn2* ablation decreases the ER-contacting mitochondrial surface by ~20–35% (Naon et al., 2017). Using the same cells, different groups reached different conclusions using TEM(Filadi et al., 2017). We reason that ER-mitochondria contact sites are rare events in TEM since the samples are sliced. We will try to take more TEM images to quantify the distance. However, we are not sure that we can come up with a definitive conclusion by TEM. Nevertheless, we observed significant mitochondrial structural changes using IF and observed the changes in cytosolic calcium levels, which is consistent with the known function of Mfn2 as a ER-mitochondrial tether (Naon et al., 2016).

The title of figure 5 is wrong.
However, in these figures, it is clear that cells are beautifully polarized, with mitochondria accumulating at the uropod (and even more in the absence of Mfn2). When comparing these images with those published by Campello et al (JEM 2006), there are 2 observations that can be made: first of all, these data confirm that mitochondrial fission promotes cell polarity; second, they suggest that the defect is not at the level of cell polarity/chemotaxis.

We have fixed the title of figure 5.

We agree that mfn2 defective neutrophils does not have a defect in cell polarization. The defects in migration is possibly due to other reasons such as poor adhesion or regulation in the actin cytoskeleton dynamics. However, our data is not sufficient to support that mitochondrial fission promotes cell polarization and chemotaxis.

Figure 6:
Calcium data are, in general, very weak. First of all, controls with ionomycin are missing. Statistical analyses of the curves should be included. As for the use of the MCU inhibitor Ru360, is there any evidence that it is cell-permeant in this context? Is it blocking MCU? Since the authors can show mitochondrial calcium upon FMLP, they should also demonstrate that Ru360 is indeed working and inhibiting mitochondrial calcium uptake.
The sentence "The MCU inhibitor Ru360did not cause further reduction of chemotaxis in MFN2 knockdown dHL-60 cells (Supplementary Fig. 6c, d and Supplementary Movie. 12), indicating that MCU and MFN2 lies in the same pathway in terms of regulating chemotaxis in dHL-60 cells" is speculative.
In general, there is no solid demonstration that the effect is calcium-mediated.

We will include the control of ionomycin and include statistics of the results.

© 2020. Published by The Company of Biologists under the terms of the Creative Commons Attribution License (https://creativecommons.org/licenses/by/4.0/).

Ru360 is a widely used MCU inhibitor. The fact that Ru360 itself inhibited neutrophil migration supported that the chemical enters cells. We agree that stating "indicating that MCU and MFN2 lies in the same pathway in terms of regulating chemotaxis in dHL-60 cells" is speculative. In addition, we tried to reduce cytosolic calcium levels in mfn2 KD cells either using Ca^{2+} chelator (BAPTA, in Fig S6) or an IP3 receptor inhibitor. In both cases we observed reduced migration blocking calcium signal alone. The mfn2 KD phenotype was not rescued. This could due to that multiple molecules/pathways are calcium dependent in cell migration. We will include all the negative data. We thus far are still unable to establish a functional link of the calcium with mfn2 regulated signaling.

We have moved the calcium data to Fig 4. The elevated calcium signal is an indirect evidence to support the loss of ER-mitochondria tether. We have modified our conclusion to leave out calcium as a relevant signal regulated by mfn2 for neutrophil migration.

As for Rac, it is surprising to see that Rac inhibition has no effect on cell migration. Rac is known to promotes migration in fibroblasts and other cell types and Rac deficiency inhibits migration (see for example Steffen et al, JCS 2013). Two sets of experiments are absolutely required: 1) verify this in fibroblasts since it has been elegantly shown that Rac is essential in these cells for migration; 2) analyse the effect of Rac inhibitors in pPak kinetics.

Rac is required for cell migration and the growth of branched actin network. The Rac inhibitors we selected here are specific to two rac GEFs, vav and Tiam. Steffen et al, JCS 2013 used Rac1 KO MEF, which is different from ours. Thus the works are not contradictory. MEFs are very different from neutrophils. We chose MEFs since there are knockout cells available and well characterized. The MFN2 KO cells display prominent lamellipodia, which is also consistent with the observation in Steffen et al, JCS 2013. We have used these two inhibitors in MEF wound closure and did not observe a strong phenotype.

We will analyze the effect of Rac inhibitors in pPak kinetics in the control and Mfn2 deficient dHL-60 cells.

Reviewer #1 (Significance (Required)):

As presented here, the manuscript has a modest significance. The audience would be specialised: cell migration, cell signalling.
My expertise is immunology, cell activation, cell migration, cell signalling.

Reviewer #2 (Evidence, reproducibility and clarity (Required)):

Major comments:

Although the results could be very interesting, and could be significantly relevant to the mitochondrial field and the cell biology one in general, major points need to be addressed to fully support conclusions of the authors. Different controls and quantification are missing, Actin dynamics analysis should be improved, effects of the artificial tether is weakly characterized and the demonstration of the specific role of mito-ER contacts via mfn2 in migration should be reinforced.

-In figure 1, quantification of circulating neutrophils is required in Mfn2 KO embryos. The authors should also show these quantified results for OPA1KO, which are just mentioned in the text. In addition, in figure 1b and d, the neutrophils from the Mfn2KO embryos seem bigger compared to control. Can the authors comment on neutrophils size and potential contribution to the phenotype? Finally, the authors propose a defect in neutrophil migration in Mfn2-KO, however neutrophils are found in the circulation. The authors should explain these results.

Since the cells are all in circulation, we can only estimate the percentage. Overall, the phenotype is drastic, shown in movie S1. We will state how many fish embryos we have imaged and how often we observe this phenotype (only 1 or 2 in the tissue (mfn2 KO) or in circulation (control)). The bigger spots are resulted from cells outside the focal plane-zebrafish embryos

© 2020. Published by The Company of Biologists under the terms of the Creative Commons Attribution License (https://creativecommons.org/licenses/by/4.0/).

are thick tissues. We agree that since neutrophils in the KO fish are all circulation, we cannot make a conclusion whether they can migrate in tissue in zebrafish. We conclude that “mfn2 regulates neutrophil tissue retention and extravasation in zebrafish”, but did not comment on chemotaxis.

-The authors need to reinforce the Mfn2 specificity for their phenotype. In particular in Fig S1, they show that loss of OPA1 significantly decreases neutrophil migration in vivo. However, they then only study the effect of Mfn1 silencing in neutrophil and MFN1 KO MEFs (Sup Fig s3). The authors should perform the same experiments in neutrophil and MEF upon loss of OPA1 (similar to Fig S3). Does loss of OPA1 and Mfn1 decrease neutrophil arrest to activated endothelial cells?

We knocked down OPA1 in HL-60 cells. The cells appear unhealthy and display a migration defect, consistent with the data in zebrafish. We are not comfortable making conclusions here since secondary affects in dying cells may cause any phenotype not directly attributed to the loss of OPA1. Nevertheless, we will include the data.

We have decided not to include Opa1 KO MEF since the cell morphology as documented in ATCC is similar to that of WT MEF. Only the MEF2 KO MEF is more circular. MFN1 KO MEF is a better specificity control which we have characterized in depth.

[*NOTE: We have removed images from ATCC documenting the morphology of MEF cells in culture.*

The information can be accessed here:
OPA1 KO MEFs, https://www.atcc.org/products/all/CRL-2995.aspx#characteristics
Mfn2-null MEFs, https://www.atcc.org/products/all/CRL-2993.aspx#characteristics
WT MEFs, https://www.atcc.org/products/all/CRL-2907.aspx#characteristics]

Since Mfn1 KD HL-60 cells migrate well on surface, they are not expected to have adhesion defects. Nevertheless, we will determine whether loss of Mfn1 decrease neutrophil arrest to activated endothelial cells and include the data.

-Using their images, the authors should also document on the directionality of the cell during cell migration. Do Mfn2 depleted cells do not migrate because they are arrested or because they are lacking directionality? Environment/chemokine sensing defects?

We will quantified the directionality of the cells. As pointed out by reviewer 1, mfn2 deficient cells can polarize and not defective in chemokine sensing. We do not expect a significant change in directionality defect.

-Actin dynamics analysis should be improved. Loss of Mfn1 and Mfn2 lead to cell shape changes. The authors should quantify this phenotype by analysing cell circularity (as well as for Opa1 loss). Stress fibres number or Phalloidin intensity quantification in cell body should also be performed.

We will quantified the circularity, stress fiber numbers and phalloidin intensity in Mfn1 and Mfn2 KO MEFs.

-Can the migration defects could be attributed to Focal adhesion protein dynamics defects? The authors shown an hyperactivation of Rac1 and an hyperphosphorylation of PAK, which can control FAP (focal adhesion proteins) dynamics. In addition, immunofluorescence analysis shows a decreased signal and cellular misdistribution of paxillin. The authors should characterize these phenotypes. FAP levels (Paxillin/Phospho-Paxillin and Vinculin) should be analysed by immunoblot, the number of FAP/cell, distribution and size should also be quantified. Their dynamics should also be analysed by live cell imaging. Finally, Paxillin level and distribution seems to be also impacted in Mfn1KO cells. Can the authors comment on that? The different quantifications would help to better understand the effect of different mitofusins in cytoskeleton dynamic.

© 2020. Published by The Company of Biologists under the terms of the Creative Commons Attribution License (https://creativecommons.org/licenses/by/4.0/).

We thank the reviewer for the great advices for our follow up work. So far our results supports Rac over activation as a relevant pathway how mfn2 regulates neutrophil migration. Although Rac can regulate focal adhesion dynamics in other cells (Rooney et al., 2010), how Rac activation regulates focal adhesion dynamics in neutrophils is not clear. Mfn2 regulated membrane tether could affect lipid trafficking, cellular metabolism and other signaling molecules. It will take substantial amount of work to make a conclusion and it is more suitable a separate report. This is one of the directions we will pursuit in our future studies.

-Please perform rescue experiments for cell migration in MFN2KO and MFN1KO MEFs. Immunoblots showing protein levels of these proteins would be appreciated. To really discriminate how Mfn2 regulates cell migration, the authors should also perform rescue experiments using a fusogenic mutant Mfn2 ((K109A). It will help to demonstrate the relevance of mito-ER contacts and not mitochondrial fusion in the phenotype.

For the reason mentioned above, we do not plan to do additional experiments in MEF cells since this work is focused on neutrophils. It is documented that Mfn2 K109A cannot restore mitochondrial fusion. However, it is not clear whether this construct can restore ER-tether. Result using this construct will be hard to interpret.

-Figure 4, the authors stipulate that Mfn2 regulates ER-mitochondria tethering. However, the authors present no evidence for this conclusion. The authors should perform manders coefficient in MFN2 KO cells and compared it to control. Also, loss of Mfn2 induces mitochondrial fragmentation, which can lead to problem for mito-er contacts quantification by light microscopy. The authors should use their TEM pictures to quantify mito-ER contacts (Number, length and % of mito perimeter), not only mitochondrial morphology. Mfn1 should be used as negative control. it would be interesting also to determine the status of the mito-ER contact in the different conditions used in the manuscript to stimulate cell migration like fMLP treatment.

We have performed manders coefficient in the mfn2 KD cells and observed no difference compared with the control. It is possibly due to the prevalent ER structure in the cells-despite the structural change, mitochondria are still mostly on top of ER when examined using IF. Using EM method, *Mfn2* ablation decreases the ER-contacting mitochondrial surface by ~20–35% (Naon et al., 2017). Using the same cells, different groups reached different conclusions using TEM(Filadi et al., 2017). We reason that ER-mitochondria contact sites are rare events in TEM since the samples are sliced. We will try to take more TEM images to quantify the distance. However, we are not sure that we will come up with a definitive conclusion by TEM. Nevertheless, we observed significant mitochondrial structural changes using IF and observed the changes in cytosolic calcium levels, which is consistent with the known function of Mfn2 as a ER-mitochondrial tether (Naon et al., 2016).

-The authors use an artificial tether to manipulate mito-ER contacts in cellulo. However, no information from its origin, or its design are documented in the manuscript. In addition, the authors should show that this tether efficiently works by analyzing mito-ER contacts upon expression by EM and mitochondrial calcium uptake. Does this tether rescue mito-ER contacts defects induced by loss of Mfn2? How the authors explain that the tether rescues mitochondrial morphology defects in MFN2KO? In these conditions, mitochondria should not be able to fuse anymore as Mfn2 is lost? This is really intriguing results. Does the tether rescue the other parameters? Mitochondrial distribution (with quantification)? Cell shape? Paxillin defects? ROS and membrane potential? These rescue experiments analyses are important to determine which parameters are really involved in cell migration defects due to the decreased tethering. Finally, it would be of great interest to analyse the effect of the tether alone on cell migration, Rac1 activity, cell shape? Gain of function? These results may reinforce the idea that contact sites regulate cell migration.

The tether is a GFP protein carrying both ER and mitochondrial localization sequences at the ends (Kornmann et al., 2009). The details are now added to the manuscript.

© 2020. Published by The Company of Biologists under the terms of the Creative Commons Attribution License (https://creativecommons.org/licenses/by/4.0/).

In HL-60 mfn2 KD cells, tether expression partially rescues mitochondrial distribution (quantified in Fig 5c), cell migration and Rac over activation. Although ROS and membrane potential are slightly affected by Mfn2 deletion in HL-60 cells, it is not clear whether they play any roles in mfn2 regulated cell adhesion or migration. We will attempt to use TEM to determine the mitochondrial structure upon tether rescue.

Despite multiple attempts, we could not obtain a line over-expressing the tether in wt HL-60 cells. We suspect that further increase in the tether is toxic to the cells.

-It is well established that a decrease of membrane potential leads to a decrease of mitochondrial calcium uptake. Calcium results obtained by the authors without any information on the roles of the tether could not lead to any conclusion. Does the tether rescues membrane potential and calcium uptake by the mitochondria? So far, the decrease of mitochondrial calcium upon stimulation in Mfn2KO cab be attributed to both mito-ER contact or membrane potential defects. It has been shown that MEFs MFN2 KO can lead to a decrease of MCU provel level leading to a decrease of mitochondrial calcium uptake (PMID: 25870285). The authors should also check MCU protein level.

We observed that mfn2 deficiency resulted in a minor reduction in membrane potential. Although Mfn2 KO MEF has reduced level of Mcu, Mfn2 silence in MEF does not affect Mcu levels (Filadi et al., 2015). Another group also concluded that Mfn2 deletion does not necessarily affect Mcu levels (Naon et al., 2016). Nevertheless, we will measure the MCU protein level in the Mfn2 knockdown HL-60 cells.

-Hyperactivation of Rac1 is only based on phosphorylation of PAK, which is quite weak. The authors should better describe the hyperactivation of RAC1 or other RhoGTPases in their Mfn2 KO MEFs. What are the levels of RAC1 and other RhoGTPases? Subcellular distribution in the cell? Kits are also available to determine RhoGTPase activity by pull down assay (Cell biolabs).

In Mfn2 KO MEFs, Rac overactivation is suggested by the increased lamellipodia formation, classical Rac readouts. Since the current manuscript focuses on neutrophils, we will performed the Rac GFP pull down experiments in HL-60 cells. We will also stain Rac GTP in HL-60 cells.

-The references are up-to-date. The text and the figures are clear and accurate.

Minor comments:

-The authors should show the efficiency of the KO generated for Mfn2 and Opa1 in zebrafish embryos. Sequencing results to highlight the position of the mutations and their consequences on the coding protein should be shown, as well as immunoblot analysis should be performed to analyse Mfn1, Mfn2 and OPA1 protein levels. The generation of a MFN1-KO transgenic line would have been appreciated to finely compare the roles of the 3 GTPases involved in mitochondrial fusion during neutrophil infiltration and migration in vivo.

To show evidence of the genome edition, we have deep sequenced this loci of mfn2 and opa1 and the mutation frequencies were stated in the original text. Since each embryos have approximately 150 neutrophils, WB is not feasible. Sequencing is the standard method (Ablain et al., 2015; Zhou et al., 2018). We only stated the mutation efficiency in the manuscript because amplifying the genomic DNA from the sorted cells introduces PCR bias and the numbers are not a quantitative reflection of the degree of gene disruption. We will include the sequencing result of the sgRNA target sites in a supplemental Figure.

The *mfn1* gene in zebrafish is duplicated. We are not sure whether we can obtain efficient disruption at both loci. We hope the results using Mfn1 KO MEF and MFN1 KD HL-60 cells are enough to show a specific role of Mfn2 in cell migration.

-MFN1, MFN2, AND OPA1 protein levels should be analysed by immunoblot in the Mfn1 and Mfn2 KO MEFs.

© 2020. Published by The Company of Biologists under the terms of the Creative Commons Attribution License (https://creativecommons.org/licenses/by/4.0/).

It is unlikely that mfn1/2 KO will affect OPA1 levels (Saita et al., 2016). Both MFN1 and MFN2 MEF display fragmented mitochondrial network which can be rescued by overexpression of MFN1 or MFN2 (Chen et al., 2003). The level of OPA1 in the cells are not relevant. We will stain mitochondria in the mfn1/2 KO MEFs to make sure that the cells have fragmented mitochondria as expected.

-In cell spreading assay, it would be great to identify cells during the process, by an asterix for example. "wt MEFs extended transient filopodia and lamellipodia and eventually elongated, whereas Mfn2-null MEFs generated extensive membrane ruffles and retained the circular shape". It would be interesting to quantify these different parameters.

We will add Asterixes to the cells. We will quantify the percentage of cells that can rearrange their cell shape in the WT and Mfn2 KO MEFs.

-For all their immunoblot analysis, the authors should use a mitochondrial marker as loading control (VDAC1, TOM20, HSP60...). In figure 5, Vinculin should not be used a loading control, with its role in focal adhesion dynamics.

Vinculin is stable in HL-60 cells under multiple conditions and selected as a control. The signal intensity correlates well with the amount of protein loaded. Using mitochondrial proteins as loading controls is not common and may be risky as the amount of mitochondria in cells can be variable.

-Legends for figures 5 and 6 are inverted.

Thanks, we have changed the heading of figure 5. The legends were correct.

-Please document in the material and methods section, how confocal images have been acquired: number of z-stacks, reconstitution, 3D analysis...

We will update in the method the parameters of imaging acquisition.

-The authors should show their results of blood cell composition quantification in ctrl vs MFn2 depletion.

We will include the results of blood cell composition in a supplemental figure.

-The authors should describe all the acronyms used throughout all the manuscript. For example, LTB4, fMLP...

We have describe all the acronyms in the updated manuscript.

Reviewer #2 (Significance (Required)):

Beyond their role in energy production, mitochondria are involved in numerous cellular functions including cell migration. Mitochondria form a network balanced by fission and fusion events, where membrane contact sites with the endoplasmic reticulum are crucial. These contact sites are also involved in mitochondrial and cellular functions via their capacity to exchange lipids, metabolites and calcium. The role of mitochondria in cell migration has started to emerge where mitochondrial fragmentation and/or mitochondrial calcium homeostasis are acknowledged to drive cancer cell migration and to regulate actin dynamics. In this manuscript, Zhou W and colleagues proposed for the first time the role of mitochondria-ER contacts in cell migration. Mechanistically, this can be associated to the capacity of these contacts to control mitochondrial functions or mitochondrial calcium homeostasis. These findings are physiologically relevant and of particular interest to the mitochondrial and cell migration field but also to general cell biology. It represents a novel function associated to these membrane contact sites and point-out these contacts as signalling platform creating microdomains of metabolites exchanges involved in cell migration.

Keywords: Mitochondria - Membraned dynamics - calcium homeostasis - Membrane contact

© 2020. Published by The Company of Biologists under the terms of the Creative Commons Attribution License (https://creativecommons.org/licenses/by/4.0/).

sites

Reviewer #3 (Evidence, reproducibility and clarity (Required)):

Mitofusin 2 (Mfn2) is a mitochondrial outer membrane protein that is important for mitochondrial fusion and the establishment of mitochondrial ER contacts. It has been published before that these contact sites are important for calcium signaling. Zhou et al. examined the role of Mfn2 in neutrophils. They propose a model in which mitochondrial ER contacts established via Mfn2 are crucial for regulation of Rac, which is a small GTPase driving cell migration by promoting actin polymerization. Loss of Mfn2 results in elevated cytosolic calcium, over-activation of Rac, and defects of chemotactic movements. These defects can be partially rescued by restoration of mitochondrial ER contacts through expression of an artificial tether protein.

Major points

1.The authors claim on p. 6 that decreased neutrophil retention is not simply due to defects in mitochondrial fusion. However, the experimental setup they used for mfn2 (Fig. 1) is different from that for opa1 (Fig. S1), and therefore the results are not directly comparable. Unfortunately, the authors don't show fragmentation of mitochondria, neither in mfn2 nor in opa1 depleted cells. To support their statement they must show that lack of Mfn2 and Opa1 causes mitochondrial fragmentation to the same extent and then examine neutrophil retention in the same assay. Also, it would make sense to include Mfn1 in this analysis, as the authors later claim that the effects they observed are specific for Mfn2.

Since Mfn2 KO neutrophils are not in tissue, the experiment in Fig 1S to look at cell speed in tissue is not feasible. Since the cells are all in circulation, we can only estimate the percentage. Overall, the phenotype is very drastic, see movie S1. We will state how many fish embryos we have imaged and how often we observe this phenotype (only 1 or 2 in the tissue (mfn2 KO) or in circulation (control and opa1 KO)). Opa1 KO neutrophils are not in circulation.

To show evidence of the genome edition, we have deep sequenced this loci of mfn2 and opa1 and the mutation frequencies were stated in the original text. Since each embryos have approximately 150 neutrophils, WB and other biochemical assays are not feasible. Sequencing is the standard method (Ablain et al., 2015; Zhou et al., 2018). We only stated the mutation efficiency in the manuscript because amplifying the genomic DNA from the sorted cells introduces PCR bias and the numbers are not a quantitative reflection of the degree of gene disruption. We will include the sequencing result of the sgRNA target sites in a supplemental Figure.

Since Mfn2 KO neutrophils are all in circulating, we cannot observe their mitochondrial morphology. This is the reason why we used HL-60 cells for the mechanistic study. The mfn1 gene in zebrafish is duplicated. We have generated an mfn1b KO line and did not observe any phenotype. However we are not sure whether we can obtain efficient disruption at both loci. We hope the results using Mfn1 KO MEF and MFN1 KD HL-60 cells are enough to show a specific role of Mfn2 in cell migration.

We will have stained the mitochondria structure in the MEF1/2 MEF cells and the in Mfn1/2 KD dHL-60 cells. Opa1 KD HL-60 cells display extensive cell death and we are not confident interpreting any results from this line.

2. The authors should examine mitochondrial morphology in MFN2 shRNA treated cells (Fig. 2) and in mfn2-null MEFs (Fig. 3).

Mitochondrial morphology is examined in MFN2 shRNA treated cells (Fig 4c and 5a). The mitochondrial morphology in mfn2-null MEFs are published (Chen et al., 2003). We will further confirmed their results by staining mitochondrial structure in the KO MEFs.

3. The authors claim that chemotaxis defects of neutrophils are specific for MFN2 knock down, but not for MFN1. They show a Western blot of mfn1 knock down cells in Figure S3s. There is a

© 2020. Published by The Company of Biologists under the terms of the Creative Commons Attribution License (https://creativecommons.org/licenses/by/4.0/).

band clearly visible, which appears to be much stronger than the MFN2 band in sh1 cells in Fig. 2a. Therefore, this conclusion is not valid.

The band intensities are dependent on the antibody quality and imaging acquisition and display. We don’t feel comfortable comparing the amount of two different proteins from two separate blots.

4. The colocalization of MFN2 with mitochondria and ER, shown in Fig. 4a, should be improved. Both mitochondria and ER appear abnormally clumped. The authors should stain mitochondria, ER and Mfn2 in the same cells. Images should be displayed much larger. The same is true for Fig. 5a. The authors claim that an artificial tether restored mitochondrial morphology in mfn2 knock down cells. They should state in the text which tether was used. Furthermore, they should explain their criteria for judgement of mitochondrial morphology. At least in my exes, mitochondria appear highly clumped in the image shown for sh1+T cells. In Fig. 5c it is not indicated how many cells were scored.

We will replace Fig 4a with a more representative image.

Neutrophils are blood cells and do not spread as well as adherent cells. We have also overexposed the images to show the smaller mitochondria, which cannot be visualized without saturating the signals. We tried to stain the cells with Mfn2 and Calnexin. However we cannot retain the mitotracker signal in fixed cells and could not do a triple label in dHL-60 cells. For this reason we have done double staining of mitochondria-ER, MFN2-mitochondria and MFN2-ER.

We have included the citation and the description of the tether. The tether is composed of a GFP protein carrying both ER and mitochondrial localization sequences at the ends, which functions independently of MFN2.

The criteria for the judgement of the mitochondrial morphology is now included in the methods, clustering analysis.

Minor points

5. The Western blot shown in Fig. 5d suggests that expression of the tether construct reduced the amount of MFN2. How can this be explained?

This Mfn2 amount is not significantly altered by the tether expression when quantified. We will add the quantification of all WB to the figures.

6. The paper is sometimes hard to digest for a reader who is not familiar with the authors' experimental systems. The description of the experiments in the main text is highly condensed.

We will elaborate on the experimental system in the results section.

7. Page 11: "5 m post stimulation" should read "5 min post stimulation".

Thank you. We have made this correction.

8. Some references are incomplete (page numbers are lacking).

We will reformat our references and checked for page numbers.

Reviewer #3 (Significance (Required)):

Apparently, the manuscript is written for an audience with a special interest in chemotactic movements of neutrophils. I guess that the results reported in this manuscript will be of interest for this field. My background is mitochondrial biology and dynamics and I don't have the expertise to evaluate the aspects specific for neutrophils.

© 2020. Published by The Company of Biologists under the terms of the Creative Commons Attribution License (https://creativecommons.org/licenses/by/4.0/).

It is well established that mfn2 mediates mitochondrial fusion and ER contact. Our novelty is the discovery that mfn2 suppresses Rac activation, which is essential for neutrophil adhesion and migration.

References:

Ablain, J., E.M. Durand, S. Yang, Y. Zhou, and L.I. Zon. 2015. A CRISPR/Cas9 vector system for tissue-specific gene disruption in zebrafish. Developmental cell. 32:756-764.
Amini, P., D. Stojkov, A. Felser, C.B. Jackson, C. Courage, A. Schaller, L. Gelman, M.E. Soriano, J.M. Nuoffer, L. Scorrano, C. Benarafa, S. Yousefi, and H.U. Simon. 2018. Neutrophil extracellular trap formation requires OPA1-dependent glycolytic ATP production. Nature communications. 9:2958.
Chen, H., S.A. Detmer, A.J. Ewald, E.E. Griffin, S.E. Fraser, and D.C. Chan. 2003. Mitofusins Mfn1 and Mfn2 coordinately regulate mitochondrial fusion and are essential for embryonic development. The Journal of cell biology. 160:189-200.
de Brito, O.M., and L. Scorrano. 2008. Mitofusin 2 tethers endoplasmic reticulum to mitochondria. Nature. 456:605-610.
Filadi, R., E. Greotti, G. Turacchio, A. Luini, T. Pozzan, and P. Pizzo. 2015. Mitofusin 2 ablation increases endoplasmic reticulum-mitochondria coupling. Proceedings of the National Academy of Sciences of the United States of America. 112:E2174-2181.
Filadi, R., E. Greotti, G. Turacchio, A. Luini, T. Pozzan, and P. Pizzo. 2017. On the role of Mitofusin 2 in endoplasmic reticulum-mitochondria tethering. Proceedings of the National Academy of Sciences of the United States of America. 114:E2266-E2267.
Kornmann, B., E. Currie, S.R. Collins, M. Schuldiner, J. Nunnari, J.S. Weissman, and P. Walter. 2009. An ER-mitochondria tethering complex revealed by a synthetic biology screen. Science. 325:477-481.
Maianski, N.A., F.P. Mul, J.D. van Buul, D. Roos, and T.W. Kuijpers. 2002. Granulocyte colony-stimulating factor inhibits the mitochondria-dependent activation of caspase-3 in neutrophils. Blood. 99:672-679.
Naon, D., M. Zaninello, M. Giacomello, T. Varanita, F. Grespi, S. Lakshminaranayan, A. Serafini, M. Semenzato, S. Herkenne, M.I. Hernandez-Alvarez, A. Zorzano, D. De Stefani, G.W. Dorn, 2nd, and L. Scorrano. 2016. Critical reappraisal confirms that Mitofusin 2 is an endoplasmic reticulum-mitochondria tether. Proceedings of the National Academy of Sciences of the United States of America. 113:11249-11254.
Naon, D., M. Zaninello, M. Giacomello, T. Varanita, F. Grespi, S. Lakshminaranayan, A. Serafini, M. Semenzato, S. Herkenne, M.I. Hernandez-Alvarez, A. Zorzano, D. De Stefani, G.W. Dorn, 2nd, and L. Scorrano. 2017. Reply to Filadi et al.: Does Mitofusin 2 tether or separate endoplasmic reticulum and mitochondria? Proceedings of the National Academy of Sciences of the United States of America. 114:E2268-E2269.
Rooney, C., G. White, A. Nazgiewicz, S.A. Woodcock, K.I. Anderson, C. Ballestrem, and A. Malliri. 2010. The Rac activator STEF (Tiam2) regulates cell migration by microtubule-mediated focal adhesion disassembly. EMBO reports. 11:292-298.
Saita, S., T. Ishihara, M. Maeda, S. Iemura, T. Natsume, K. Mihara, and N. Ishihara. 2016. Distinct types of protease systems are involved in homeostasis regulation of mitochondrial morphology via balanced fusion and fission. Genes to cells : devoted to molecular & cellular mechanisms. 21:408-424.
Zhou, W., L. Cao, J. Jeffries, X. Zhu, C.J. Staiger, and Q. Deng. 2018. Neutrophil-specific knockout demonstrates a role for mitochondria in regulating neutrophil motility in zebrafish. Disease models & mechanisms. 11.

First decision letter

MS ID#: JOCES/2020/248880

MS TITLE: Mitofusin 2 regulates neutrophil adhesive migration and the actin cytoskeleton

© 2020. Published by The Company of Biologists under the terms of the Creative Commons Attribution License (https://creativecommons.org/licenses/by/4.0/).

AUTHORS: Wenqing Zhou, Alan Y Hsu, Yueyang Wang, Ramizah Syahirah, Tianqi Wang, Jacob Jeffries, Haroon T Mohammad, Xu Wang, David M Umulis, Mohamed N Seleem, and Qing Deng
ARTICLE TYPE: Research Article

Please make the changes in the manuscript that you have outlined in your response to the reviewers. It seems that some of the responses have not been completed. Also, you refer to a Supplemental Figure S1, but no such figure is attached with this manuscript submission. Please ensure that the figure(s) are present when the revised version of the manuscript is resubmitted.

We are aware that you may currently be unable to access the lab to undertake experimental revisions. If it would be helpful, we encourage you to contact us to discuss your revision in greater detail. Please send us a point-by-point response indicating where you are able to address concerns raised (either experimentally or by changes to the text) and where you will not be able to do so within the normal timeframe of a revision. We will then provide further guidance. Please also note that we are happy to extend revision timeframes as necessary.

Please ensure that you clearly highlight all changes made in the revised manuscript. Please avoid using 'Tracked changes' in Word files as these are lost in PDF conversion.

I should be grateful if you would also provide a point-by-point response detailing how you have dealt with the points raised by the reviewers in the 'Response to Reviewers' box. Please attend to all of the reviewers' comments. If you do not agree with any of their criticisms or suggestions please explain clearly why this is so.

First revision

Author response to reviewers' comments

We thank the referees for their enthusiasm and time providing critical feedbacks to our manuscript. The novelty of our work is the identification of the importance of Mfn2 in regulating the Rac signaling and neutrophil migration and adhesion, which is significant to the mitochondrial and the general cell biology field. Below please find our point-to-point response to the comments.

Reviewer #1 (Evidence, reproducibility and clarity (Required)):

Introduction:

"Although mitochondria-derived ATP possibly regulates neutrophil chemotaxis in vitro (Bao et al., 2015), removal of extracellular ATP improves neutrophil chemotaxis in vivo (Li et al., 2016). These conflicting reports prompted us to search for mechanisms delineating the role of mitochondria in neutrophil migration outside the realm of ATP or cellular energy (Bi et al., 2014; Schuler et al., 2017; Zanotelli et al., 2018)."
This sentence is superficial and misleading: extracellular ATP may interfere with chemotaxis through various energy-independent mechanisms (see for example Zumerle et al. Cell Reports 2019) and this is not conflicting with the role of intracellular ATP in migration.

We were not clear in the writing. Bao et al suggests that neutrophils secret ATP at the leading edge and mitochondria at the leading edge is the source of the extracellular ATP. Both studies focused on extracellular ATP. We agree that the reports are not necessarily conflicting with our conclusion. Since exogenous ATP can induce additional signaling. We rewrote this sentence emphasizing that we are looking for mechanisms in addition to ATP, which is distinct from previous studies. The change is now incorporated in the updated manuscript, line 90-97.

Figure 1:
The authors didn't show evidence of the genome edition (PCR, RFLP or Sequencing over the sgRNA

© 2020. Published by The Company of Biologists under the terms of the Creative Commons Attribution License (https://creativecommons.org/licenses/by/4.0/).

target) or at least RT-PCR or WB for MFN2.
In Fig 1b, 1c the scale bar is missing.
"Neutrophils were sorted from both lines and their respective loci targeted by the 4 sgRNAs were deep sequenced." There are no data about sorting strategies for zebrafish neutrophils in the figure. Moreover, only 2 sgRNAs are shown and there are no sequencing data.

To show evidence of the genome edition, we have deep sequenced this loci of *mfn2* and *opa1* and the mutation frequencies were stated in the original text. The sorting strategies were described in Methods-Mutational Efficiency Quantification. Each *mfn2* KO line has 2 individual sgRNAs, and two KO (*mfn2* KO and *mfn2* KO#2) were shown in Fig 1b, so there are 4 sgRNAs targeting *mfn2*. Since each embryos have approximately 150 neutrophils, WB is not feasible. Sequencing is the standard method (Ablain et al., 2015; Zhou et al., 2018). We only stated the mutation efficiency in the manuscript because amplifying the genomic DNA from the sorted cells introduces PCR bias and the numbers are not a quantitative reflection of the degree of gene disruption. We have include the sequencing result of the sgRNA target sites in supplemental Figure 1.

We used one scale bar for all the panels in Fig 1b,c. All panels are at the same magnification.

Figure 2:
In the WB, reconstitution is not obvious. In general, all WBs are not quantified (and they should be quantified).
The in vivo experiment does not have proper controls. For example, can the authors exclude that in these mice there is reduced inflammation because neutrophils have defective activation? What about NETs? And cytokines/chemokines? And exocytosis? In the absence of these controls, the experiment cannot be properly interpreted.

We have now quantified all WBs in our study. We have added the quantifications to each blot. The mice model we chose is used to evaluate in vivo neutrophil migration. We used a neutrophil specific promoter to delete *Mfn2* in mice and collected data at a very early time point when the tissue inflammatory environment is determined by tissue resident sentinel cells, such as macrophages. Although our results support that Mfn2 is required for neutrophil migration in mammals, we agree that we cannot fully rule out that other neutrophil functions are also regulated by Mfn2.

To address whether other neutrophil functions are affected by MFN2, we performed assays to evaluate NETosis and degranulation in MFN2 KD HL-60 cells. The results are now included in Fig. S2 and described in line 163-166.

Figure 3:
The conclusion of the authors "In summary, Mfn2 modulates the actin cytoskeleton and cell migration in MEFs" should be supported by experiments to distinguish between the specific role of Mfn2 and the role of mitochondrial dynamics (Opa1, Drp1, Mfn1). It is also not clear why the authors decided to use MEFs instead of other cells (more similar to neutrophils which are not adherent cells). The results obtained in MEFs may be irrelevant for neutrophils.

We agree that MEFs are very different from neutrophils. We chose MEFs since the function of Mfn2 in MEF is well characterized (Chen et al., 2003; de Brito and Scorrano, 2008; Naon et al., 2016). Both *Mfn1-null* and *Mfn2-null* MEFs have fragmented mitochondria. Mfn1, which is very similar to Mfn2, serves as the best control. We have further confirmed the fragmented mitochondria structure in the KO cells and the results are now included in Fig 3. Related description is added to line 185-187. Our results in MEFs indicate that Mfn2 regulates cell migration not only in fast-migrating cells like neutrophils but also in some slow-migrating cells like MEFs.

For specificity, in addition to Mfn2, we looked at Mfn1 and Opa1 in different systems. We did not select Drp1 since the mitochondrial network in neutrophils is highly fused (Maianski et al., 2002; Zhou et al., 2018).

We have also knocked down OPA 1 in HL-60s. We observed massive cell death in this line and cell migration is affected, possibly due to a depletion of cellular ATP as reported (Amini et al., 2018). We have include the data showing cell death and chemotaxis in Fig S3. The related discussion is added to line 168-171. In zebrafish neutrophils, knocking out opa1 also reduced cell migration (Fig

© 2020. Published by The Company of Biologists under the terms of the Creative Commons Attribution License (https://creativecommons.org/licenses/by/4.0/).

S1C, D).

Figure 4-5:
Fig 5a: in ctrl and sh1 the ER seems to be larger than the phalloidin (=cytoskeleton=cell border approximately) in a few regions. Only the sh1+T seems to fit correctly.

We use the F-actin staining as an indicator of cell front. F-Actin is predominant at cell front, but much less in the cell body and uropod. Here we set the confocal laser power at a certain level to give us a good resolution of brighter signals which may not be strong enough to detect signals in the cell body. That's why the fluorescence is very dim or even absent in the cell body. However, the majority of ER do fit in the cell border if look closer.

The TEM image (only 1 in supplementary) is not sufficient to convince that the tethering is lost. Quantification of number of contacts and distance between ER and mitochondria should be included.

Using EM method for quantification, *Mfn2* ablation decreases the ER-contacting mitochondrial surface by ~20–35% (Naon et al., 2017). Using the same cells, different groups reached different conclusions using TEM(Filadi et al., 2017). We reason that ER-mitochondria contact sites are rare events in TEM since the samples are sectioned. We are not sure that we can come up with a definitive conclusion using TEM. Nevertheless, we observed significant mitochondrial structural changes using IF and observed the changes in cytosolic calcium levels, which is consistent with the known function of Mfn2 as a ER-mitochondrial tether (Naon et al., 2016).

The title of figure 5 is wrong.
However, in these figures, it is clear that cells are beautifully polarized, with mitochondria accumulating at the uropod (and even more in the absence of Mfn2). When comparing these images with those published by Campello et al (JEM 2006), there are 2 observations that can be made: first of all, these data confirm that mitochondrial fission promotes cell polarity; second, they suggest that the defect is not at the level of cell polarity/chemotaxis.

We have corrected the title of Figure 5.

We agree that MFN2 defective neutrophils do not have a defect in cell polarization. The defects in migration is possibly due to other reasons such as poor adhesion or regulation in the actin cytoskeleton dynamics. However, the conclusion that mitochondrial fission promotes cell polarization and chemotaxis cannot be drawn from our data. We have included related discussion in line 204-205.

Figure 6:
Calcium data are, in general, very weak. First of all, controls with ionomycin are missing. Statistical analyses of the curves should be included. As for the use of the MCU inhibitor Ru360, is there any evidence that it is cell-permeant in this context? Is it blocking MCU? Since the authors can show mitochondrial calcium upon FMLP, they should also demonstrate that Ru360 is indeed working and inhibiting mitochondrial calcium uptake.
The sentence "The MCU inhibitor Ru360did not cause further reduction of chemotaxis in MFN2 knockdown dHL-60 cells (Supplementary Fig. 6c, d and Supplementary Movie. 12), indicating that MCU and MFN2 lies in the same pathway in terms of regulating chemotaxis in dHL-60 cells" is speculative.
In general, there is no solid demonstration that the effect is calcium-mediated.

We have performed the control experiment using ionomycin and observed expected results. The data is now added to Fig S7. Statistics are also included.

Ru360 is a widely used MCU inhibitor. The fact that Ru360 itself inhibited neutrophil migration supported that the chemical enters cells. We agree that stating "indicating that MCU and MFN2 lies in the same pathway in terms of regulating chemotaxis in dHL-60 cells" is speculative. In addition, we tried to reduce cytosolic calcium levels in MFN2 KD cells either using Ca^{2+} chelator (BAPTA) or an IP3 receptor inhibitor, 2APB. The data is now included in Fig S7. In both cases we observed reduced migration in control cells. The chemotaxis defect in MFN2 KD cell line was not rescued. This could

© 2020. Published by The Company of Biologists under the terms of the Creative Commons Attribution License (https://creativecommons.org/licenses/by/4.0/).

due to that multiple molecules/pathways are calcium dependent in cell migration. We thus far are still unable to establish a functional link of the calcium with Mfn2 regulated signaling.

The elevated calcium signal is an indirect evidence to support the loss of ER-mitochondria tether. We have modified our conclusion to leave out calcium as a relevant signal regulated by Mfn2 for neutrophil migration.

As for Rac, it is surprising to see that Rac inhibition has no effect on cell migration. Rac is known to promotes migration in fibroblasts and other cell types and Rac deficiency inhibits migration (see for example Steffen et al, JCS 2013). Two sets of experiments are absolutely required: 1) verify this in fibroblasts since it has been elegantly shown that Rac is essential in these cells for migration; 2) analyse the effect of Rac inhibitors in pPak kinetics.

Rac is required for cell migration and the growth of branched actin network. The Rac inhibitors we selected here are specific to two rac GEFs, Vav and Tiam. Steffen et al, JCS 2013 used Rac1 KO MEF, which is different from our approach. Thus the works are not contradictory. MEFs are very different from neutrophils. We chose MEFs since there are knockout cells available and well characterized. The Mfn2 KO cells display prominent lamellipodia, which is also consistent with the observation in Steffen et al, JCS 2013. We have used these two inhibitors in MEFs wound closure and did not observe a defect in cell migration.

We have analyzed the effect of Rac inhibitors in pPAK kinetics in the control and MFN2-deficient dHL-60 cells. Both inhibitors rescued the hyperactivation of RAC in MFN2-deficient dHL-60s but did not affect the RAC activation in control cell line. The data is now included in Fig 7.

Reviewer #1 (Significance (Required)):

As presented here, the manuscript has a modest significance. The audience would be specialised: cell migration, cell signalling.
My expertise is immunology, cell activation, cell migration, cell signalling.

Reviewer #2 (Evidence, reproducibility and clarity (Required)):

Major comments:

Although the results could be very interesting, and could be significantly relevant to the mitochondrial field and the cell biology one in general, major points need to be addressed to fully support conclusions of the authors. Different controls and quantification are missing, Actin dynamics analysis should be improved, effects of the artificial tether is weakly characterized and the demonstration of the specific role of mito-ER contacts via mfn2 in migration should be reinforced.

-In figure 1, quantification of circulating neutrophils is required in Mfn2 KO embryos. The authors should also show these quantified results for OPA1KO, which are just mentioned in the text. In addition, in figure 1b and d, the neutrophils from the Mfn2KO embryos seem bigger compared to control. Can the authors comment on neutrophils size and potential contribution to the phenotype? Finally, the authors propose a defect in neutrophil migration in Mfn2-KO, however neutrophils are found in the circulation. The authors should explain these results.

Since the cells are all in circulation, we can only estimate the percentage. Overall, the phenotype is drastic, as shown in movie S1. From over one hundred neutrophils, only 1 or 2 are in the tissue (mfn2 KO) versus only 1 or 2 in circulation (control or opa1 KO). We have stated how many fish embryos we have imaged in Figure 1 legend and all display the same phenotype. The bigger spots are resulted from cells outside the focal plane-zebrafish embryos are thick tissues. We agree that since neutrophils in the KO fish are all in circulation, we cannot make a conclusion whether they can migrate in tissue in zebrafish. We have performed chemotaxis assays and observed that mfn2-deficient neutrophils fail to respond to inflammation or chemoattractant. Therefore, we conclude that “mfn2 regulates neutrophil tissue retention and extravasation in zebrafish” in line 138.

© 2020. Published by The Company of Biologists under the terms of the Creative Commons Attribution License (https://creativecommons.org/licenses/by/4.0/).

-The authors need to reinforce the Mfn2 specificity for their phenotype. In particular in Fig S1, they show that loss of OPA1 significantly decreases neutrophil migration in vivo. However, they then only study the effect of Mfn1 silencing in neutrophil and MFN1 KO MEFs (Sup Fig s3). The authors should perform the same experiments in neutrophil and MEF upon loss of OPA1 (similar to Fig S3). Does loss of OPA1 and Mfn1 decrease neutrophil arrest to activated endothelial cells?

In addition to knock out opa1 in zebrafish, we knocked down OPA1 in HL-60 cells. The cells appear unhealthy and display a migration defect, consistent with the data in zebrafish. We were not comfortable making conclusions here since secondary affects in dying cells may cause phenotypes not directly attributed to the loss of OPA1. Nevertheless, we have now include the data in Fig S3. The related discussion is added to line 168-171.

We have decided not to include Opa1 KO MEF since the cell morphology as documented in ATCC is similar to that of WT MEF. Only the Mfn2 KO MEF is more circular. Given the structural and functional similarity between Mfn1 and Mfn2, Mfn1 KO MEF is a better specificity control which we have characterized in depth (Fig 3).

Since Mfn1 KD HL-60 cells migrate well on surface, they are not expected to have adhesion defects.

[*NOTE: We have removed images from ATCC documenting the morphology of MEF cells in culture.*

The information can be accessed here:
OPA1 KO MEFs, https://www.atcc.org/products/all/CRL-2995.aspx#characteristics
Mfn2-null MEFs, https://www.atcc.org/products/all/CRL-2993.aspx#characteristics
WT MEFs, https://www.atcc.org/products/all/CRL-2907.aspx#characteristics]

-Using their images, the authors should also document on the directionality of the cell during cell migration. Do Mfn2 depleted cells do not migrate because they are arrested or because they are lacking directionality? Environment/chemokine sensing defects?

We have quantified the directionality of the cells. As pointed out by reviewer 1, MFN2 deficient cells can polarize and are not defective in chemokine sensing. We did not observed any significant changes in directionality. The data is now included in Fig 2C.

-Actin dynamics analysis should be improved. Loss of Mfn1 and Mfn2 lead to cell shape changes. The authors should quantify this phenotype by analysing cell circularity (as well as for Opa1 loss). Stress fibres number or Phalloidin intensity quantification in cell body should also be performed.

We agree with the reviewer and have quantified the circularity, stress fiber numbers and phalloidin intensity in wt, Mfn1 and Mfn2 KO MEFs. The data is now included in Fig 3. The related discussion is added to line 187-192.

-Can the migration defects could be attributed to Focal adhesion protein dynamics defects? The authors shown an hyperactivation of Rac1 and an hyperphosphorylation of PAK, which can control FAP (focal adhesion proteins) dynamics. In addition, immunofluorescence analysis shows a decreased signal and cellular misdistribution of paxillin. The authors should characterize these phenotypes. FAP levels (Paxillin/Phospho-Paxillin and Vinculin) should be analysed by immunoblot, the number of FAP/cell, distribution and size should also be quantified. Their dynamics should also be analysed by live cell imaging. Finally, Paxillin level and distribution seems to be also impacted in Mfn1KO cells. Can the authors comment on that? The different quantifications would help to better understand the effect of different mitofusins in cytoskeleton dynamic.

We thank the reviewer for the great advices for our follow up work. So far our results support Rac over activation as a relevant pathway how Mfn2 regulates neutrophil migration. Although Rac can regulate focal adhesion dynamics in other cells (Rooney et al., 2010), how Rac activation regulates focal adhesion dynamics in neutrophils is not clear. Due to the high background of the Paxillin staining, we have removed the related data from the manuscript. Mfn2-regulated membrane tether could affect lipid trafficking, cellular metabolism and other signaling molecules. It will take

© 2020. Published by The Company of Biologists under the terms of the Creative Commons Attribution License (https://creativecommons.org/licenses/by/4.0/).

substantial amount of work to make a conclusion and it is more suitable to a separate report. This is one of the directions we will pursuit in our future studies.

-Please perform rescue experiments for cell migration in MFN2KO and MFN1KO MEFs. Immunoblots showing protein levels of these proteins would be appreciated. To really discriminate how Mfn2 regulates cell migration, the authors should also perform rescue experiments using a fusogenic mutant Mfn2 ((K109A). It will help to demonstrate the relevance of mito-ER contacts and not mitochondrial fusion in the phenotype.

We thank the reviewer for the suggestions. It is documented that Mfn2 K109A cannot restore mitochondrial fusion. However, it is not clear whether this construct can restore ER-tether. Result using this construct will be hard to interpret. The *Mfn1-null* and *Mfn2-null* MEFs are well characterized cell lines, and we further confirmed the cell lines with fragmentated mitochondria. This work is focused on neutrophils. We have confirmed the function of MFN2 in neutrophils with different rescue strategies including protein rescue, mitochondria-ER artificial tether, and Rac inhibitors.

-Figure 4, the authors stipulate that Mfn2 regulates ER-mitochondria tethering. However, the authors present no evidence for this conclusion. The authors should perform manders coefficient in MFN2 KO cells and compared it to control. Also, loss of Mfn2 induces mitochondrial fragmentation, which can lead to problem for mito-er contacts quantification by light microscopy. The authors should use their TEM pictures to quantify mito-ER contacts (Number, length and % of mito perimeter), not only mitochondrial morphology. Mfn1 should be used as negative control. it would be interesting also to determine the status of the mito-ER contact in the different conditions used in the manuscript to stimulate cell migration like fMLP treatment.

We have performed manders coefficient in the MFN2 KD cells and observed slight but not significant decrease compared with the control. The data is now added to line 213. It is possibly due to the prevalent ER structure in the cells-despite the structural change, mitochondria are still mostly on top of ER when examined using IF. Using EM method, *Mfn2* ablation decreases the ER-contacting mitochondrial surface by ~20–35% (Naon et al., 2017). Using the same cells, different groups reached different conclusions using TEM(Filadi et al., 2017). We reason that ER-mitochondria contact sites are rare events in TEM since the samples are sliced. We are not sure that we will come up with a definitive conclusion by TEM. Nevertheless, we observed significant mitochondrial structural changes using IF and observed the expected changes in cytosolic calcium levels, which is consistent with the known function of Mfn2 as a ER-mitochondrial tether (Naon et al., 2016).

-The authors use an artificial tether to manipulate mito-ER contacts in cellulo. However, no information from its origin, or its design are documented in the manuscript. In addition, the authors should show that this tether efficiently works by analyzing mito-ER contacts upon expression by EM and mitochondrial calcium uptake. Does this tether rescue mito-ER contacts defects induced by loss of Mfn2? How the authors explain that the tether rescues mitochondrial morphology defects in MFN2KO? In these conditions, mitochondria should not be able to fuse anymore as Mfn2 is lost? This is really intriguing results. Does the tether rescue the other parameters? Mitochondrial distribution (with quantification)? Cell shape? Paxillin defects? ROS and membrane potential? These rescue experiments analyses are important to determine which parameters are really involved in cell migration defects due to the decreased tethering. Finally, it would be of great interest to analyse the effect of the tether alone on cell migration, Rac1 activity, cell shape? Gain of function? These results may reinforce the idea that contact sites regulate cell migration.

The tether is a GFP protein carrying both ER and mitochondrial localization sequences at the ends (Kornmann et al., 2009). The details are now added to the manuscript in line 224-226.

In MFN2 KD dHL-60 cells, tether expression partially rescues mitochondrial distribution (quantified in Fig 5C), cell migration and Rac over activation. Although ROS and membrane potential are slightly affected by MFN2 deletion in HL-60 cells, it is not clear whether they play any roles in MFN2 regulated cell adhesion or migration. To confirm that the artificial tether is indeed functional, we have performed calcium measurement in the MFN2 knockdown cells expressing the tether and we observed that the cytosolic calcium level is restored, indicating that the tether is working as

© 2020. Published by The Company of Biologists under the terms of the Creative Commons Attribution License (https://creativecommons.org/licenses/by/4.0/).

expected. The data is now included in Fig 5E. Despite multiple attempts, we could not obtain a line over-expressing the tether in wt HL-60 cells. We suspect that further increase in the tether is toxic to the cells.

-It is well established that a decrease of membrane potential leads to a decrease of mitochondrial calcium uptake. Calcium results obtained by the authors without any information on the roles of the tether could not lead to any conclusion. Does the tether rescues membrane potential and calcium uptake by the mitochondria? So far, the decrease of mitochondrial calcium upon stimulation in Mfn2KO cab be attributed to both mito-ER contact or membrane potential defects. It has been shown that MEFs MFN2 KO can lead to a decrease of MCU provel level leading to a decrease of mitochondrial calcium uptake (PMID: 25870285). The authors should also check MCU protein level.

We observed that mfn2 deficiency resulted in a minor reduction in membrane potential. Although Mfn2 KO MEF has reduced level of Mcu, Mfn2 silence in MEF does not affect Mcu levels (Filadi et al., 2015). Another group also concluded that Mfn2 deletion does not necessarily affect Mcu levels (Naon et al., 2016). We have measured the MCU protein level in the MFN2 knockdown HL-60 cells, and we did not detect a decrease in MCU protein level. The data is now included in Figure S7. The related discussion is added to line 251-252.

-Hyperactivation of Rac1 is only based on phosphorylation of PAK, which is quite weak. The authors should better describe the hyperactivation of RAC1 or other RhoGTPases in their Mfn2 KO MEFs. What are the levels of RAC1 and other RhoGTPases? Subcellular distribution in the cell? Kits are also available to determine RhoGTPase activity by pull down assay (Cell biolabs).

In Mfn2 KO MEFs, Rac overactivation is suggested by the increased lamellipodia formation, which is a classical Rac hyperactivation readout. Since the current manuscript focuses on neutrophils, we performed the Rac-GTP pull down experiments in HL-60 cells. Consistently, significantly more Rac-GTP was detected in MFN2-deficient dHL-60s compared to the control dHL-60 cells. We also stained Rac GTP in different dHL-60 cells. The data is now included in Fig 6.

-The references are up-to-date. The text and the figures are clear and accurate.

Minor comments:

-The authors should show the efficiency of the KO generated for Mfn2 and Opa1 in zebrafish embryos. Sequencing results to highlight the position of the mutations and their consequences on the coding protein should be shown, as well as immunoblot analysis should be performed to analyse Mfn1, Mfn2 and OPA1 protein levels. The generation of a MFN1-KO transgenic line would have been appreciated to finely compare the roles of the 3 GTPases involved in mitochondrial fusion during neutrophil infiltration and migration in vivo.

To show evidence of the genome editing, we have deep sequenced the loci of *mfn2* and *opa1* and the mutation frequencies were stated in the original text. Since each embryos have approximately 150 neutrophils, WB is not feasible. Sequencing is the standard method (Ablain et al., 2015; Zhou et al., 2018). We only stated the mutation efficiency in the manuscript because amplifying the genomic DNA from the sorted cells introduces PCR bias and the numbers are not a quantitative reflection of the degree of gene disruption. We have included the sequencing result of the sgRNA target sites in Figure S1.

The *mfn1* gene in zebrafish is duplicated. We have generated an mfn1b KO line and did not observe any phenotype. However we are not sure whether we can obtain efficient disruption at both mfn1a and mfn1b loci. We have compared the function of Mfn1 in cell migration by using Mfn1 KO MEF and MFN1 KD HL-60 cells, showing a specific role of Mfn2 in cell migration.

-MFN1, MFN2, AND OPA1 protein levels should be analysed by immunoblot in the Mfn1 and Mfn2 KO MEFs.

It is unlikely that mfn1/2 KO will affect OPA1 levels (Saita et al., 2016). Both Mfn1 KO and Mfn2 KO MEFs display fragmented mitochondrial network which can be rescued by overexpression of Mfn1 or

© 2020. Published by The Company of Biologists under the terms of the Creative Commons Attribution License (https://creativecommons.org/licenses/by/4.0/).

Mfn2 (Chen et al., 2003). The level of Opa1 in the cells are not relevant. We have stained mitochondria in the Mfn1/2 KO MEFs and confirmed that the cells have fragmented mitochondria as expected. The data is now added to Fig. 3. Related description is added to line 186-187.

-In cell spreading assay, it would be great to identify cells during the process, by an asterix for example. "wt MEFs extended transient filopodia and lamellipodia and eventually elongated, whereas Mfn2-null MEFs generated extensive membrane ruffles and retained the circular shape". It would be interesting to quantify these different parameters.

We thank the reviewer for the suggestions and have added Asterixes to the cells. We have also quantified the percentage of cells that can rearrange their cell shape in the WT, Mfn1, and Mfn2 KO MEFs. The results are included in Fig 3 and results are described in line 193-196.

-For all their immunoblot analysis, the authors should use a mitochondrial marker as loading control (VDAC1, TOM20, HSP60...). In figure 5, Vinculin should not be used a loading control, with its role in focal adhesion dynamics.

Vinculin is stable in HL-60 cells under multiple conditions and thus was selected as a control. The signal intensity correlates well with the amount of protein loaded. Using mitochondrial proteins as loading controls is not common and may be risky as the amount of mitochondria in cells can be variable.

-Legends for figures 5 and 6 are inverted.

Thanks, we have changed the heading of figure 5. The legends were corrected.

-Please document in the material and methods section, how confocal images have been acquired: number of z-stacks, reconstitution, 3D analysis...

We have updated in the method the parameters of imaging acquisition.

-The authors should show their results of blood cell composition quantification in ctrl vs MFn2 depletion.

We have include the results of blood cell composition in supplemental figure 2 L and M.

-The authors should describe all the acronyms used throughout all the manuscript. For example, LTB4, fMLP...

We have describe all the acronyms in the updated manuscript.

Reviewer #2 (Significance (Required)):

Beyond their role in energy production, mitochondria are involved in numerous cellular functions including cell migration. Mitochondria form a network balanced by fission and fusion events, where membrane contact sites with the endoplasmic reticulum are crucial. These contact sites are also involved in mitochondrial and cellular functions via their capacity to exchange lipids, metabolites and calcium. The role of mitochondria in cell migration has started to emerge where mitochondrial fragmentation and/or mitochondrial calcium homeostasis are acknowledged to drive cancer cell migration and to regulate actin dynamics.
In this manuscript, Zhou W and colleagues proposed for the first time the role of mitochondria-ER contacts in cell migration. Mechanistically, this can be associated to the capacity of these contacts to control mitochondrial functions or mitochondrial calcium homeostasis. These findings are physiologically relevant and of particular interest to the mitochondrial and cell migration field but also to general cell biology. It represents a novel function associated to these membrane contact sites and point-out these contacts as signalling platform creating microdomains of metabolites exchanges involved in cell migration.

Keywords: Mitochondria - Membraned dynamics - calcium homeostasis - Membrane contact sites

© 2020. Published by The Company of Biologists under the terms of the Creative Commons Attribution License (https://creativecommons.org/licenses/by/4.0/).

Reviewer #3 (Evidence, reproducibility and clarity (Required)):

Mitofusin 2 (Mfn2) is a mitochondrial outer membrane protein that is important for mitochondrial fusion and the establishment of mitochondrial ER contacts. It has been published before that these contact sites are important for calcium signaling. Zhou et al. examined the role of Mfn2 in neutrophils. They propose a model in which mitochondrial ER contacts established via Mfn2 are crucial for regulation of Rac, which is a small GTPase driving cell migration by promoting actin polymerization. Loss of Mfn2 results in elevated cytosolic calcium, over-activation of Rac, and defects of chemotactic movements. These defects can be partially rescued by restoration of mitochondrial ER contacts through expression of an artificial tether protein.

Major points

1.The authors claim on p. 6 that decreased neutrophil retention is not simply due to defects in mitochondrial fusion. However, the experimental setup they used for mfn2 (Fig. 1) is different from that for opa1 (Fig. S1), and therefore the results are not directly comparable. Unfortunately, the authors don't show fragmentation of mitochondria, neither in mfn2 nor in opa1 depleted cells. To support their statement they must show that lack of Mfn2 and Opa1 causes mitochondrial fragmentation to the same extent and then examine neutrophil retention in the same assay. Also, it would make sense to include Mfn1 in this analysis, as the authors later claim that the effects they observed are specific for Mfn2.

Since mfn2 KO neutrophils are not in tissue, the experiment in Fig S1 to look at cell speed in tissue is not feasible. Since the cells are all in circulation, we can only estimate the percentage. Overall, the phenotype is drastic, as shown in movie S1. From over one hundred neutrophils, only 1 or 2 are in the tissue (mfn2 KO) versus only 1 or 2 in circulation (control or opa1 KO). We have stated how many fish embryos we have imaged in Figure 1 legend and all display the same phenotype.

To show evidence of the genome editing, we have deep sequenced this loci of mfn2 and opa1 and the mutation frequencies were stated in the original text. Since each embryos have approximately 150 neutrophils, WB and other biochemical assays are not feasible. Sequencing is the standard method (Ablain et al., 2015; Zhou et al., 2018). We only stated the mutation efficiency in the manuscript because amplifying the genomic DNA from the sorted cells introduces PCR bias and the numbers are not a quantitative reflection of the degree of gene disruption. We have included the sequencing result of the sgRNA target sites in Figure S1.

Since Mfn2 KO neutrophils are all in circulating, we cannot observe their mitochondrial morphology. This is the reason why we used HL-60 cells for the mechanistic study. The mfn1 gene in zebrafish is duplicated. We have generated an mfn1b KO line and did not observe any phenotype. However we are not sure whether we can obtain efficient disruption at both mfn1a and mfn1b loci. We have compared the function of Mfn1 in cell migration by using Mfn1 KO MEF and MFN1 KD HL-60 cells. The results indicated a specific role of Mfn2 in cell migration.

We have stained the mitochondria structure in the Mfn1/2 MEF cells and the results are now included in Fig 3. We have also stained the mitochondria structure in MFN1/2 KD dHL-60 cells, the results are now included in Fig 4 and Fig S3. Indeed, unlike in MEFs, a significant increase in mitochondria fragmentation was not observed in either the MFN1 or MFN2 knockdown dHL-60 cells. We therefore modified our conclusion accordingly in line 301-306: It remains to be determined whether mitochondrial fission/fusion regulates neutrophil migration. The fused mitochondrial network in neutrophils is possibly a result of the abundant expression of the mitofusins. Evaluating the role of mitochondrial shape regulating genes in neutrophil migration will be necessary to draw a solid conclusion on this topic.

We have also knocked down OPA 1 in HL-60s. We observed massive cell death in this line and cell migration is affected, possibly due to a depletion of cellular ATP as reported (Amini et al., 2018). We have include the data showing cell death and chemotaxis in Fig S3. The related discussion is added to line 168-171. In zebrafish neutrophils, knocking out Opa1 also reduced cell migration (Fig S1).

© 2020. Published by The Company of Biologists under the terms of the Creative Commons Attribution License (https://creativecommons.org/licenses/by/4.0/).

2.The authors should examine mitochondrial morphology in MFN2 shRNA treated cells (Fig. 2) and in mfn2-null MEFs (Fig. 3).

We agree with the reviewer and have examined mitochondrial morphology in MFN2-deficient dHL-60 cells (Fig 4c and 5a). The mitochondrial morphology in *Mfn2-null* MEFs has been well characterized (Chen et al., 2003). We have confirmed their results by staining mitochondrial structure in the KO MEFs. The results are now included in Fig 3. Related description is added to line 185-187.

3.The authors claim that chemotaxis defects of neutrophils are specific for MFN2 knock down, but not for MFN1. They show a Western blot of mfn1 knock down cells in Figure S3s. There is a band clearly visible, which appears to be much stronger than the MFN2 band in sh1 cells in Fig. 2a. Therefore, this conclusion is not valid.

The band intensities are dependent on the antibody quality and imaging acquisition and display. We don't feel comfortable comparing the amount of two different proteins from two separate blots.

4.The colocalization of MFN2 with mitochondria and ER, shown in Fig. 4a, should be improved. Both mitochondria and ER appear abnormally clumped. The authors should stain mitochondria, ER and Mfn2 in the same cells. Images should be displayed much larger. The same is true for Fig. 5a. The authors claim that an artificial tether restored mitochondrial morphology in mfn2 knock down cells. They should state in the text which tether was used. Furthermore, they should explain their criteria for judgement of mitochondrial morphology. At least in my exes, mitochondria appear highly clumped in the image shown for sh1+T cells. In Fig. 5c it is not indicated how many cells were scored.

We have replaced Fig 4a with a more representative image.

Neutrophils are blood cells and do not spread as well as adherent cells. We have also overexposed the images to show the smaller mitochondria, which cannot be visualized without saturating the signals. We tried to stain the cells with Mfn2 and Calnexin. However we cannot retain the mitotracker signal in fixed cells and could not do a triple label in dHL-60 cells. For this reason we have done double staining of mitochondria-ER, MFN2-mitochondria and MFN2-ER.

We have included the citation and the description of the tether in line 224-227. The tether is composed of a GFP protein carrying both ER and mitochondrial localization sequences at the ends, which functions independently of MFN2.

The criteria for the judgement of the mitochondrial morphology is now included in the methods, confocal imaging.

Minor points

5.The Western blot shown in Fig. 5d suggests that expression of the tether construct reduced the amount of MFN2. How can this be explained?

This Mfn2 amount is not significantly altered by the tether expression when quantified. We have add the quantification of all WB to the figures.

6.The paper is sometimes hard to digest for a reader who is not familiar with the authors' experimental systems. The description of the experiments in the main text is highly condensed.

We thank the reviewer for the suggestions and have elaborated on the experimental system in the results section.

7.Page 11: "5 m post stimulation" should read "5 min post stimulation".

© 2020. Published by The Company of Biologists under the terms of the Creative Commons Attribution License (https://creativecommons.org/licenses/by/4.0/).

We thank the reviewer for this point. We have made this correction.

8.Some references are incomplete (page numbers are lacking).

We have reformat our references and checked for page numbers.

Reviewer #3 (Significance (Required)):

Apparently, the manuscript is written for an audience with a special interest in chemotactic movements of neutrophils. I guess that the results reported in this manuscript will be of interest for this field. My background is mitochondrial biology and dynamics and I don't have the expertise to evaluate the aspects specific for neutrophils.

It is well established that mfn2 mediates mitochondrial fusion and mitochondria-ER contact. Our novelty is the identification that mfn2 suppresses Rac activation, which is essential for neutrophil adhesion and migration.

References:

Ablain, J., E.M. Durand, S. Yang, Y. Zhou, and L.I. Zon. 2015. A CRISPR/Cas9 vector system for tissue-specific gene disruption in zebrafish. Developmental cell. 32:756-764.

Amini, P., D. Stojkov, A. Felser, C.B. Jackson, C. Courage, A. Schaller, L. Gelman, M.E. Soriano, J.M. Nuoffer, L. Scorrano, C. Benarafa, S. Yousefi, and H.U. Simon. 2018. Neutrophil extracellular trap formation requires OPA1-dependent glycolytic ATP production. Nature communications. 9:2958.

Chen, H., S.A. Detmer, A.J. Ewald, E.E. Griffin, S.E. Fraser, and D.C. Chan. 2003. Mitofusins Mfn1 and Mfn2 coordinately regulate mitochondrial fusion and are essential for embryonic development. The Journal of cell biology. 160:189-200.

de Brito, O.M., and L. Scorrano. 2008. Mitofusin 2 tethers endoplasmic reticulum to mitochondria. Nature. 456:605-610.

Filadi, R., E. Greotti, G. Turacchio, A. Luini, T. Pozzan, and P. Pizzo. 2015. Mitofusin 2 ablation increases endoplasmic reticulum-mitochondria coupling. Proceedings of the National Academy of Sciences of the United States of America. 112:E2174-2181.

Filadi, R., E. Greotti, G. Turacchio, A. Luini, T. Pozzan, and P. Pizzo. 2017. On the role of Mitofusin 2 in endoplasmic reticulum-mitochondria tethering. Proceedings of the National Academy of Sciences of the United States of America. 114:E2266-E2267.

Kornmann, B., E. Currie, S.R. Collins, M. Schuldiner, J. Nunnari, J.S. Weissman, and P. Walter. 2009. An ER-mitochondria tethering complex revealed by a synthetic biology screen. Science. 325:477-481.

Maianski, N.A., F.P. Mul, J.D. van Buul, D. Roos, and T.W. Kuijpers. 2002. Granulocyte colony-stimulating factor inhibits the mitochondria-dependent activation of caspase-3 in neutrophils. Blood. 99:672-679.

Naon, D., M. Zaninello, M. Giacomello, T. Varanita, F. Grespi, S. Lakshminaranayan, A. Serafini, M. Semenzato, S. Herkenne, M.I. Hernandez-Alvarez, A. Zorzano, D. De Stefani, G.W. Dorn, 2nd, and L. Scorrano. 2016. Critical reappraisal confirms that Mitofusin 2 is an endoplasmic reticulum-mitochondria tether. Proceedings of the National Academy of Sciences of the United States of America. 113:11249-11254.

Naon, D., M. Zaninello, M. Giacomello, T. Varanita, F. Grespi, S. Lakshminaranayan, A. Serafini, M. Semenzato, S. Herkenne, M.I. Hernandez-Alvarez, A. Zorzano, D. De Stefani, G.W. Dorn, 2nd, and L. Scorrano. 2017. Reply to Filadi et al.: Does Mitofusin 2 tether or separate endoplasmic reticulum and mitochondria? Proceedings of the National Academy of Sciences of the United States of America. 114:E2268-E2269.

Rooney, C., G. White, A. Nazgiewicz, S.A. Woodcock, K.I. Anderson, C. Ballestrem, and A. Malliri. 2010. The Rac activator STEF (Tiam2) regulates cell migration by microtubule-mediated focal adhesion disassembly. EMBO reports. 11:292-298.

Saita, S., T. Ishihara, M. Maeda, S. Iemura, T. Natsume, K. Mihara, and N. Ishihara. 2016. Distinct types of protease systems are involved in homeostasis regulation of mitochondrial morphology via balanced fusion and fission. Genes to cells : devoted to molecular & cellular mechanisms. 21:408-424.

© 2020. Published by The Company of Biologists under the terms of the Creative Commons Attribution License (https://creativecommons.org/licenses/by/4.0/).

Zhou, W., L. Cao, J. Jeffries, X. Zhu, C.J. Staiger, and Q. Deng. 2018. Neutrophil-specific knockout demonstrates a role for mitochondria in regulating neutrophil motility in zebrafish. Disease models & mechanisms. 11.

Second decision letter

MS ID#: JOCES/2020/248880

MS TITLE: Mitofusin 2 regulates neutrophil adhesive migration and the actin cytoskeleton

AUTHORS: Wenqing Zhou, Alan Y Hsu, Yueyang Wang, Ramizah Syahirah, Tianqi Wang, Jacob Jeffries, Haroon T Mohammad, Xu Wang, David M Umulis, Mohamed N Seleem, and Qing Deng
ARTICLE TYPE: Research Article

Thank you for sending your manuscript to Journal of Cell Science through Review Commons. I have gone through the revised manuscript and your point-by-point response and am happy to tell you that your manuscript has been accepted for publication in Journal of Cell Science, pending standard ethics checks.

© 2020. Published by The Company of Biologists under the terms of the Creative Commons Attribution License (https://creativecommons.org/licenses/by/4.0/).
